# Copper-Catalyzed Borylation of Styrenes by 1,8-Diaminonaphthalene-Protected Diboronic Acid

**DOI:** 10.1021/acs.orglett.3c00451

**Published:** 2023-03-22

**Authors:** Taiga Yasuda, Yusuke Yoshigoe, Shinichi Saito

**Affiliations:** Department of Chemistry, Faculty of Science, Tokyo University of Science, Kagurazaka, Shinjuku, Tokyo 162-8601, Japan

## Abstract

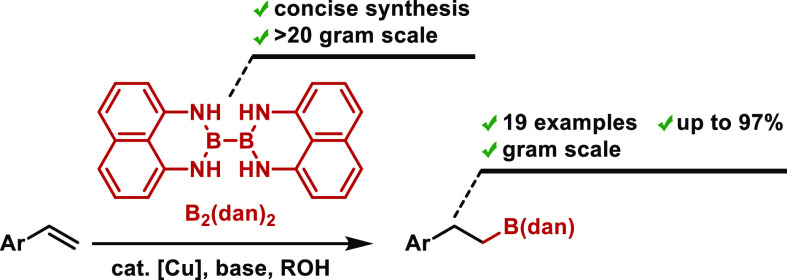

We report a concise synthesis of 1,8-diaminonaphthalene-protected diboronic acid (B_2_(dan)_2_), which is a promising borylating agent. B_2_(dan)_2_ is a bench-stable compound, and it could be utilized for Cu-catalyzed borylation of styrene derivatives. The reaction proceeded in a highly selective manner, and the products were isolated in up to 97% yields. Mechanistic studies revealed that a borate species would be a key intermediate for the borylation reaction.

Boronic acid derivatives are important compounds in synthetic organic chemistry because they are useful and less toxic substrates for various coupling reactions.^1^ Among them, 1,8-diaminonaphthalene (H_2_-dan)-protected boronic acids (R-B(dan)) are known as stable derivatives, which do not react under standard conditions for Suzuki–Miyaura coupling or undergo protodeboronation under aqueous basic conditions.^2^ R-B(dan) could be, however, converted to reactive boronic acid (R-B(OH)_2_) by acidic hydrolysis. Accordingly, R-B(dan) has been used as a protected boronic acid in organic synthesis. Recently the usefulness of these compounds is increasing since we and other groups examined the reactivity of alkynyl-B(dan),^3a^ alkenyl-B(dan),^3b^ aryl-B(dan),^3c,3d^ alkyl-B(dan),^2f^ and cyclopropyl-B(dan)^3e^ and found that these compounds could be directly employed as substrates for cross coupling reactions.

Though the syntheses of alkenyl-B(dan), aryl-B(dan), and alkynyl-B(dan) have been well documented, fewer methods are available for the synthesis of alkyl-B(dan).^4^ Among the methods reported for the synthesis of alkyl-B(dan), we were interested in the copper-catalyzed borylation of alkenes with (pin)B–B(dan) (pin: pinacolato, Scheme 1a).^4a^ Though this reaction was reported in 2014 by Yoshida et al., only two examples have been described, and the generality of the reaction is unclear. Moreover, the chemistry of closely related reactions using B_2_(pin)_2_ has been extensively studied by Hoveyda and other groups.^5^ Based on these results, we anticipated that B_2_(dan)_2_ would be a suitable substrate for this type of transformation. In this paper we report an improved synthesis of B_2_(dan)_2_ and its application to the copper-catalyzed borylation of styrene derivatives (Scheme 1b).

**Scheme 1:**
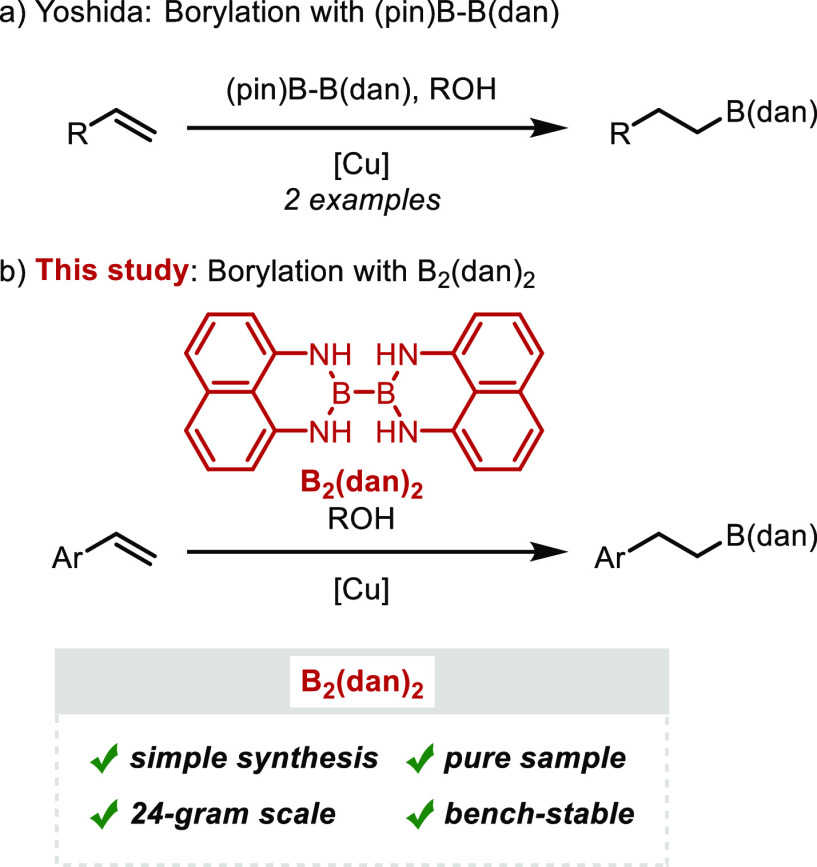
Synthesis of Alkyl-B(dan) with Diboron Reagents

A reported method for the synthesis of B_2_(dan)_2_ involves the reaction of 1,8-diaminonaphthalene with B_2_(NMe_2_)_4_.^6,7^ We envisioned that B_2_(dan)_2_ could be synthesized by the condensation of 1,8-diaminonaphthalene with commercially available tetrahydroxydiboron (B_2_(OH)_4_, Scheme 2). When a mixture of 1,8-diaminonaphthalene and B_2_(OH)_4_ in toluene was refluxed with a Dean–Stark trap for 4 h, the dehydration proceeded, and B_2_(dan)_2_ was isolated as white powder in 92% yield. The synthesis of B_2_(dan)_2_ could be achieved at a large scale (79 mmol of **1**) to provide 24 g of the product. B_2_(dan)_2_ was a bench-stable, white solid, and it could be purified by silica gel column chromatography or recrystallization (AcOEt). It was important to purify 1,8-diaminonaphthalene by sublimation before use: if commercial 1,8-diaminonaphthalene was used without purification, B_2_(dan)_2_ was isolated as a colored solid.^8^

**Scheme 2:**
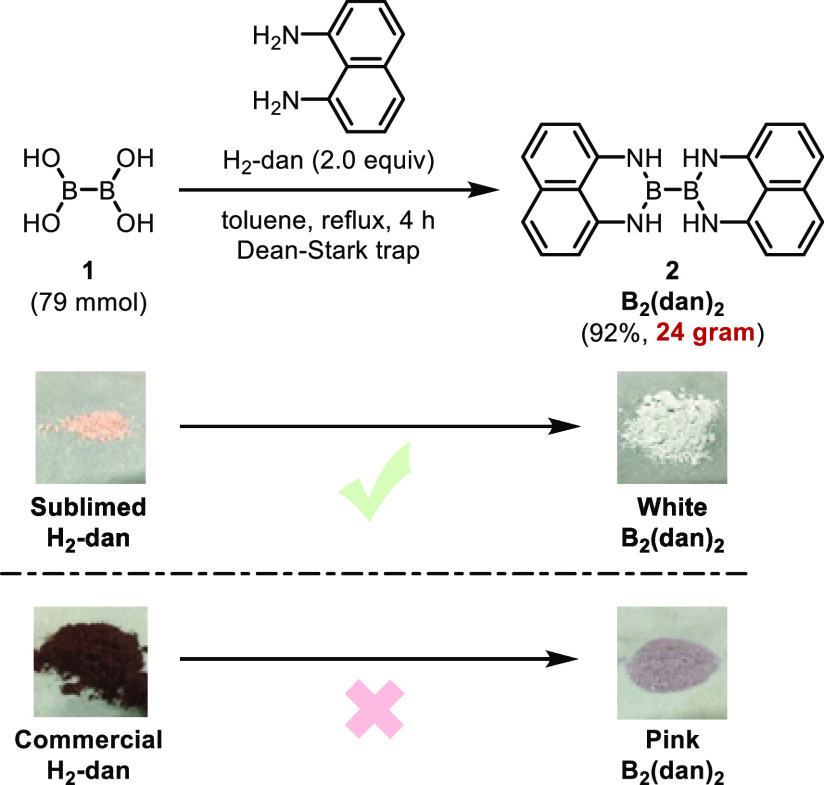
Improved Synthesis of B_2_(dan)_2_

Next, we examined the borylation reaction of unsaturated hydrocarbons using B_2_(dan)_2_ as the borylating agent. After brief screening of the reaction conditions, we found that B_2_(dan)_2_ could be used for the copper-catalyzed borylation reaction of styrenes (Table 1). Thus, borylation of 4-methoxystyrene (**3a**, 1.0 equiv) with B_2_(dan)_2_ (1.2 equiv) proceeded in the presence of CuBr_2_, dppp (5 mol %), KO^*t*^Bu (0.50 equiv), and HO^*t*^Bu (1.2 equiv) in 1,4-dioxane at 50 °C for 2 h, and **4a** was isolated in 55% yield (entry 1). The reaction proceeded regioselectively, and only β-borylated product was isolated. When dppf was used as a ligand, **4a** was obtained in a better yield (entry 2). Among the phosphine ligands we tested, Xantphos was most effective: the reaction of **3a** completed in 2 h, and the product was isolated in 96% yield (entry 3). The results may imply that a phosphine ligand with a large bite angle could have a favorable effect on the progress of the reaction. We next examined the catalytic activity of NHC (N-heterocyclic carbene)–copper complexes. While the catalytic activity of CuCl(IPr) was very low, CuCl(IMes) catalyzed this reaction with high efficiency (entries 4 and 5). Among the complexes we tested, CuCl(SIMes) was the most efficient catalyst for this reaction: the reaction completed in 1 h, and the product was isolated in 95% yield (entry 6). Finally, we briefly examined the solvent effect on the reaction. Though THF turned out to be an inferior solvent, the reaction proceeded smoothly in toluene, and the product was isolated in 97% yield (entries 7 and 8).^9^ The yield of the product decreased when a smaller amount (0.25 equiv) of KO^*t*^Bu was used (entry 9). Based on these results, we selected the reaction conditions described in entry 8 as the best conditions of this reaction.^10^ We confirmed that the reaction could be scaled up without problem: when a larger amount (4.0 mmol) of **3a** was used for this reaction, **4a** was isolated in 92% yield (entry 8).

**Table 1:**
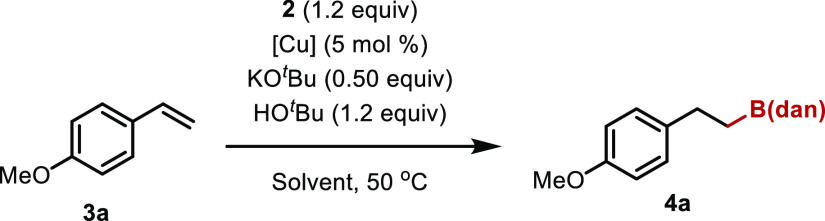
Optimization of the Reaction Conditions^a^

Entry	[Cu]^b^	Solvent	*t* (h)	Yield^c^ (%)
1	CuBr_2_ + dppp	1,4-dioxane	2	55
2	CuBr_2_ + dppf	1,4-dioxane	2	70
3	CuBr_2_ + Xantphos	1,4-dioxane	2	96
4	CuCl(IPr)	1,4-dioxane	2	Trace
5	CuCl(IMes)	1,4-dioxane	2	90
6	CuCl(SIMes)	1,4-dioxane	1	95
7	CuCl(SIMes)	THF	1	49
8	CuCl(SIMes)	toluene	1	97 (92)^d^
9	CuCl(SIMes)	toluene	1	86^e^

a A mixture of **2** (1.2 equiv), [Cu] (5 mol %), KO^*t*^Bu (0.50 equiv), **3a** (0.30 mmol), and HO^*t*^Bu (1.2 equiv) in solvent (0.85 mL) was stirred for 1–2 h at 50 °C.

b dppp = 1,3-bis(diphenylphosphino)propane; dppf = 1,1′-bis(diphenylphosphino)ferrocene; Xantphos = 4,5-bis(diphenylphosphino)-9,9-dimethylxanthene.

c Isolated yield.

d A larger amount (4.0 mmol) of **3a** was used.

e A smaller amount (0.25 equiv) of KO^*t*^Bu was used.
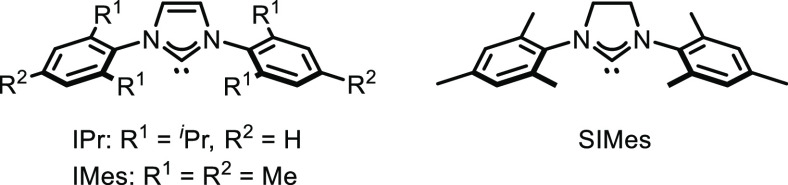

With the optimal conditions in hand, we investigated the substrate scope of this reaction (Scheme 3). Unsubstituted styrene (**3b**) and styrene derivatives with an electron-donating group (**3c**–**f**) were highly reactive: the reaction of 4-alkylstyrene derivatives completed in 1 h, and corresponding alkyl-B(dan) were isolated in 92–96% yields. 4-(*N*,*N*-Dimethylamino)styrene (**3e**) was also a suitable substrate for this borylation. It is noteworthy that the *t*-butyldimethylsilyl (TBS) group was unaffected when the reaction of a TBS-protected styrene (**3f**) was examined. When 4-chloro or 4-bromostyrene was used, the progress of the reaction was slow: a longer reaction time (3 h) was necessary for the completion of the reaction. The lower yield of **4h** might be attributed to the higher reactivity of the bromide under the reaction conditions. 4-Trifluoromethyl and 4-*t*-butoxycarbonylstyrene were fairly reactive substrates, and the borylated products (**4i** and **j**) were isolated in 66–75% yields. The progress of the reaction of 4-cyanostyrene (**3k**) was very slow. The product (**4k**) was isolated in 50% yield after heating the reaction for 24 h. We assume that the progress of the reaction was inhibited by the coordination of the cyano group of the substrate to the copper catalyst. The reactivity of 3- or 2-methoxystyrene was comparable to that of 4-methoxystyrene, and the products (**4l** and **4m**) were isolated in high yields. 2-Vinylnaphthalene (**3n**) was a good substrate, and the reaction proceeded smoothly.

**Scheme 3:**
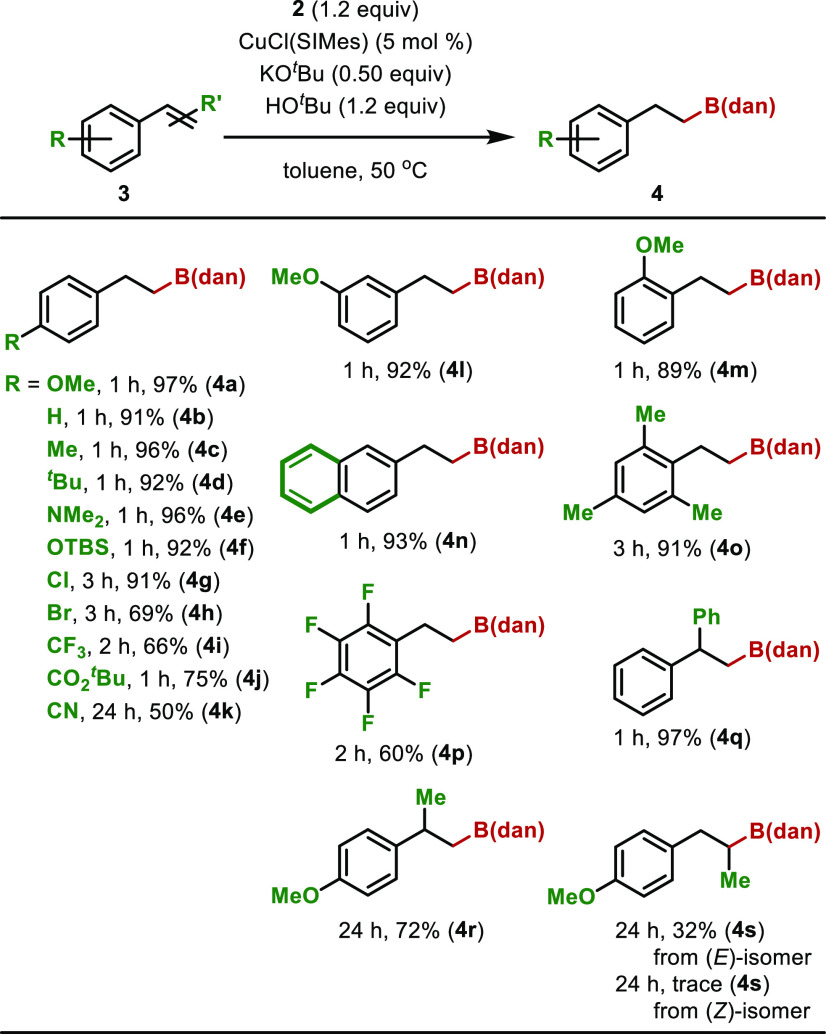
Substrate Scope A mixture of **2** (1.2 equiv), CuCl(SIMes) (5 mol %), KO^*t*^Bu (0.50 equiv), **3** (0.30 mmol), and HO^*t*^Bu (1.2 equiv) in toluene (0.85 mL) was stirred at 50 °C.

We further examined the reaction of multiply substituted styrenes such as 2,4,6-trimethylstyrene and 2,3,4,5,6-pentafluorostyrene, and the corresponding borylated products were isolated (**4o** and **4p**), though a longer reaction time (2–3 h) was required. The reactivity of 1,1-diphenylethene was comparable to styrene, and the borylation proceeded smoothly; however, the reaction of α-methyl-4-methoxystyrene was slow, and a longer reaction time (24 h) was required for the completion of the reaction. The reactivity of β-methyl-4-methoxystyrene was very low. The product (**4s**) was isolated in 32% yield after heating the (*E*)-isomer for 24 h, while a trace amount of **4s** was detected in the ^1^H NMR spectrum of the crude mixture when the (*Z*)-isomer was treated under identical reaction conditions.^11,12^ The decreased reactivity of disubstituted alkenes in these examples may imply that the interaction of the copper species with the alkene was inhibited by introducing an alkyl group to the olefinic moiety.

The usefulness of the alkyl-B(dan) derivative was demonstrated by the conversion of **4a** into some derivatives (Scheme 4). For example, iodide **5** was synthesized in 75% yield by the reaction of **4a** with KO^*t*^Bu and I_2_.^13^ The treatment of **4a** with KO^*t*^Bu in toluene under air at 80 °C gave alcohol **6** in 68% yield.^14,15^

**Scheme 4:**
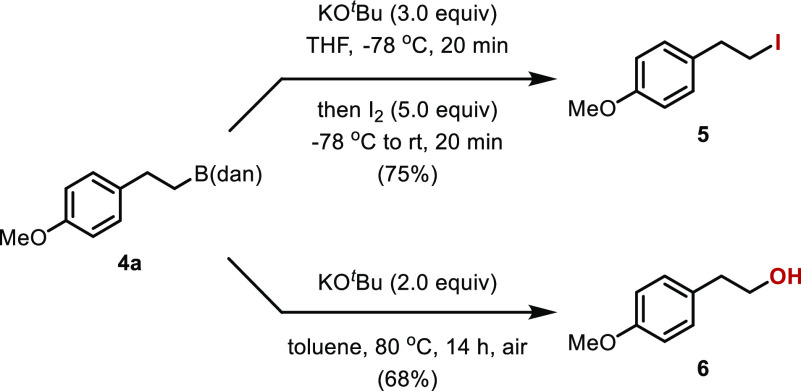
Reactions of **4a**

To understand the mechanism of this reaction, we conducted some control experiments and attempted to detect key organoboron intermediates (Scheme 5). When 5 mol % of KO^*t*^Bu, which is sufficient for the formation of the copper butoxide complex, was employed, the reaction did not proceed (Scheme 5a). To probe the role of KO^*t*^Bu, we compared the ^11^B{^1^H} NMR chemical shift of B_2_(dan)_2_ and a mixture of B_2_(dan)_2_ and KO^*t*^Bu. The ^11^B{^1^H} NMR chemical shift of B_2_(dan)_2_ appeared at 31.5 ppm in 1,4-dioxane, while a new signal appeared at 0.98 ppm when KO^*t*^Bu (1.0 equiv) was added to the solution of B_2_(dan)_2_ (Scheme 5b). Since the observed chemical shift was similar to the values (−0.96 ppm^3c^ and −1.1 ppm^3d^) of the borate formed by the reaction of Ph-B(dan) with KO^*t*^Bu and the reported values (ca. 0 ppm) of other borates,^3e^ we assume that a borate was formed by the reaction of B_2_(dan)_2_ with KO^*t*^Bu. Next, deuterated B_2_(dan)_2_ and MeOD were used for the reaction, and deuterated alkyl-B(dan) (**4a**-*d*, 84% yield, 48% D) was obtained (Scheme 5c). The isolation of **4a-***d* implies that a benzylcopper species was formed as an intermediate of this reaction.

**Scheme 5:**
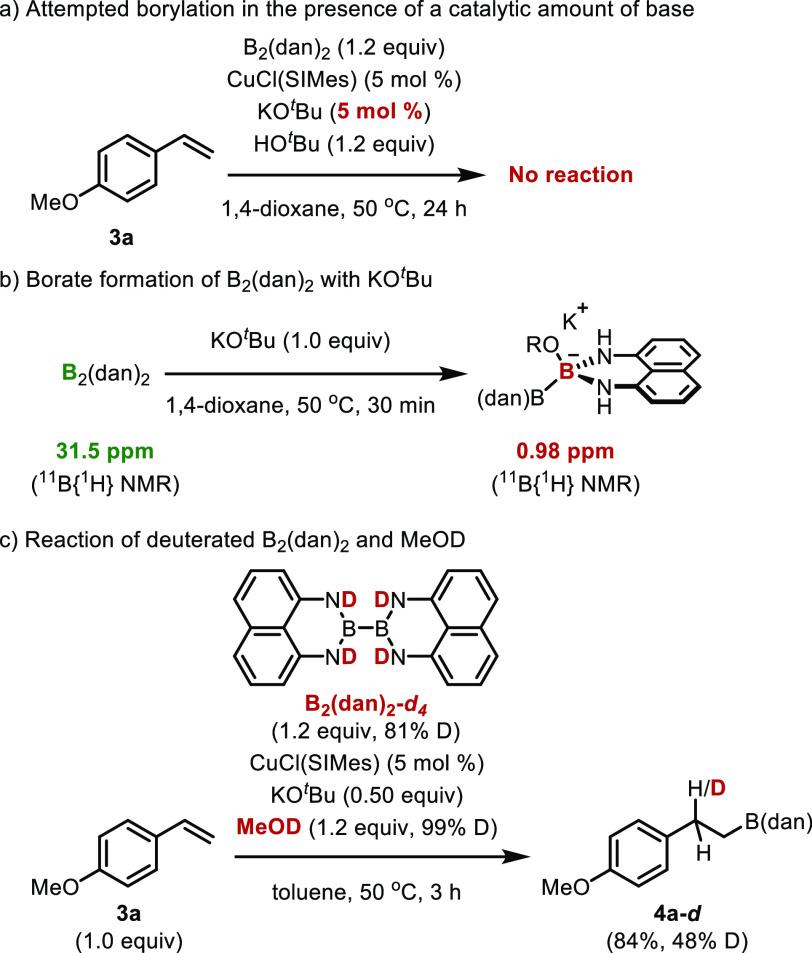
Mechanistic Study

Based on these results, we assume that the mechanism of this reaction would be similar to a generally accepted pathway^5,16^ for the copper-catalyzed borylation reaction of alkene by diboron compounds (Scheme 6). Copper–alkoxide **I** was formed by the reaction of CuCl(SIMes) and KO^*t*^Bu, and **I** would react with borate **II** to form [Cu]–B(dan) **III**.^17^ Complex **III** would interact with styrene to yield a π-complex (**IV**). The insertion of the Cu–B bond to C=C bond would proceed, and alkylcopper species **V** would be generated. The high selectivity of this process would be explained in terms of the formation of a stable η^3^-benzylcopper species^16b^ and/or the bulkiness of the B(dan) moiety.^18^ Protonation of the organocopper species by ROH would provide the final product with concomitant regeneration of **I**.

**Scheme 6:**
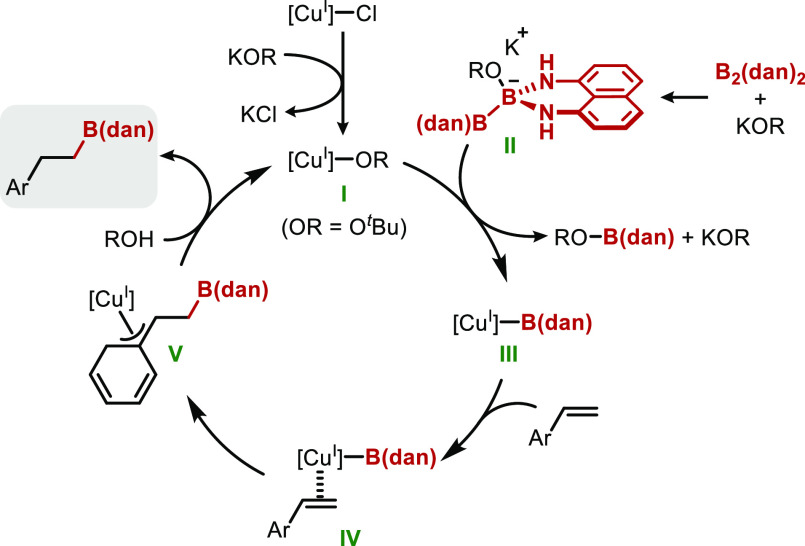
Proposed Catalytic Cycle

In conclusion, we developed a concise synthesis of B_2_(dan)_2_ from tetrahydroxydiboron. The usefulness of B_2_(dan)_2_ was demonstrated by using this reagent for the copper-catalyzed borylation of styrenes. Studies directed toward the use of B_2_(dan)_2_ for other borylation reactions as well as the application of alkyl-B(dan) for various transformations are ongoing.

## Data Availability

The data underlying this study are available in the published article and its online Supporting Information.

## Supporting Information Available

Experimental procedures and characterization data (PDF)
