# Supplementary material for: Copper-Catalyzed Borylation of Styrenes by 1,8-Diaminonaphthalene-Protected Diboronic Acid
Source: Org Lett. 2023 Mar 22;25(12):2093–7. doi: 10.1021/acs.orglett.3c00451 (PMC10071482; doi:10.1021/acs.orglett.3c00451)
Supplement: Supplementary file 1 — ol3c00451_si_001.pdf [file ol3c00451_si_001.pdf]

Supporting Information

Copper-Catalyzed Borylation of Styrenes by 1,8-Diaminonaphthalene-Protected Diboronic Acid

Taiga Yasuda, Yusuke Yoshigoe, Shinichi Saito*

Department of Chemistry, Faculty of Science, Tokyo University of Science, Kagurazaka, Shinjuku, Tokyo 162-8601, Japan

* E-mail: ssaito@rs.tus.ac.jp

Contents

1. **General Considerations** S2
2. **Sublimation of 1,8-Diaminonaphthalene (H_2-dan)** S3
3. **Preparation of B_2(dan)$_2$** S3
4. **Optimization of the Reaction Conditions** S4
5. **Detailed Studies on the Solvent Effect** S5
6. **General Procedure for the Cu-catalyzed Hydroboration Reaction** S6
7. **Characterization Data** S7
8. **Reactions of Alkyl-B(dan) 4a** S15
9. **Mechanistic Studies** S16
10. **References** S19
11. **NMR spectra** S20

1. General Considerations

Unless otherwise noted, reagents and solvents were commercially available and used without further purification. Styrene derivatives, **3e**, **3i**, **3l**, **3m**, **3n**, **3r** and **3s**, were synthesized by reported method.[1] An oil bath or a bead bath was used as the heat source, and the external temperature was reported. NMR spectra were recorded on a 400 MHz spectrometer. Chemical shifts were reported in delta units (δ) relative to $CHCl_3$ in $CDCl_3$ (7.24 ppm for ^{1}H NMR and 77.23 ppm for ^{13}C NMR). The $^{11}B\{^{1}H\}$ NMR spectra were measured in quartz NMR tube, and the chemical shifts are referenced to the $^{11}B\{^{1}H\}$ signal of $BF_3\cdot OEt_2$ (0.00 ppm) as an external standard. The ^{19}F NMR chemical shifts are referenced to the ^{19}F signal of C_6F_6 (-164.9 ppm) as an external standard. Multiplicity is indicated by s (singlet), d (doublet), t (triplet), m (multiplet), br (broad). Coupling constants, *J*, are reported in Hertz. IR spectra were recorded on a Fourier transform infrared spectrometer using a diamond ATR module. Thin layer chromatography was performed on Merck silica gel 60F-254 plates. Column chromatography was performed using Kanto Chemical silica gel 60N (spherical, neutral 40–50 μm). High-resolution mass spectra (HRMS) were obtained on a quadrupole time-of-flight (TOF) mass analyzer.

2. Sublimation of 1,8-Diaminonaphthalene (H_2-dan)

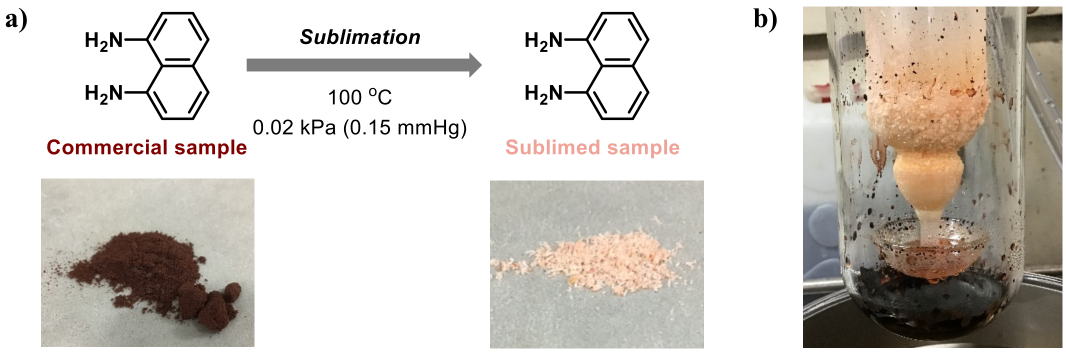

Figure S1. **a)** Sublimation of commercial H_2-dan. **b)** Sublimed H_2-dan in a sublimation apparatus.

1,8-Diaminonaphthalene (commercial sample, dark brown) was placed in a sublimation apparatus, and the sublimed sample (pink) was isolated by heating the apparatus at 100 °C in vacuo (0.15 mmHg, Figure S1).

3. Preparation of $B_2(dan)_2$

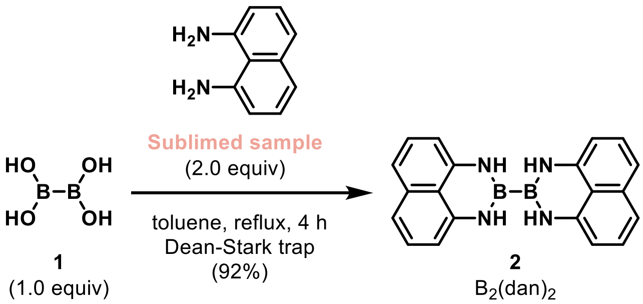

A mixture of $B_2(OH)_4$ (79 mmol, 7.1 g) and sublimed 1,8-diaminonaphthalene (159 mmol, 25 g, pink) in toluene (160 mL) was refluxed under Ar for 4 h with a Dean-Stark trap. The progress of reaction was monitored by TLC (hexane/AcOEt = 5:1). After the solvent was removed by evaporation, the residue was suspended in hot AcOEt (300 mL) and the insoluble material was removed by filtration. The filtrate was evaporated, and the residue was purified by recrystallization (AcOEt) to afford $B_2(dan)_2$ [2] as a white solid (92%, 79 mmol, 24 g). $B_2(dan)_2$ is air-stable and it could be purified by column chromathography (hexane/AcOEt = 5:1).

^{1}H NMR (DMSO-d_6, 400 MHz): δ 8.09 (s, 4H), 7.07 (t, 4H, J = 7.8 Hz), 6.87 (d, 4H, J = 8.2 Hz), 6.32 (d, 4H, J = 6.8 Hz); ($CDCl_3$, 400 MHz): δ 7.09 (t, 4H, J = 7.9 Hz), 7.01 (d, 4H, J = 8.0 Hz), 6.31 (d, 4H, J = 7.3 Hz), 5.96 (s, 4H).

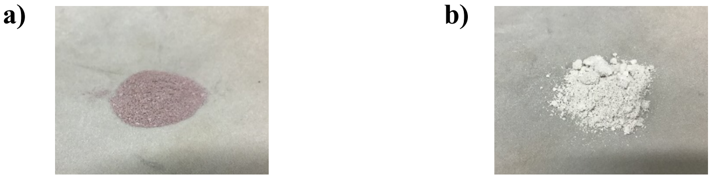

Figure S2. **a)** $B_2(dan)_2$ synthesized from commercial H_2-dan. **b)** $B_2(dan)_2$ synthesized from sublimed H_2-dan.

4. Optimization of the Reaction Conditions

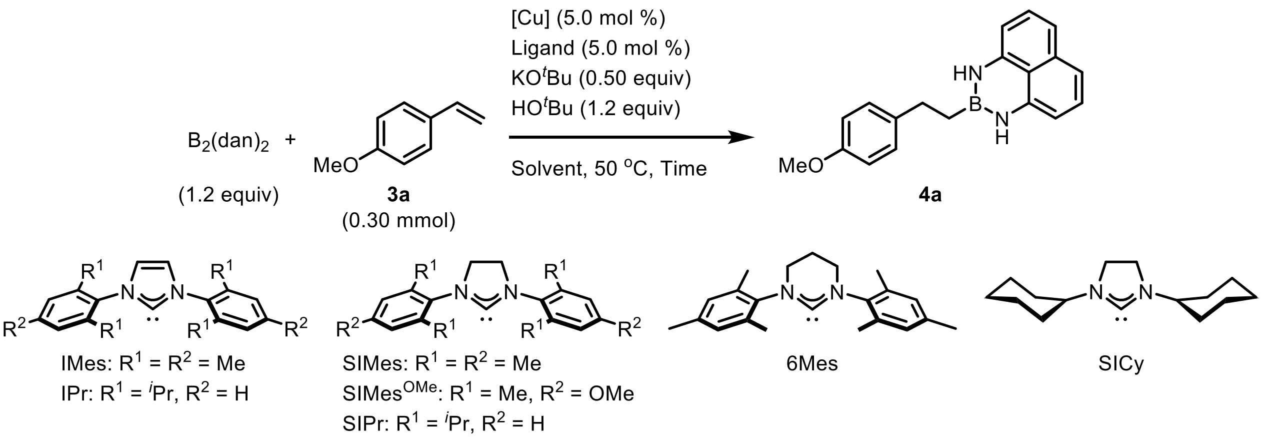

A mixture of copper complex (0.015 mmol, 5.0 mol %), ligand (0.015 mmol, 5.0 mol %, if any), $B_2(dan)_2$ (120 mg, 0.36 mmol, 1.2 equiv), and KO^{t}Bu (16.8 mg, 0.15 mmol, 0.50 equiv) in solvent (0.85 mL) was stirred at rt for 30 min. To the mixture was added alkene **3a** (0.040 mL, 0.30 mmol, 1.0 equiv) and HO^{t}Bu (0.034 mL, 0.36 mmol, 1.2 equiv), and the resulting mixture was stirred at 50 °C under Ar. The progress of the reaction was monitored by TLC. The reaction mixture was filtered through a pad of silica gel, and the pad was washed with AcOEt (10 mL X 3). The combined filtrate was evaporated, and the residue was purified by silica gel column chromatography (hexane/AcOEt) to afford **4a**.

Table S1. Optimization of the reaction conditions

Entry	[Cu]	Ligand	Solvent	Time (h)	Yield (%)
1	CuI	Xantphos	1,4-dioxane	4	88
2	CuBr	Xantphos	1,4-dioxane	2	91
3	$CuBr_2$	Xantphos	1,4-dioxane	2	96
4	$CuCl_2$	Xantphos	1,4-dioxane	2	92
5	$Cu(OAc)_2$	Xantphos	1,4-dioxane	2	72
6	$CuBr_2$	dppf	1,4-dioxane	2	70
7	$CuBr_2$	dppp	1,4-dioxane	2	55
8	$CuBr_2$	PPh_3 (10 mol %)	1,4-dioxane	2	0
9	$CuBr_2$	1, 10-phenanthroline	1,4-dioxane	2	0
10	CuCl(IMes)	-	1,4-dioxane	2	90
11	CuCl(SIMes)	-	1,4-dioxane	1	95
12	CuCl(SIMes^{OMe})	-	1,4-dioxane	2	16
13	CuBr(6Mes)	-	1,4-dioxane	2	44
14	CuCl(IPr)	-	1,4-dioxane	2	trace
15	CuCl(SIPr)	-	1,4-dioxane	2	trace
16	CuCl(SICy)	-	1,4-dioxane	2	trace
17	CuCl(SIMes)	-	THF	1	49
18	CuCl(SIMes)	-	toluene	1	97

5. Detailed Studies on the Solvent Effect

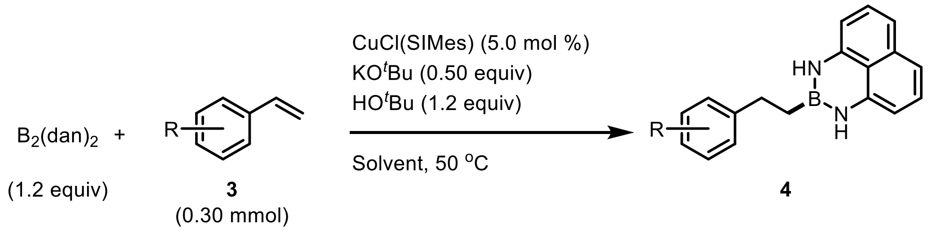

3	Reaction time and yield of **4**	
	in toluene	in 1,4-dioxane
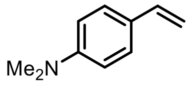 **3e**	1 h, 96%	22 h, 24%
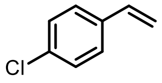 **3g**	3 h, 91%	22 h, 48%
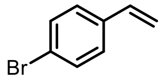 **3h**	3 h, 69%	24 h, 17%
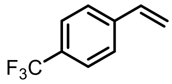 **3i**	2 h, 66%	20 h, 42%
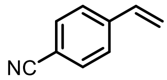 **3k**	24 h, 50%	20 h, trace
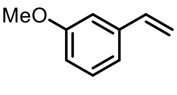 **3l**	1 h, 92%	7 h, 82%
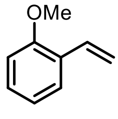 **3m**	1 h, 89%	21 h, 56%
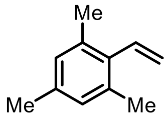 **3o**	3 h, 91%	26 h, 45%
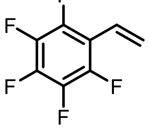 **3p**	2 h, 60%	24 h, 14%
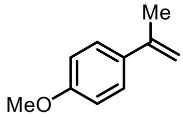 **3r**	24 h, 72%	17 h, 6%

6. General Procedure for the Cu-catalyzed Borylation Reaction

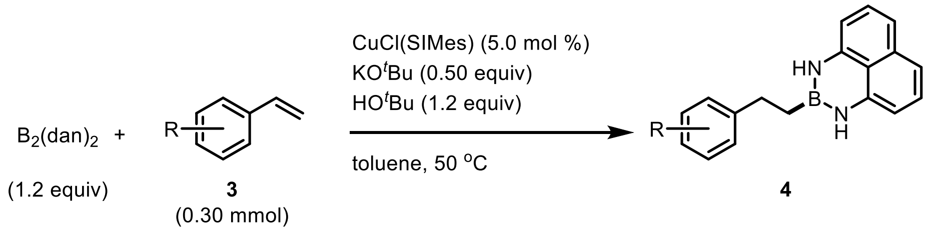

3

4

A mixture of CuCl(SIMes) (6.1 mg, 0.015 mmol, 5.0 mol %), $B_2(dan)_2$ (120 mg, 0.36 mmol, 1.2 equiv), and KO^{t}Bu (16.8 mg, 0.15 mmol, 0.50 equiv) in toluene (0.85 mL) was stirred at rt for 30 min. To the mixture was added alkene **3** (0.30 mmol, 1.0 equiv) and HO^{t}Bu (0.034 mL, 0.36 mmol, 1.2 equiv). The resulting mixture was stirred at 50 °C under Ar, and the progress of the reaction was monitored with TLC. The reaction mixture was filtered through a pad of silica gel, and the pad was washed with AcOEt (10 mL X 3). The combined filtrate was evaporated, and the residue was purified by silica gel column chromatography (hexane/AcOEt = 10:1) to afford **4**.

7. Characterization Data

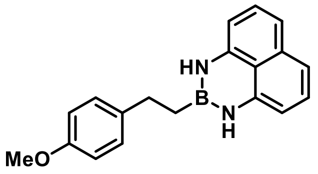

4a

2-(4-Methoxyphenethyl)-2,3-dihydro-1*H*-naphtho[1,8-*de*][1,3,2]diazaborinine (**4a**): **4a** (0.088 g, 0.29 mmol, 97%) was synthesized from 4-methoxystyrene (**3a**, 0.040 g, 0.30 mmol) as a white solid. Mp: 85.2–86.6 °C; ^{1}H NMR ($CDCl_3$, 400 MHz): δ 7.15 (d, 2H, *J* = 8.2 Hz), 7.07 (t, 2H, *J* = 7.8 Hz), 6.98 (d, 2H, *J* = 7.8 Hz), 6.84 (dd, 2H, *J* = 6.6, 2.1 Hz, 2H), 6.24 (dd, 2H, *J* = 7.3, 0.9 Hz), 5.54 (s, 2H), 3.78 (s, 3H), 2.72 (t, 2H, *J* = 8.2 Hz), 1.20 (t, 2H, *J* = 8.0 Hz); ^{13}C{^{1}H} NMR ($CDCl_3$, 100 MHz): δ 158.0, 141.3, 136.5, 136.1, 129.0, 127.7, 119.8, 117.6, 114.1, 105.7, 55.5, 30.1, the signal of the boron-bound carbon atom was obscure due to the quadrupolar boron nucleus; ^{11}B{^{1}H} NMR ($CDCl_3$, 128 MHz): δ 32.1; IR (ATR, cm^{-1}): 3390, 3054, 2914, 1602, 1416, 1244, 821; HRMS (ESI+) calcd. for $C_{19}H_{20}{}^{10}BN_2O$ ([M+H]$^+$): 302.1700. Found 302.1688.

When pink $B_2(dan)_2$ was used, **4a** (0.087 g, 0.29 mmol, 96%) was obtained as an off-white solid.

Large-scale synthesis of 4a: a mixture of CuCl(SIMes) (0.081 g, 0.20 mmol, 5.0 mol %), $B_2(dan)_2$ (1.20 g, 4.8 mmol, 1.2 equiv), and KO*t*Bu (0.224 g, 2.0 mmol, 0.50 equiv) in toluene (11 mL) was stirred at rt for 30 min. To the mixture was added alkene **3a** (0.537 g, 4.0 mmol, 1.0 equiv) and HO*t*Bu (0.46 mL, 4.8 mmol, 1.2 equiv). The resulting mixture was stirred at 50 °C under Ar, and the progress of the reaction was monitored with TLC. The reaction mixture was filtered through a pad of silica gel, and the pad was washed with AcOEt (20 mL X 3). The combined filtrate was evaporated, and the residue was purified by silica gel column chromatography (hexane/AcOEt = 10:1) to afford **4a** (1.1 g, 3.7 mmol, 92%) as a white solid.

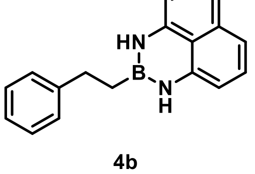

4b

2-Phenethyl-2,3-dihydro-1*H*-naphtho[1,8-*de*][1,3,2]diazaborinine (**4b**): **4b** (0.074 g, 0.27 mmol, 91%) was synthesized from styrene (**3b**, 0.031 g, 0.30 mmol) as a colorless oil. ^{1}H NMR ($CDCl_3$, 400 MHz): 7.34-7.30 (m, 2H), 7.27-7.22 (m, 3H), 7.10 (t, 2H, *J* = 7.8 Hz), 7.01 (dd, 2H, *J* = 8.2, 0.9 Hz), 6.25 (dd, 2H, *J* = 7.3, 0.9 Hz), 5.55 (s, 2H), 2.79 (t, 2H, *J* = 8.0 Hz), 1.24 (t, 2H, *J* = 8.0 Hz); ^{13}C{^{1}H} NMR ($CDCl_3$, 100 MHz): δ 144.0, 141.2, 136.5, 128.7, 128.2, 127.7, 126.1, 119.8, 117.6, 105.7, 31.0, 16.8 (br); ^{11}B{^{1}H} NMR ($CDCl_3$, 128 MHz): δ 32.4; NMR data matched those reported previously. [3]

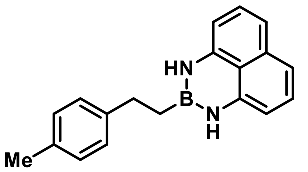

4c

2-(4-Methylphenethyl)-2,3-dihydro-1*H*-naphtho[1,8-*de*][1,3,2]diazaborinine (**4c**): **4c** (0.082 g, 0.29 mmol, 96%) was synthesized from 4-methylstyrene (**3c**, 0.036 g, 0.30 mmol) as a white solid. Mp: 93.7–94.7 °C; ^{1}H NMR ($CDCl_3$, 400 MHz): δ 7.13-7.04 (m, 6H), 6.97 (d, 2H, *J* = 7.8 Hz), 6.23 (dd, 2H, *J* = 7.3, 0.9 Hz), 5.55 (s, 2H), 2.74 (t, 2H, *J* = 8.2 Hz), 2.31 (s, 3H), 1.21 (t, 2H, *J* = 8.2 Hz); ^{13}C{^{1}H} NMR ($CDCl_3$, 100 MHz): δ 141.3, 141.0, 136.5, 135.5, 129.4, 128.0, 127.7, 119.8, 117.6, 105.7, 30.5, 21.2, 17.1 (br); ^{11}B{^{1}H} NMR ($CDCl_3$, 128 MHz): δ 32.6; the NMR data matched those reported previously. [4]

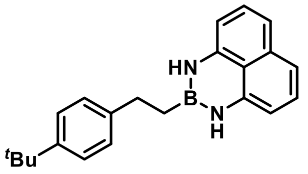

4d

2-(4-(*tert*-Butyl)phenethyl)-2,3-dihydro-1*H*-naphtho[1,8-*de*][1,3,2]diazaborinine (**4d**): **4d** (0.091 g, 0.28 mmol, 92%) was synthesized from 1-(*tert*-butyl)-4-vinylbenzene (**3d**, 0.048 g, 0.30 mmol) as a white solid. Mp: 104.7–105.8 °C; ^{1}H NMR ($CDCl_3$, 400 MHz): δ 7.32 (dd, 2H, *J* = 6.4, 1.8 Hz), 7.17 (d, 2H, *J* = 8.2 Hz), 7.07 (t, 2H, *J* = 7.8 Hz), 6.98 (d, 2H, *J*=8.2 Hz), 6.23 (dd, 2H, *J* = 7.3, 0.9 Hz), 5.54 (s, 2H), 2.75 (t, 2H, *J* = 8.2 Hz), 1.31 (s, 9H), 1.23 (t, 2H, *J* = 8.2 Hz); ^{13}C{^{1}H} NMR ($CDCl_3$, 100 MHz): δ 148.9, 141.3, 140.9, 136.5, 127.82, 127.75, 125.6, 119.8, 117.6, 105.7, 34.6, 31.6, 30.4, the signal of the boron-bound carbon atom was obscure due to the quadrupolar boron nucleus; ^{11}B{^{1}H} NMR ($CDCl_3$, 128 MHz): δ 32.3; IR (ATR, cm^{-1}): 3398, 2961, 1507, 1410, 818; HRMS (ESI+) calcd. for $C_{22}H_{25}{}^{10}BN_2$ ($[M]^+$): 327.2142. Found 327.2150.

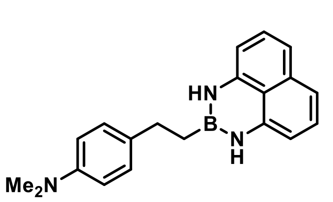

4e

4-(2-(1*H*-Naphtho[1,8-*de*][1,3,2]diazaborinin-2(3*H*)-yl)ethyl)-*N*,*N*-dimethylaniline (**4e**): **4e** (0.091 g, 0.29 mmol, 96%) was synthesized from 4-(*N*,*N*-dimethylamino)styrene (**3e**, 0.044 g, 0.30 mmol) as a white solid. Mp: 102.5–103.4 °C; ^{1}H NMR ($CDCl_3$, 400 MHz): δ 7.14-7.06 (m, 4H), 6.99 (d, 2H, *J* = 8.2 Hz), 6.72 (d, 2H, *J* = 8.7 Hz), 6.24 (d, 2H, *J* = 7.3 Hz), 5.56 (s, 2H), 2.92 (s, 6H), 2.70 (t, 2H, *J* = 8.0 Hz), 1.20 (t, 2H, *J* = 8.0 Hz); ^{13}C{^{1}H} NMR ($CDCl_3$, 100 MHz): δ 149.3, 141.3, 136.5, 132.2, 128.7, 127.7, 119.8, 117.5, 113.4, 105.6, 41.1, 29.9, 17.0 (br); ^{11}B{^{1}H} NMR

($CDCl_3$, 128 MHz): δ 32.8; IR (ATR, cm^{-1}): 3412, 3054, 2912, 1600, 1521, 822; HRMS (ESI+) calcd. for $C_{20}H_{23}{}^{10}BN_3$ ($[M+H]^+$): 315.2016. Found 315.2015.

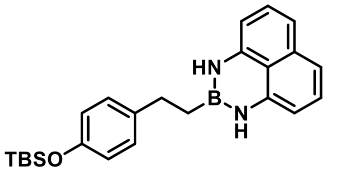

4f

2-(4-((*tert*-Butyldimethylsilyl)oxy)phenethyl)-2,3-dihydro-1*H*-naphtho[1,8-*de*][1,3,2]diazaborinine (**4f**): **4f** (0.111 g, 0.27 mmol, 92%) was synthesized from *tert*-butyldimethyl(4-vinylphenoxy)silane (**3f**, 0.070 g, 0.30 mmol) as a colorless oil. ^{1}H NMR ($CDCl_3$, 400 MHz): δ 7.10-7.05 (m, 4H), 6.99 (d, 2H, J = 7.8 Hz), 6.77 (dd, 2H, J = 6.4, 2.3 Hz), 6.23 (dd, 2H, J = 7.3, 0.9 Hz), 5.53 (s, 2H), 2.71 (t, 2H, J = 8.0 Hz), 1.19 (t, 2H, J = 8.0 Hz), 0.98 (t, 9H, J = 3.0 Hz, 9H), 0.19 (t, 6H, J = 3.2 Hz); $^{13}C\{^1H\}$ NMR ($CDCl_3$, 100 MHz): δ 153.9, 141.3, 136.6, 136.5, 129.0, 127.7, 120.2, 119.8, 117.6, 105.7, 30.2, 25.9, 18.4, 16.9 (br), -4.2; $^{11}B\{^1H\}$ NMR ($CDCl_3$, 128 MHz): δ 32.6; IR (ATR, cm^{-1}):3407, 3053, 2928, 2857, 1603, 1507, 1260, 916; HRMS (ESI+) calcd. for $C_{24}H_{31}{}^{10}BN_2OSi$ ($[M]^+$): 401.2330. Found 401.2336.

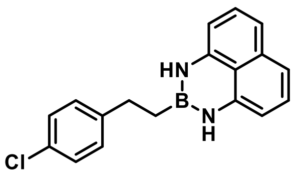

4g

2-(4-Chlorophenethyl)-2,3-dihydro-1*H*-naphtho[1,8-*de*][1,3,2]diazaborinine (**4g**): **4g** (0.084 g, 0.27 mmol, 91%) was synthesized from 4-chlorostyrene (**3g**, 0.042 g, 0.30 mmol) as a white solid. Mp: 128.7–130.0 °C; ^{1}H NMR ($CDCl_3$, 400 MHz): δ 7.25 (d, 2H, J = 8.2 Hz), 7.15 (d, 2H, J = 8.2 Hz), 7.07 (t, 2H, J = 7.8 Hz), 6.99 (d, 2H, J = 8.2 Hz), 6.25 (dd, 2H, J = 7.3, 0.9 Hz), 5.55 (s, 2H), 2.74 (t, 2H, J = 8.2 Hz), 1.19 (t, 2H, J = 8.2 Hz); $^{13}C\{^1H\}$ NMR ($CDCl_3$, 100 MHz): δ 142.5, 141.1, 136.5, 131.7, 129.5, 128.8, 127.8, 119.8, 117.8, 105.8, 30.4, the signal of the boron-bound carbon atom was obscure due to the quadrupolar boron nucleus; $^{11}B\{^1H\}$ NMR ($CDCl_3$, 128 MHz): δ 32.6; IR (ATR, cm^{-1}): 3414, 3052, 2930, 1603, 1508, 1413, 820; HRMS (ESI+) calcd. for $C_{18}H_{16}{}^{10}B^{35}ClN_2$ ($[M]^+$): 305.1126. Found 305.1132.

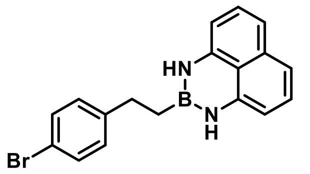

4h

2-(4-Bromophenethyl)-2,3-dihydro-1*H*-naphtho[1,8-*de*][1,3,2]diazaborinine (**4h**): **4h** (0.072 g, 0.21 mmol, 69%) was synthesized from 4-bromostyrene (**3g**, 0.055 g, 0.30 mmol) as a white solid. Mp: 142.6–143.9 °C; ^{1}H NMR ($CDCl_3$, 400 MHz): δ 7.40 (d, 2H, *J* = 8.2 Hz), 7.11-7.05 (m, 4H), 6.99 (d, 2H, *J* = 8.2 Hz), 6.25 (d, 2H, *J* = 7.3 Hz), 5.55 (s, 2H), 2.72 (t, 2H, *J* = 8.2 Hz), 1.19 (t, 2H, *J* = 8.2 Hz); ^{13}C{^{1}H} NMR ($CDCl_3$, 100 MHz): δ 143.0, 141.1, 136.5, 131.7, 129.9, 127.8, 119.79, 119.75, 117.8, 105.8, 30.5, the signal of the boron-bound carbon atom was obscure due to the quadrupolar boron nucleus; ^{11}B{^{1}H} NMR ($CDCl_3$, 128 MHz): δ 31.9; IR (ATR, cm^{-1}): 3392, 2931, 1597, 1507, 1412, 819; HRMS (ESI+) calcd. for $C_{18}H_{16}{}^{10}B{}^{79}BrN_2$ ([M]$^+$): 349.0621. Found: 349.0634.

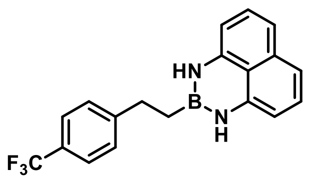

4i

2-(4-(Trifluoromethyl)phenethyl)-2,3-dihydro-1*H*-naphtho[1,8-*de*][1,3,2]diazaborinine (**4i**): **4i** (0.067 g, 0.20 mmol, 66%) was synthesized from 4-(trifluoromethyl)styrene (**3i**, 0.052 g, 0.30 mmol) as a white solid. Mp: 86.2–87.1 °C; ^{1}H NMR ($CDCl_3$, 400 MHz): δ 7.54 (d, 2H, *J* = 8.2 Hz), 7.33 (d, 2H, *J* = 8.2 Hz), 7.08 (t, 2H, *J* = 7.8 Hz), 7.00 (d, 2H, *J* = 7.8 Hz), 6.26 (d, 2H, *J* = 7.3 Hz), 5.56 (s, 2H), 2.82 (t, 2H, *J* = 8.4 Hz), 1.23 (t, 2H, *J* = 8.2 Hz); ^{13}C{^{1}H} NMR ($CDCl_3$, 100 MHz): δ 148.2, 141.0, 136.4, 128.4, 128.3 (q, $J_{F,C}$ = 32.0 Hz), 127.8, 125.5 (q, $J_{F,C}$ = 3.6 Hz), 124.6 (q, $J_{F,C}$ = 271.7 Hz), 119.8, 117.8, 105.8, 30.8, 16.6 (br); ^{11}B{^{1}H} NMR ($CDCl_3$, 128 MHz): δ 32.1; ^{19}F NMR ($CDCl_3$, 375 MHz): δ -65.4; IR (ATR, cm^{-1}): 3395, 3054, 2933, 2220, 1599, 1326, 819; HRMS (ESI+) calcd. for $C_{19}H_{16}{}^{10}BF_3N_2$ ([M]$^+$): 339.1389. Found: 339.1392.

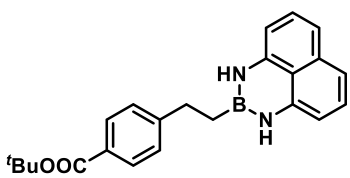

4j

***tert*-Butyl 4-(2-(1*H*-naphtho[1,8-*de*][1,3,2]diazaborinin-2(3*H*)-yl)ethyl)benzoate** (**4j**): **4j** (0.085 g, 0.23 mmol, 75%) was synthesized from *tert*-butyl 4-vinylbenzoate (**3j**, 0.061 g, 0.30 mmol) as a white solid. Mp: 195.1–196.0 °C; ^{1}H NMR ($CDCl_3$, 400 MHz): δ 7.90 (d, 2H, *J* = 7.8 Hz), 7.26 (d, 2H, *J* = 8.2 Hz), 7.07 (t, 2H, *J* = 7.8 Hz), 6.99 (d, 2H, *J* = 7.8 Hz), 6.25 (d, 2H, *J* = 7.3 Hz), 5.56 (s, 2H), 2.81 (t, 2H, *J* = 8.2 Hz), 1.57 (s, 9H), 1.22 (t, 2H, *J* = 8.2 Hz); ^{13}C{^{1}H} NMR ($CDCl_3$, 100 MHz): δ 166.0, 149.0, 141.1, 136.5, 130.0, 129.9, 128.0, 127.7, 119.8, 117.8, 105.8, 81.0,

31.1, 28.4, 16.7 (br); $^{11}B\{^1H\}$ NMR ($CDCl_3$, 128 MHz): δ 32.7; IR (ATR, cm^{-1}): 3427, 3370, 3055, 2976, 1691, 1602, 1297, 766; HRMS (ESI+) calcd. for $C_{23}H_{26}{}^{10}BN_2O_2$ ($[M+H]^+$): 372.2118. Found: 372.2126.

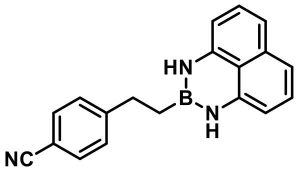

4k

4-(2-(*1H*-Naphtho[1,8-*de*][1,3,2]diazaborinin-2(3*H*)-yl)ethyl)benzonitrile (**4k**): **4k** (0.059 g, 0.20 mmol, 50%) was synthesized from 4-vinylbenzonitrile (**3i**, 0.039 g, 0.30 mmol) as a white solid. Mp: 204.1–205.3 °C; ^1H NMR ($CDCl_3$, 400 MHz):δ 7.57 (d, 2H, J = 8.2 Hz), 7.32 (d, 2H, J = 7.8 Hz), 7.08 (t, 2H, J = 7.8 Hz), 7.00 (d, 2H, J = 8.2 Hz), 6.27 (d, 2H, J = 7.3 Hz), 5.55 (s, 2H), 2.82 (t, 2H, J = 8.4 Hz), 1.22 (t, 2H, J = 8.2 Hz); $^{13}C\{^1H\}$ NMR ($CDCl_3$, 100 MHz): δ 149.7, 140.9, 136.5, 132.5, 128.9, 127.8, 119.8, 119.3, 117.9, 109.9, 105.9, 31.3, 16.5 (br); $^{11}B\{^1H\}$ NMR ($CDCl_3$, 128 MHz): δ 31.9; IR (ATR, cm^{-1}): 3394, 3055, 2919, 2220, 1604, 1514, 1415, 819; HRMS (ESI+) calcd. for $C_{19}H_{17}{}^{10}BN_3$ ($[M+H]^+$): 297.1546. Found: 297.1540.

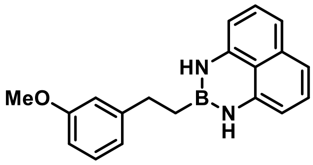

4l

2-(3-Methoxyphenethyl)-2,3-dihydro-1*H*-naphtho[1,8-*de*][1,3,2]diazaborinine (**4l**): **4l** (0.084 g, 0.27 mmol, 92%) was synthesized from 1-methoxy-3-vinylbenzene (**3l**, 0.040 g, 0.30 mmol) as a white solid. Mp: 90.2–91.3 °C; ^1H NMR ($CDCl_3$, 400 MHz): ^1H NMR ($CDCl_3$, 400 MHz) δ 7.19-7.15 (m, 2H), 7.06 (t, 2H, J = 8.0 Hz), 6.98-6.96 (m, 2H), 6.90 (td, 1H, J = 7.4, 1.1 Hz), 6.85 (d, 1H, J = 8.2 Hz), 6.24 (dd, 2H, J = 7.3, 0.9 Hz), 5.69 (s, 2H), 3.84 (s, 3H), 2.77 (t, 2H, J = 8.0 Hz), 1.20 (t, 2H, J = 7.8 Hz); $^{13}C\{^1H\}$ NMR ($CDCl_3$, 100 MHz): δ 157.2, 141.5, 136.5, 132.4, 129.5, 127.7, 127.2, 120.9, 119.8, 117.4, 110.5, 105.6, 55.5, 24.6, the signal of the boron-bound carbon atom was obscure due to the quadrupolar boron nucleus; $^{11}B\{^1H\}$ NMR ($CDCl_3$, 128 MHz): δ 32.5; IR (ATR, cm^{-1}): 3393, 3051, 2939, 1599, 1508, 1412, 762; HRMS (ESI+) calcd. for $C_{19}H_{20}{}^{10}BN_2O$ ($[M+H]^+$): 302.1700. Found: 302.1692.

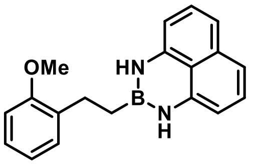

4m

2-(2-Methoxyphenethyl)-2,3-dihydro-1*H*-naphtho[1,8-*de*][1,3,2]diazaborinine (**4m**): **4m** (0.081 g, 0.27 mmol, 89%) was synthesized from 1-methoxy-2-vinylbenzene (**3m**, 0.040 g, 0.30 mmol) as a white solid. Mp: 82.6–83.3 °C;

^{1}H NMR ($CDCl_3$, 400 MHz): δ 7.21 (t, 1H, *J* = 8.0 Hz), 7.06 (t, 2H, *J* = 7.8 Hz), 6.99-6.97 (m, 2H), 6.84-6.72 (m, 3H), 6.24 (dd, 2H, *J* = 7.3, 0.9 Hz), 5.56 (s, 2H), 3.78 (s, 3H), 2.75 (t, 2H, *J* = 8.2 Hz), 1.22 (t, 2H, *J* = 8.2 Hz); $^{13}C\{^1H\}$ NMR ($CDCl_3$, 100 MHz): δ 160.0, 145.7, 141.2, 136.5, 129.6, 127.7, 120.6, 119.8, 117.6, 114.0, 111.3, 105.7, 55.4, 31.0, the signal of the boron-bound carbon atom was obscure due to the quadrupolar boron nucleus; $^{11}B\{^1H\}$ NMR ($CDCl_3$, 128 MHz): δ 32.2; IR (ATR, cm^{-1}): 3404, 3006, 2911, 1601, 1412, 766; HRMS (ESI+) calcd. for $C_{19}H_{19}{}^{10}BN_2O$ ([M^+]): 301.1621. Found: 301.1630.

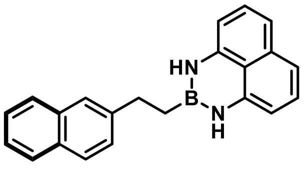

4n

2-(2-(Naphthalen-2-yl)ethyl)-2,3-dihydro-1*H*-naphtho[1,8-*de*][1,3,2]diazaborinine (**4n**): **4n** (0.093 g, 0.28 mmol, 93%) was synthesized from 2-vinylnaphthalene (**3n**, 0.046 g, 0.30 mmol) as a white solid. Mp: 114.2–115.6 °C; ^{1}H NMR ($CDCl_3$, 400 MHz): δ 7.81-7.76 (m, 3H), 7.66 (s, 1H), 7.46-7.37 (m, 3H), 7.06 (t, 2H, *J* = 7.8 Hz), 6.99-6.97 (m, 2H), 6.23 (dd, 2H, *J* = 7.3, 0.9 Hz), 5.60 (s, 2H), 2.94 (t, 2H, *J* = 8.2 Hz), 1.32 (t, 2H, *J* = 8.2 Hz); $^{13}C\{^1H\}$ NMR ($CDCl_3$, 100 MHz): δ 141.6, 141.2, 136.5, 133.9, 132.2, 128.3, 127.84, 127.75, 127.7, 127.2, 126.2, 125.9, 125.4, 119.8, 117.7, 105.7, 31.2, the signal of the boron-bound carbon atom was obscure due to the quadrupolar boron nucleus; $^{11}B\{^1H\}$ NMR ($CDCl_3$, 128 MHz): δ 32.3; IR (ATR, cm^{-1}): 3412, 3051, 2932, 1599, 1416, 759; HRMS (ESI+) calcd. for $C_{22}H_{20}{}^{10}BN_2$ ([M+H]$^+$): 322.1750. Found: 322.1741.

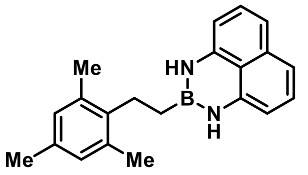

4o

2-(2,4,6-Trimethylphenethyl)-2,3-dihydro-1*H*-naphtho[1,8-*de*][1,3,2]diazaborinine (**4o**): **4o** (0.085 g, 0.27 mmol, 91%) was synthesized from 1,3,5-trimethyl-2-vinylbenzene (**3o**, 0.044 g, 0.30 mmol) as a white solid. Mp: 143.6–144.8 °C; ^{1}H NMR ($CDCl_3$, 400 MHz): δ 7.08 (t, 2H, *J* = 7.8 Hz), 6.99 (d, 2H, *J* = 8.2 Hz), 6.84 (s, 2H), 6.27 (d, 2H, *J* = 7.3 Hz), 5.55 (s, 2H), 2.70 (t, 2H, *J* = 8.7 Hz), 2.30 (s, 6H), 2.25 (s, 3H), 1.04 (t, 2H, *J* = 8.4 Hz); $^{13}C\{^1H\}$ NMR ($CDCl_3$, 100 MHz): δ 141.2, 137.9, 136.5, 135.7, 135.2, 129.2, 127.8, 119.8, 117.7, 105.7, 24.3, 21.0, 19.9, the signal of the boron-bound carbon atom was obscure due to the quadrupolar boron nucleus; $^{11}B\{^1H\}$ NMR ($CDCl_3$, 128 MHz): δ 33.2; IR (ATR, cm^{-1}): 3407, 2939, 1604, 1508, 1412, 764; HRMS (ESI+) calcd. for $C_{21}H_{24}{}^{10}BN_2$ ([M+H]$^+$): 314.2063. Found: 314.2063.

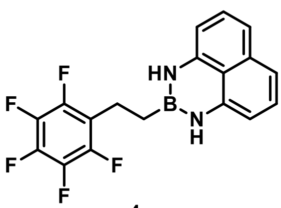

4p

2-(2-(Perfluorophenyl)ethyl)-2,3-dihydro-1*H*-naphtho[1,8-*de*][1,3,2]diazaborinine (**4p**): **4p** (0.065 g, 0.18 mmol, 60%) was synthesized from 1,2,3,4,5-pentafluoro-6-vinylbenzene (**3p**, 0.058 g, 0.30 mmol) as a white solid. Mp: 192.5–193.4 °C; ^{1}H NMR ($CDCl_3$, 400 MHz): δ 7.09 (t, 2H, J = 7.8 Hz), 7.01 (dd, 2H, J = 8.2, 0.9 Hz), 6.31 (dd, 2H, J = 7.3, 0.9 Hz), 5.63 (s, 2H), 2.81 (t, 2H, J = 8.4 Hz), 1.19 (t, 2H, J = 8.4 Hz); ^{13}C{^{1}H} NMR ($CDCl_3$, 100 MHz): δ 140.9, 136.5, 127.8, 119.8, 118.0, 106.0, 18.0, the signal of the fluorine-bound carbon atom was obscure due to the multiplicity by fluorine nucleus; ^{11}B{^{1}H} NMR ($CDCl_3$, 128 MHz): δ 31.7; ^{19}F NMR (375 MHz, $CDCl_3$): δ -148.1 (d, $J_{F,F}$ = 23.8 Hz), -161.0 (d, $J_{F,F}$ = 35.8 Hz), -165.7 (t, $J_{F,F}$ = 23.8 Hz); IR (ATR, cm^{-1}): 3438, 1599, 1498, 986, 764; HRMS (ESI+) calcd. for $C_{18}H_{12}{}^{10}BF_5N_2$ ([M]$^+$): 361.1045. Found: 361.1060.

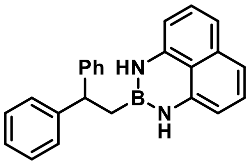

4q

2-(2,2-Diphenylethyl)-2,3-dihydro-1*H*-naphtho[1,8-*de*][1,3,2]diazaborinine (**4q**): **4q** (0.101 g, 0.29 mmol, 97%) was synthesized from 1,1-diphenylethene (**3q**, 0.054 g, 0.30 mmol) as a white solid. Mp: 169.9–171.3 °C; ^{1}H NMR ($CDCl_3$, 400 MHz): δ 7.30-7.26 (m, 8H), 7.20-7.17 (m, 2H), 7.01 (t, 2H, J = 7.8 Hz), 6.95-6.93 (m, 2H), 6.08 (dd, 2H, J = 7.3, 0.9 Hz), 5.31 (s, 2H), 4.19 (t, 1H, J = 8.2 Hz), 1.68 (d, 2H, J = 8.2 Hz); ^{13}C{^{1}H} NMR ($CDCl_3$, 100 MHz): δ 146.5, 141.1, 136.4, 128.8, 127.8, 127.7, 126.5, 119.7, 117.6, 105.7, 47.6, 22.9 (br); ^{11}B{^{1}H} NMR ($CDCl_3$, 128 MHz): δ 32.0; IR (ATR, cm^{-1}): 3400, 3056, 1600, 1507, 1414, 761; HRMS (ESI+) calcd. for $C_{24}H_{22}{}^{10}BN_2$ ([M+H]$^+$): 348.1907. Found: 348.1909.

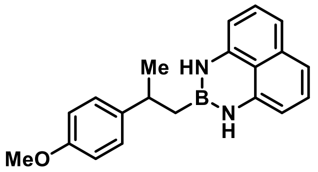

4r

2-(2-(4-Methoxyphenyl)propyl)-2,3-dihydro-1*H*-naphtho[1,8-*de*][1,3,2]diazaborinine (**4r**): **4r** (0.068 g, 0.21 mmol, 72%) was synthesized from 1-methoxy-4-(prop-1-en-2-yl)benzene (**3r**, 0.044 g, 0.30 mmol) as a colorless oil. ^{1}H NMR ($CDCl_3$, 400 MHz): δ 7.19 (dd, 2H, J = 6.6, 2.1 Hz), 7.06 (t, 2H, J = 7.8 Hz), 6.97 (d, 2H, J = 7.8 Hz), 6.87 (dd, 2H, J = 6.6, 2.1 Hz), 6.17 (d, 2H, J = 7.8 Hz), 5.41 (s, 2H), 3.80 (s, 3H), 2.94 (d, 1H, J = 8.2 Hz), 1.30 (d, 3H, J

= 7.3 Hz), 1.27-1.14 (m, 2H); $^{13}C\{^1H\}$ NMR ($CDCl_3$, 100 MHz): δ 158.1, 141.3, 140.9, 136.4, 127.69, 127.68, 119.7, 117.5, 114.1, 105.6, 55.5, 35.9, 25.8, the signal of the boron-bound carbon atom was obscure due to the quadrupolar boron nucleus; $^{11}B\{^1H\}$ NMR ($CDCl_3$, 128 MHz): δ 31.9; IR (ATR, cm^{-1}): 3413, 3052, 2954, 1603, 1511, 1247, 766; HRMS (ESI+) calcd. for $C_{20}H_{21}{}^{10}BN_2O$ ($[M^+]$): 315.1778. Found: 315.1783.

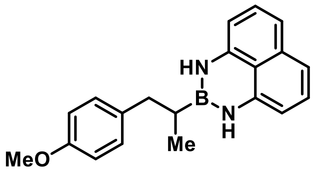

4s

2-(1-(4-Methoxyphenyl)propan-2-yl)-2,3-dihydro-1*H*-naphtho[1,8-*de*][1,3,2]diazaborinine (**4s**): **4s** (0.030 g, 0.96 mmol, 32%) was synthesized from (*E*)-1-methoxy-4-(prop-1-en-1-yl)benzene ((*E*)-**3s**, 0.044 g, 0.30 mmol) as a colorless oil. When (*Z*)-1-methoxy-4-(prop-1-en-1-yl)benzene ((*Z*)-**3s**, 0.044 g, 0.30 mmol) was used as a substrate, trace amount of **4s** was detected in the ^1H NMR spectrum of the crude mixture. ^1H NMR ($CDCl_3$, 400 MHz): δ 7.13-7.05 (m, 4H), 6.99 (dd, 2H, J = 8.2, 3.2 Hz), 6.83-6.81 (m, 2H), 6.24 (dd, 2H, J = 6.8, 2.7 Hz), 5.49 (s, 2H), 3.77 (s, 3H), 2.45-2.86 (m, 2H), 1.23-1.45 (m, 1H), 0.92-1.12 (d, 3H, J = 7.3 Hz); $^{13}C\{^1H\}$ NMR ($CDCl_3$, 100 MHz): δ 158.0, 141.3, 136.5, 134.1, 129.9, 127.8, 119.8, 117.6, 113.9, 105.8, 55.5, 39.5, 22.2 (br), 16.4; $^{11}B\{^1H\}$ NMR ($CDCl_3$, 128 MHz): δ 33.0; IR (ATR, cm^{-1}): 3406, 3051, 2950, 1603, 1510, 1246, 767; HRMS (ESI+) calcd. for $C_{20}H_{21}{}^{10}BN_2O$ ($[M^+]$): 315.1778. Found: 315.1787.

8. Reactions of Alkyl-B(dan) 4a

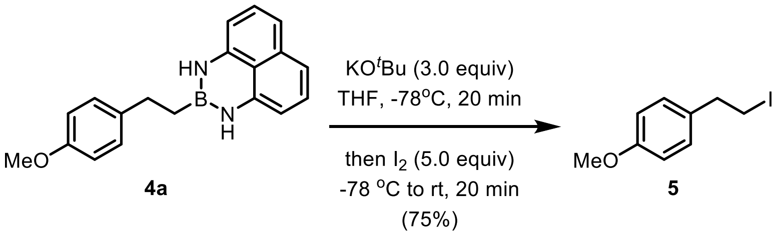

To a solution of **4a** (91 mg, 0.30 mmol) in THF (3 mL), KOtBu/THF (1.0 M, 0.90 mL, 0.90 mmol, 3.0 equiv) was added dropwise at -78 °C under Ar. After the mixture was stirred for 20 min, I_2 (381 mg, 1.5 mmol, 5.0 equiv) was added to the mixture then warmed to rt. After 20 min, sat. $Na_2S_2O_3$ aq. (10 mL) was added to the reaction mixture, and aqueous phase was washed with hexane (20 mL). The organic layer was washed with water (10 mL) and dried with Na_2SO_4. After the solvent was evaporated, the crude mixture was purified by silica gel column chromatography (hexane/AcOEt = 20:1) to afford **5** (59 mg, 0.23 mmol, 75%). [5] The NMR data matched those reported previously. [6]

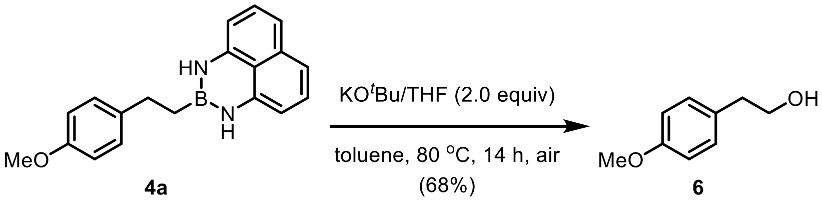

To a mixture of **4a** (38 mg, 0.13 mmol) in toluene (3 mL), KOtBu/THF (1.0 M, 0.25 mL, 0.25 mmol, 2.0 equiv) was added at rt under air. The mixture was stirred for 14 h at 80 °C. The reaction mixture was filtered through a pad of silica gel, and the pad was washed with AcOEt (10 mL X 3). The combined filtrate was evaporated, and the residue was purified by silica gel column chromatography (hexane/AcOEt = 5:1) to afford **6** (13 mg, 0.085 mmol, 68%). The NMR data matched those reported previously. [7]

9. Mechanistic Studies

Borate formation of $B_2(dan)_2$ with KO*t*Bu

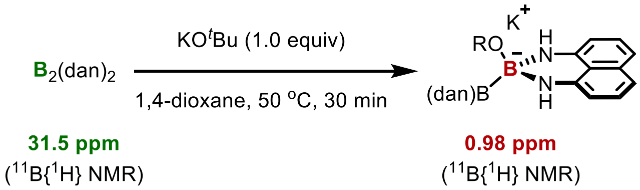

A mixture of $B_2(dan)_2$ (33 mg, 0.10 mmol) and KO*t*Bu (11 mg, 0.10 mmol, 1.0 equiv) in 1,4-dioxane (1.0 mL) was stirred at 50 °C for 30 min under Ar. An aliquot (0.5 mL) was transferred into a quartz NMR tube under Ar, and $^{11}B\{^1H\}$ NMR spectrum was measured at rt (Figure S3). The appearance of a signal at 0.98 ppm indicated that a borate species was formed by the reaction of $B_2(dan)_2$ with KO*t*Bu.

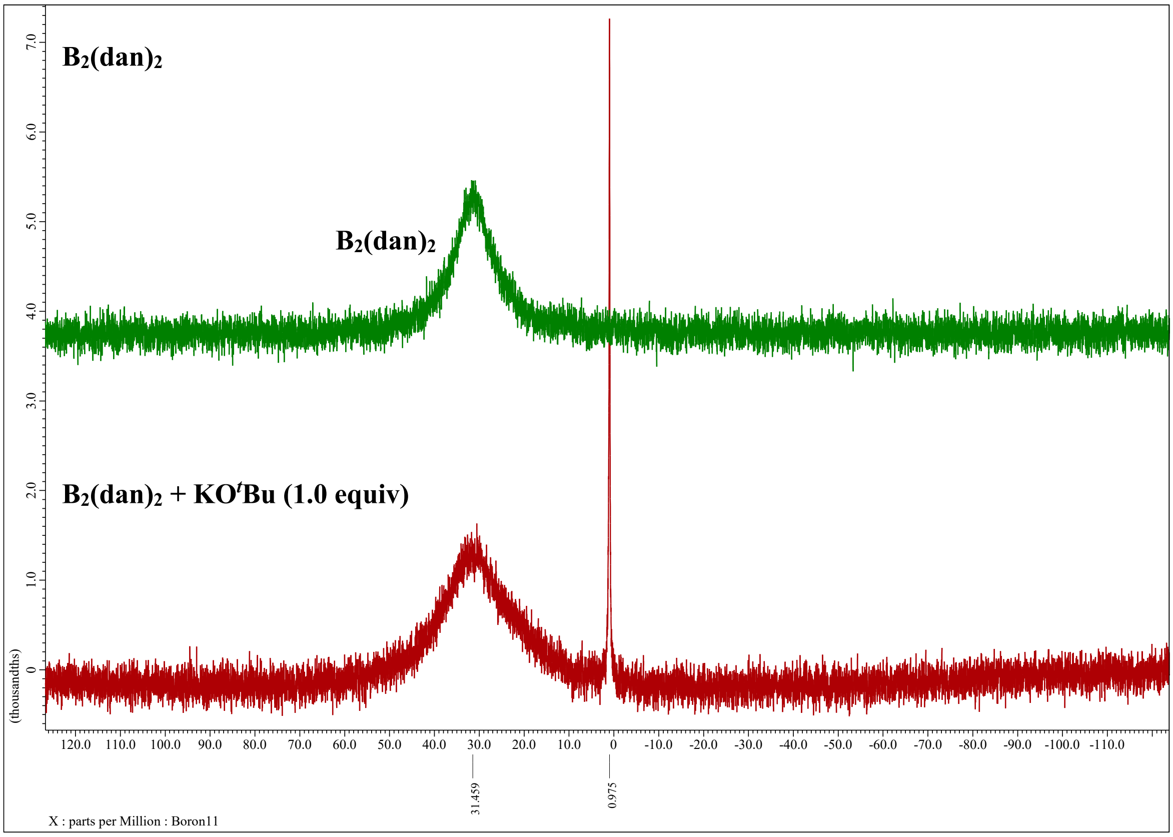

Figure S3. $^{11}B\{^1H\}$ NMR (128 MHz, 1,4-dioxane) spectra of $B_2(dan)_2$ and the mixture of $B_2(dan)_2$ and KO*t*Bu.

Synthesis of $B_2(dan)_2$-d_4

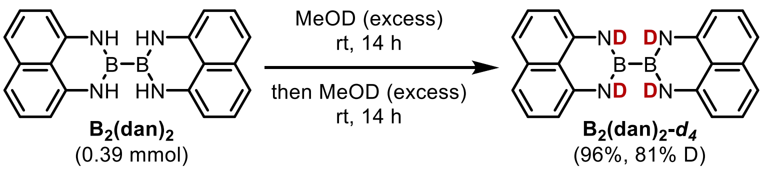

A solution of $B_2(dan)_2$ (130 mg, 0.39 mmol) in MeOD (99% D, 3 mL) was stirred at rt for 14 h under Ar. The solvent was evaporated, and the solid was dissolved again in MeOD (99% D, 3 mL). The solution was stirred at rt for 14 h under Ar, and the solvent was removed to obtain $B_2(dan)_2$-d_4 (96%, 81% D) as an off-white solid. The degree of deuterium incorporation of $B_2(dan)_2$-d_4 was found to be 81% at the N–H position by ^{1}H NMR analysis (Figure S4).

In order to reduce the deuteration loss due to water and possible residual acidic impurities in the solvent used for NMR analysis, it was imperative to include a simple pre-treatment for the solvent. $CDCl_3$ was thoroughly washed with equal volume of D_2O followed by drying over sodium sulfate before use.[8]

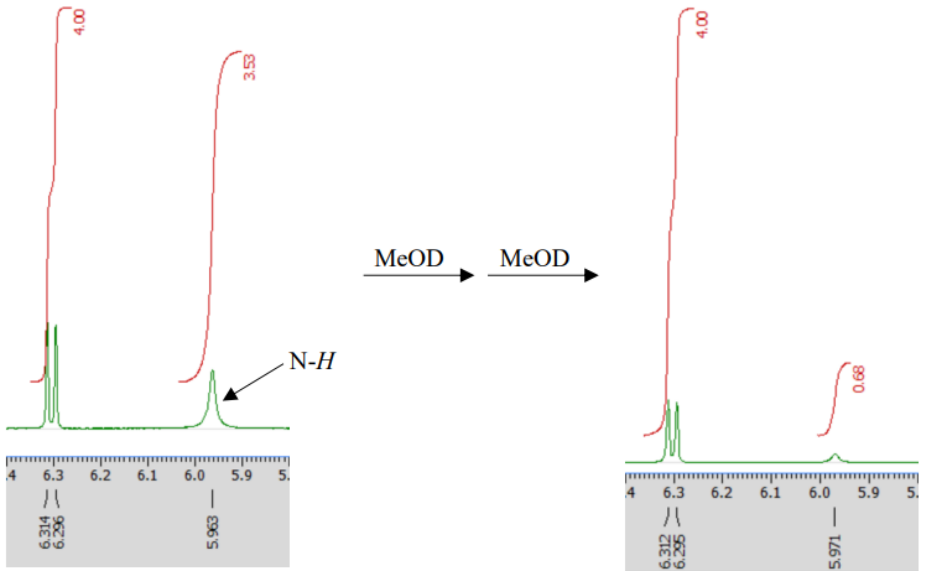

Figure S4. Deuteration of N–H moiety of $B_2(dan)_2$.

Reaction of deuterated $B_2(dan)_2$ and MeOD

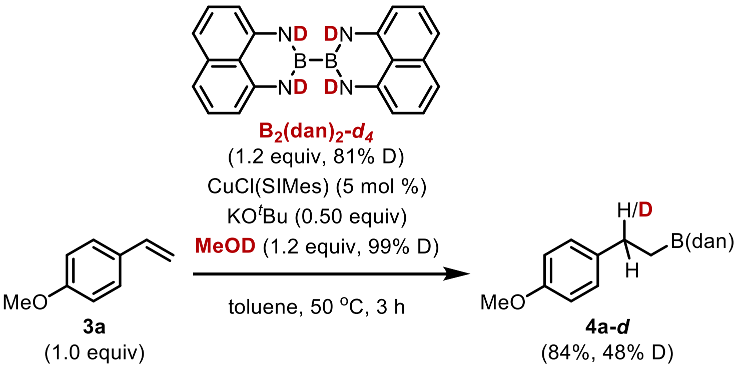

A mixture of CuCl(SIMes) (2.0 mg, 5.0 µmol, 5.0 mol %), $B_2(dan)_2$-d_4 (40.6 mg, 0.12 mmol, 1.2 equiv), and KO^{t}Bu (5.6 mg, 0.050 mmol, 0.50 equiv) in toluene (0.28 mL) was stirred at rt for 30 min. To the mixture was added alkene **3a** (13.4 mg, 0.10 mmol, 1.0 equiv) and MeOD (5.0 µL, 0.12 mmol, 1.2 equiv). The resulting mixture was stirred at 50 °C under Ar, and the progress of the reaction was monitored with TLC. The reaction mixture was filtered through a pad of silica gel, and the pad was washed with AcOEt (10 mL X 3). The combined filtrate was evaporated, and the residue was purified by silica gel column chromatography (hexane/AcOEt = 10:1) to afford **4a-*d*** (25 mg, 84 mmol, 84%). The degree of deuterium incorporation of **4a-*d*** was found to be 48% at the benzylic position by ^{1}H NMR analysis (Figure S5).

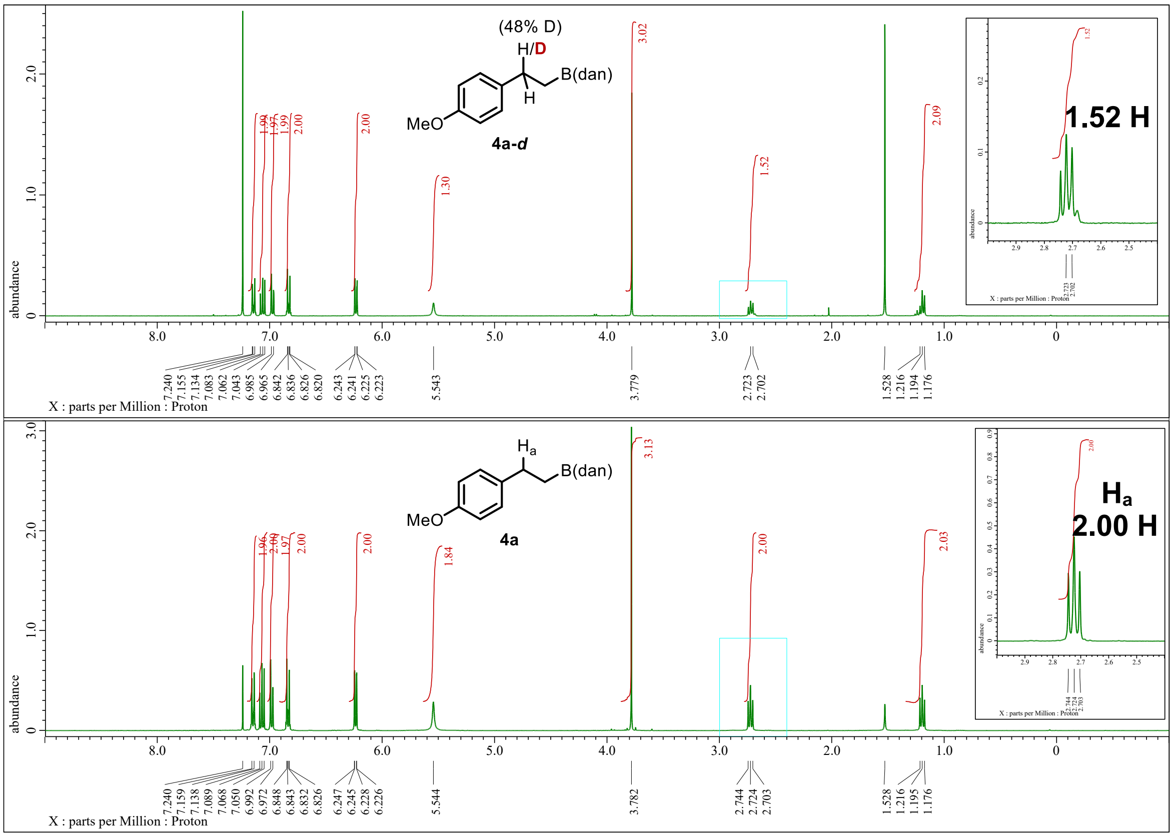

Figure S5. ^{1}H NMR (400 MHz, $CDCl_3$) spectrum of **4a-*d***.

10. References

1 Huang, J.; Hu, G.; An, S.; Chen, D.; Li, M.; Li, P. Synthesis of *N*-Alkylpyridin-4-ones and Thiazolo[3,2-*a*]pyridin-5-ones through Pummerer-Type Reactions. *J. Org. Chem.* **2019**, *84*, 9758−9769.

2 Xie, X.; Haddow, M. F.; Mansell, S. M.; Norman, N. C.; Russell, C. A. New polycyclic borazine species. *Chem. Commun.* **2011**, *47*, 3748–3750.

3 Zhong, M.; Gagné, Y.; Hope, T. O.; Pannecoucke, X.; Frenette, M.; Jubault, P.; Poisson, T. Copper-Photocatalyzed Hydroboration of Alkynes and Alkenes. *Angew. Chem. Int. Ed.* **2021**, *60*, 14498–14503.

4 Miralles, N.; Cid, J.; Cuenca, A. B.; Carbó J. J.; Fernández, E. Mixed diboration of alkenes in a metal-free context. *Chem. Commun.* **2015**, *51*, 1693–1696.

5 Larsen, M. A.; Wilson, C. V.; Hartwig, J. F. Iridium-Catalyzed Borylation of Primary Benzylic C–H Bonds without a Directing Group: Scope, Mechanism, and Origins of Selectivity. *J. Am. Chem. Soc.* **2015**, *137*, 8633–8643.

6 Zhao, M.; Barrado, A. G.; Sprenger, K.; Golz, C.; Mata, R. A.; Alcarazo, M. Electrophilic Cyanative Alkenylation of Arenes. *Org. Lett.* **2020**, *22*, 4932–4937.

7 Szostak, M.; Spain, M.; Eberhart, A. J.; Procter, D. J. Mechanism of SmI_2/Amine/H_2O-Promoted Chemoselective Reductions of Carboxylic Acid Derivatives (Esters, Acids, and Amides) to Alcohols. *J. Org. Chem.* **2014**, *79*, 11988–12003.

8 Kawasaki, Y.; Rashid, S.; Ikeyatsu, K.; Mutoh, Y.; Yoshigoe, Y.; Kikkawa, S.; Azumaya, I.; Hosoya, S.; Saito, S. Conformational Control of [2]Rotaxane by Hydrogen Bond. *J. Org. Chem.* **2022**, *87*, 5744–5759.

11. NMR spectra

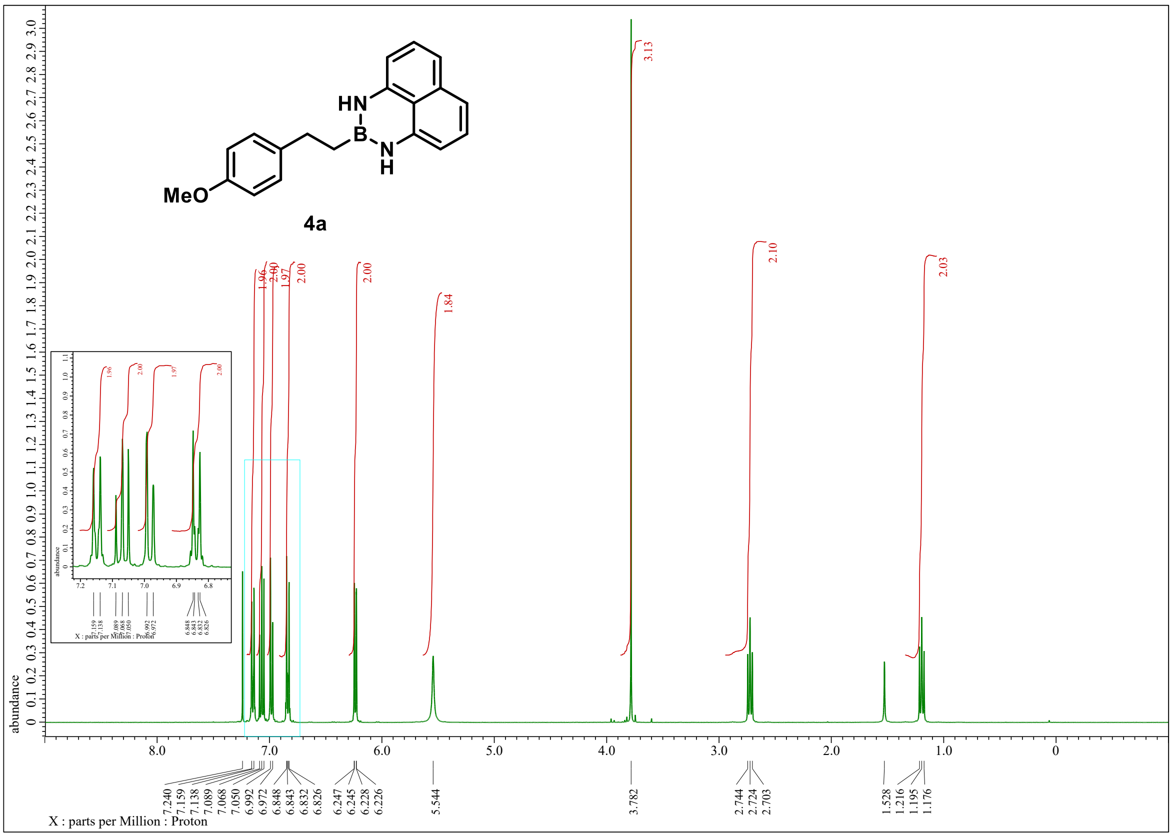

Figure S6. ^{1}H NMR ($CDCl_3$, 400 MHz) spectrum of **4a**.

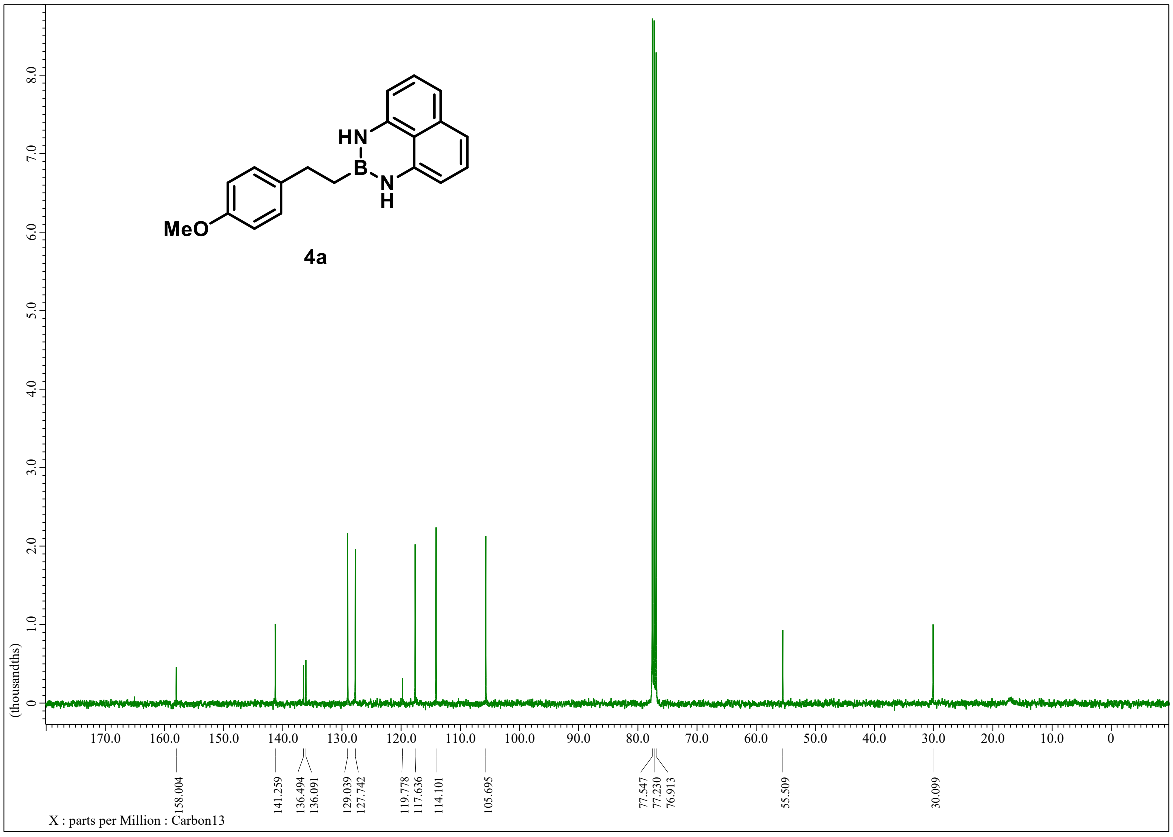

Figure S7. $^{13}C\{^{1}H\}$ NMR ($CDCl_3$, 100 MHz) spectrum of **4a**.

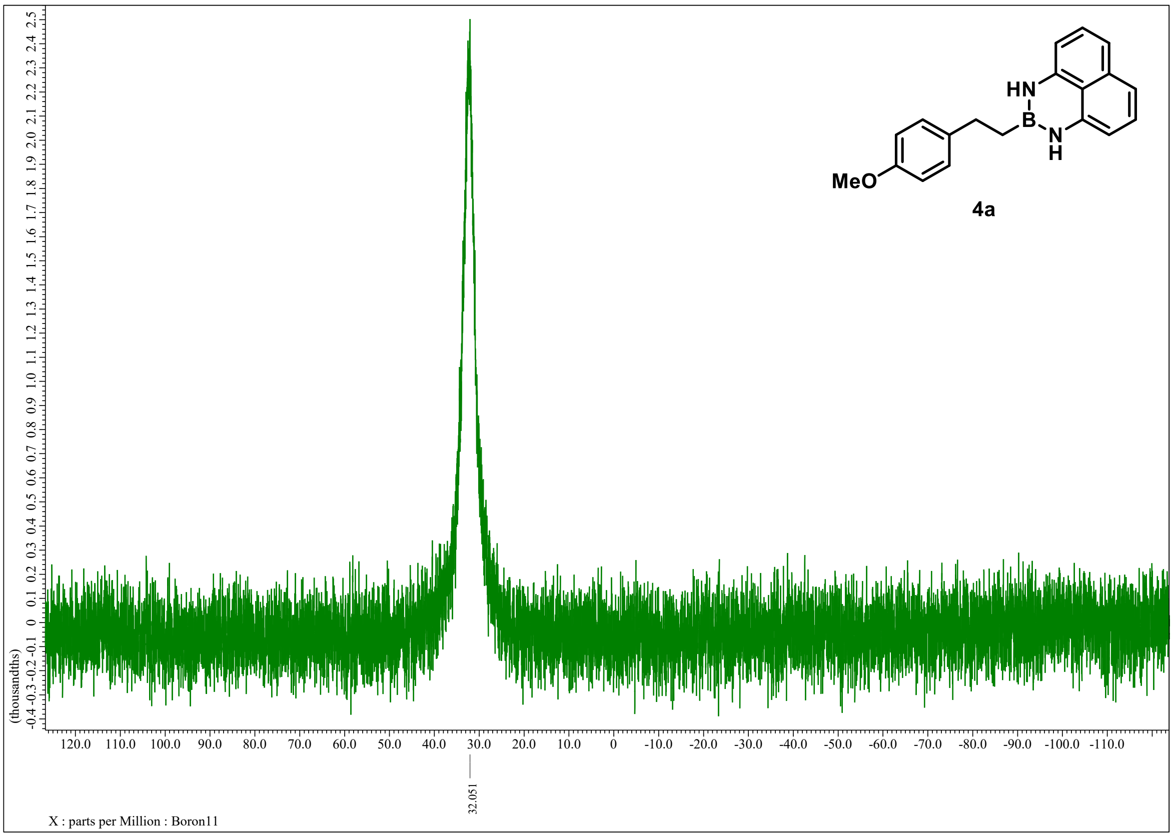

Figure S8. $^{11}B\{^{1}H\}$ NMR (128 MHz, $CDCl_3$) spectrum of **4a**.

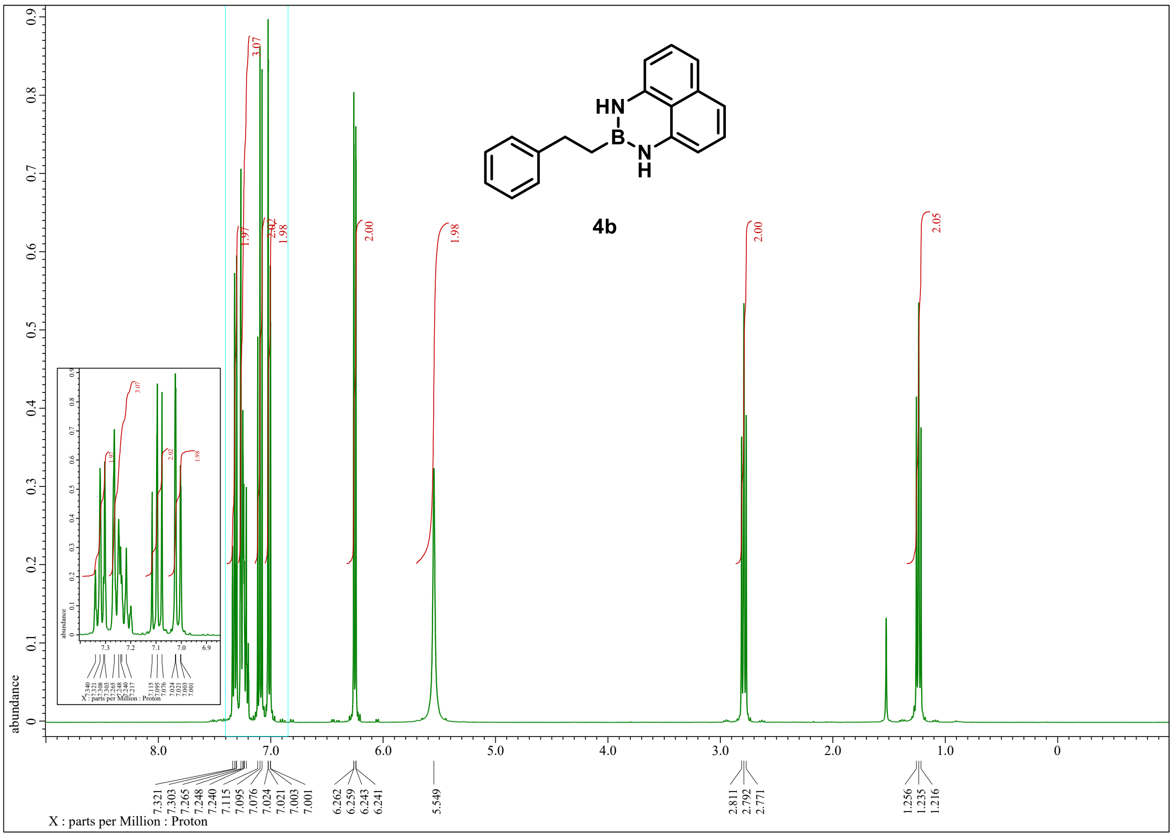

Figure S9. ^{1}H NMR (400 MHz, $CDCl_3$) spectrum of **4b**.

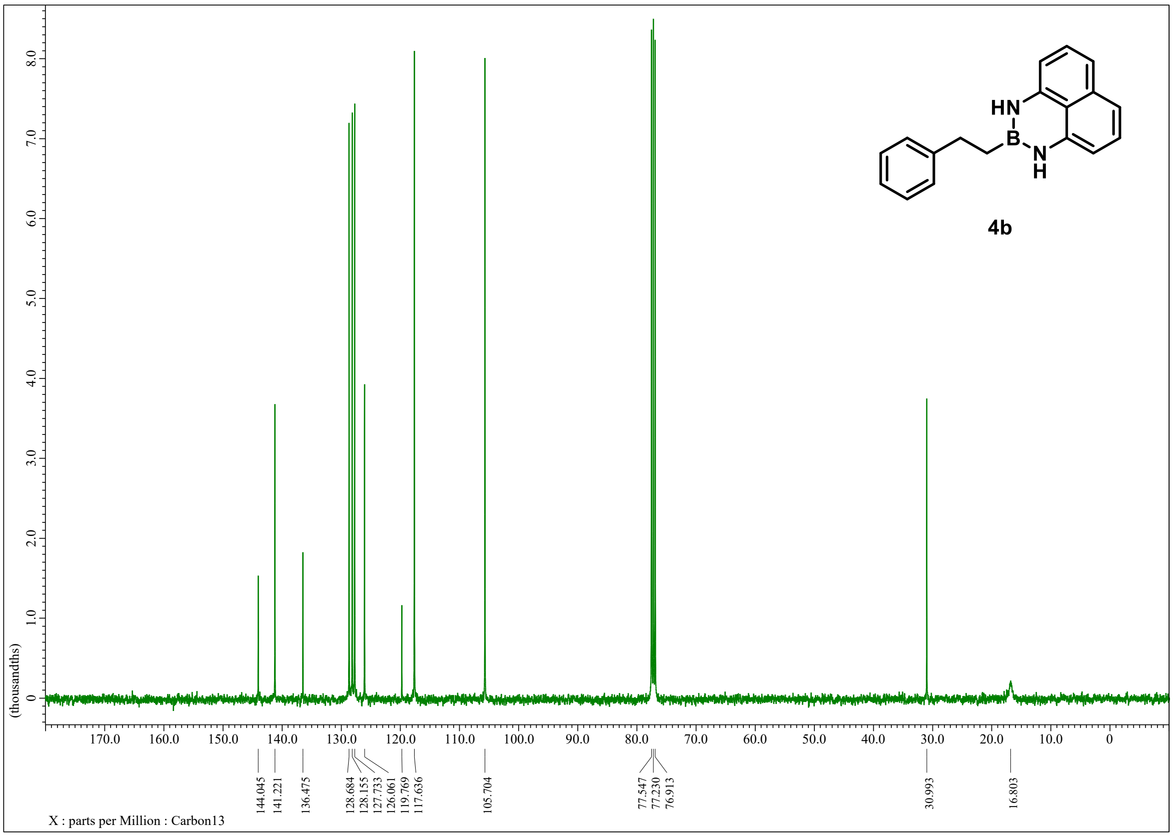

Figure S10. $^{13}C\{^{1}H\}$ NMR (100 MHz, $CDCl_3$) spectrum of **4b**.

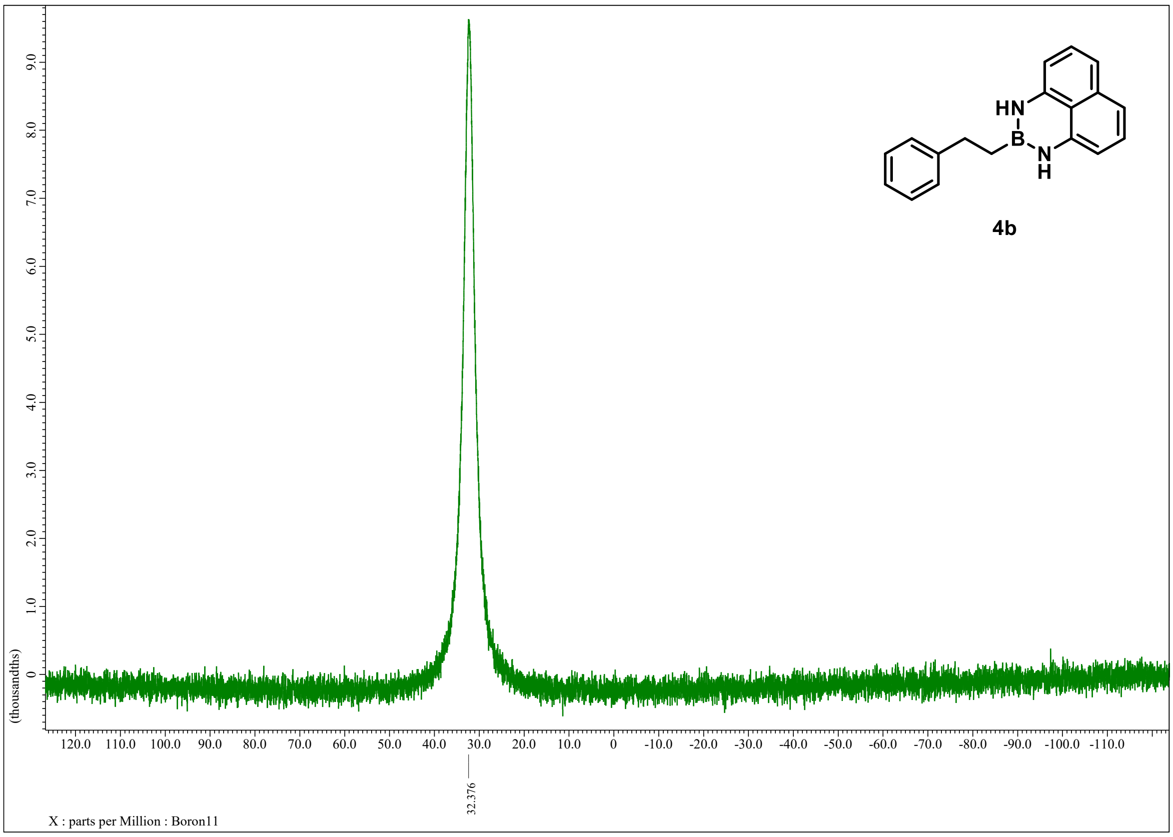

Figure S11. $^{11}B\{^{1}H\}$ NMR (128 MHz, $CDCl_3$) spectrum of **4b**.

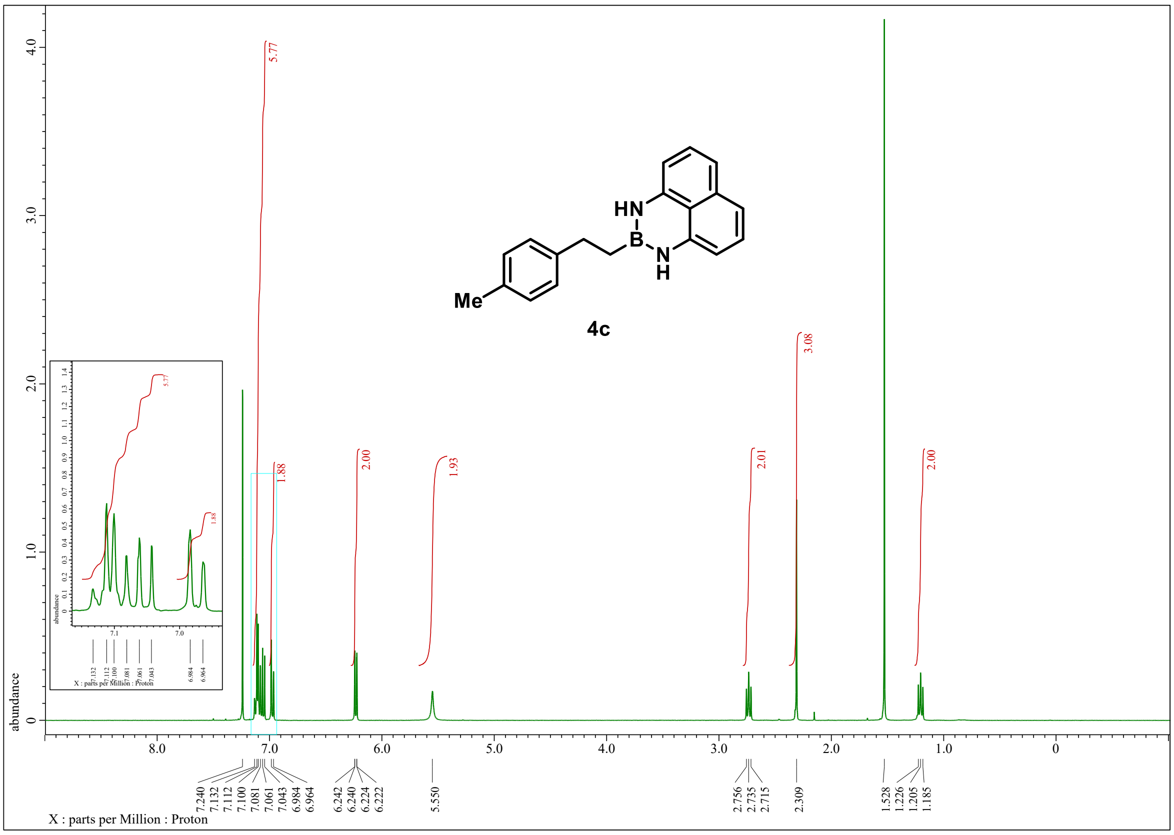

Figure S12. ^{1}H NMR (400 MHz, $CDCl_3$) spectrum of **4c**.

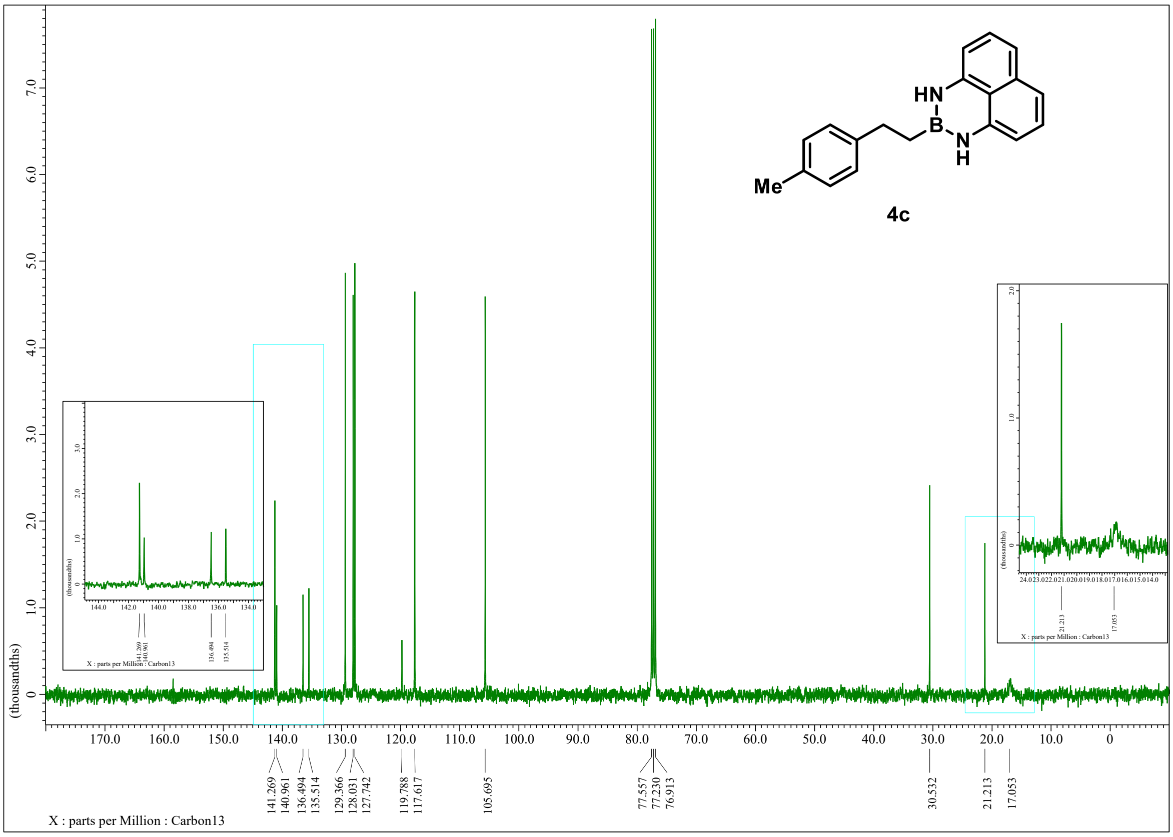

Figure S13. $^{13}C\{^{1}H\}$ NMR (100 MHz, $CDCl_3$) spectrum of **4c**.

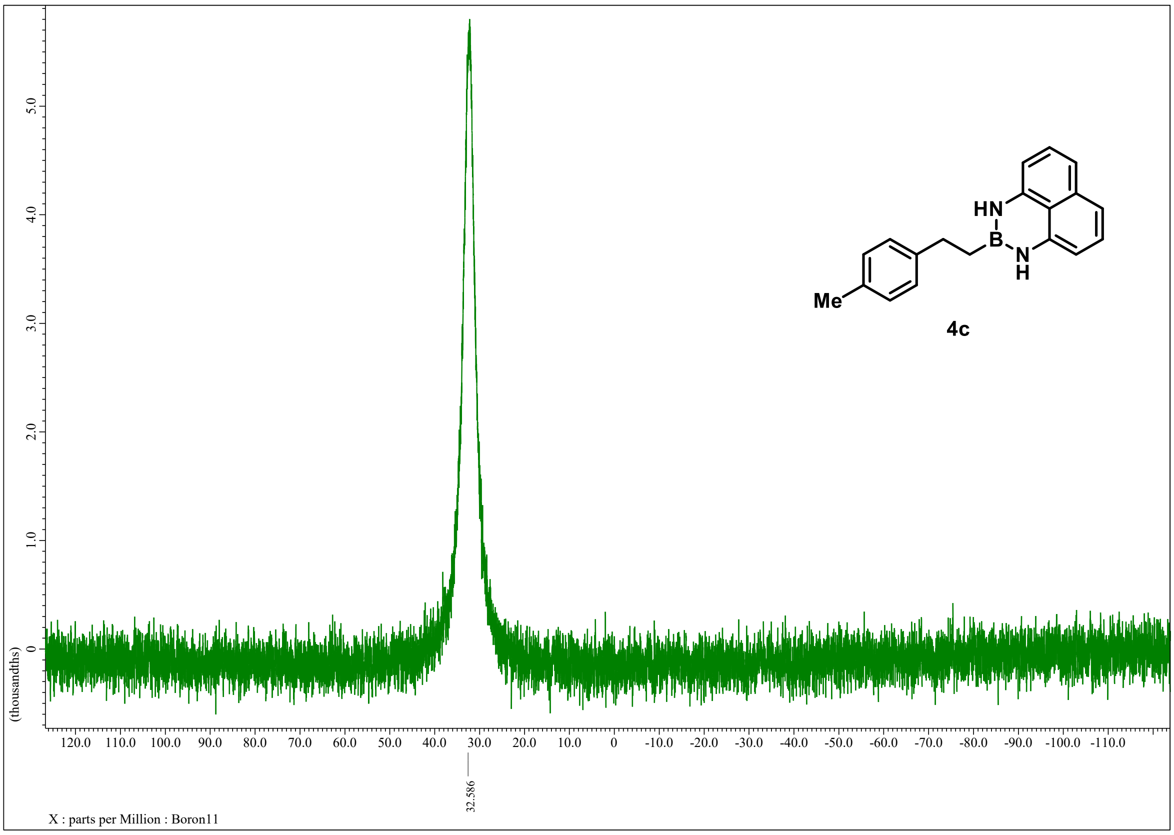

Figure S14. $^{11}B\{^{1}H\}$ NMR (128 MHz, $CDCl_3$) spectrum of **4c.**

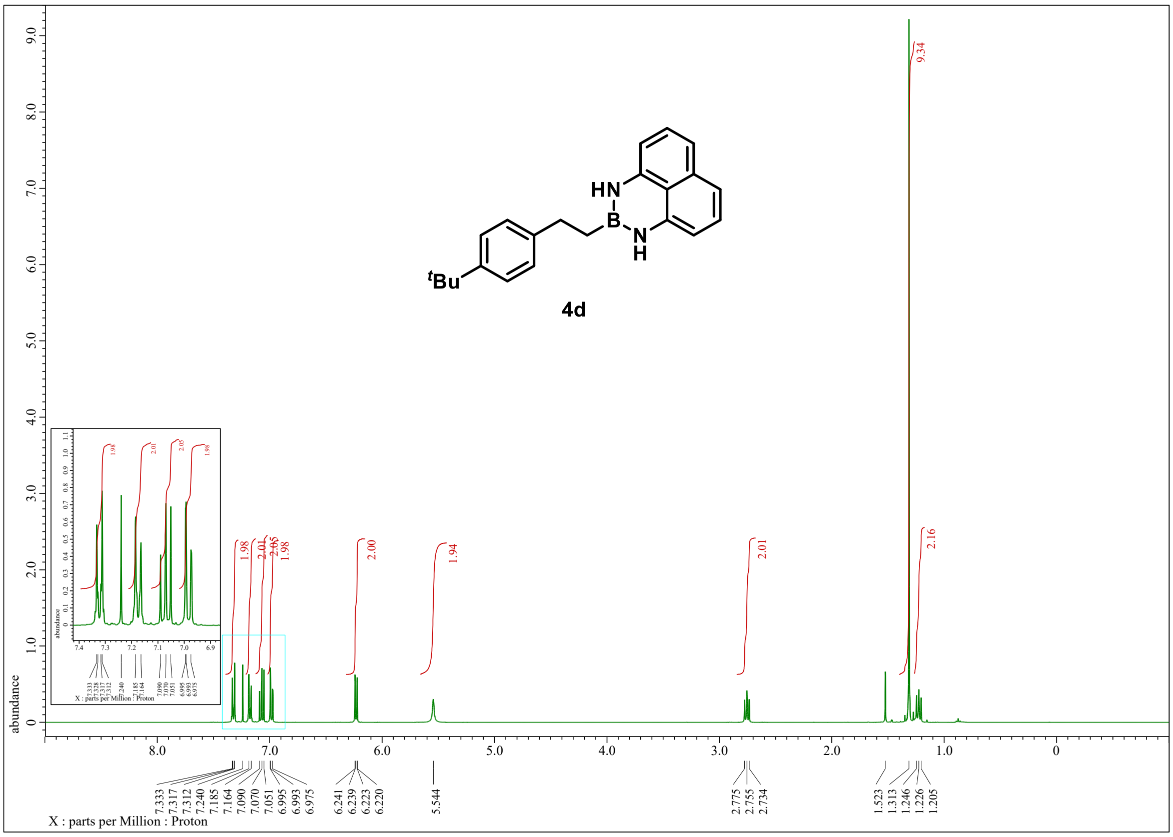

Figure S15. ^{1}H NMR (400 MHz, $CDCl_3$) spectrum of **4d.**

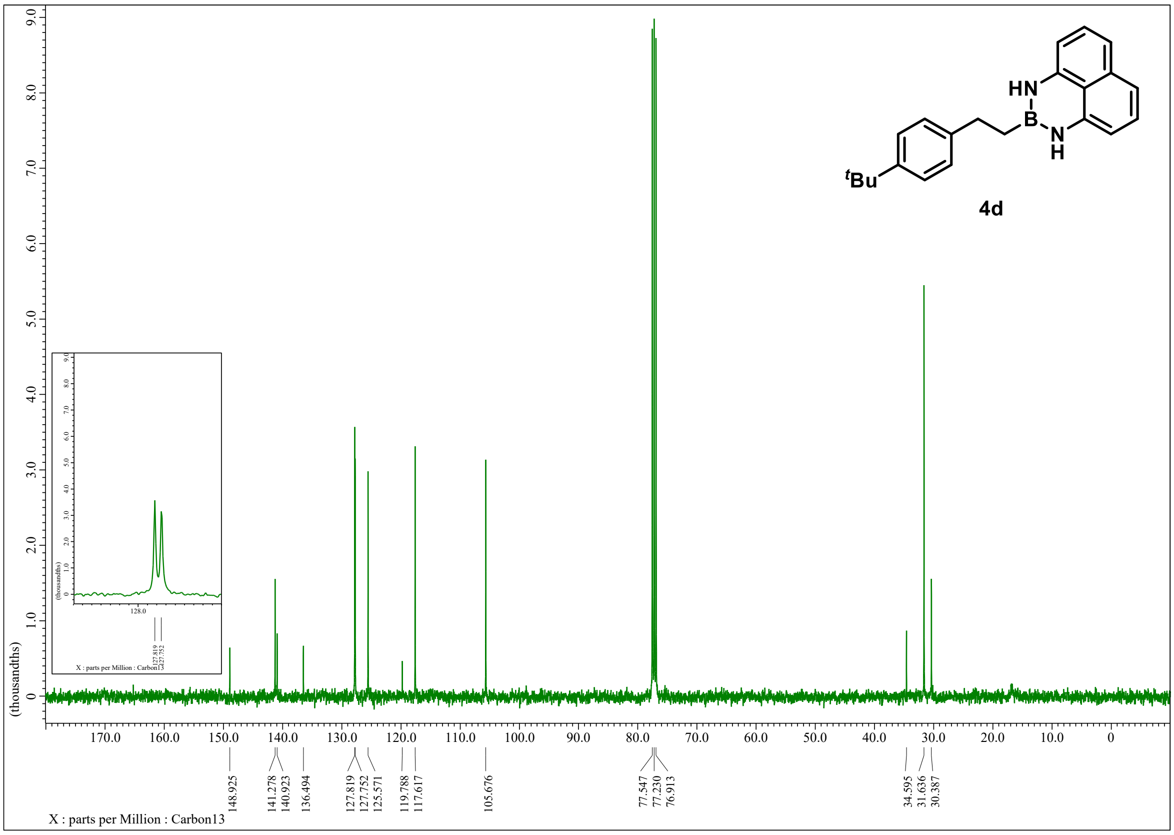

Figure S16. $^{13}C\{^{1}H\}$ NMR (100 MHz, $CDCl_3$) spectrum of **4d.**

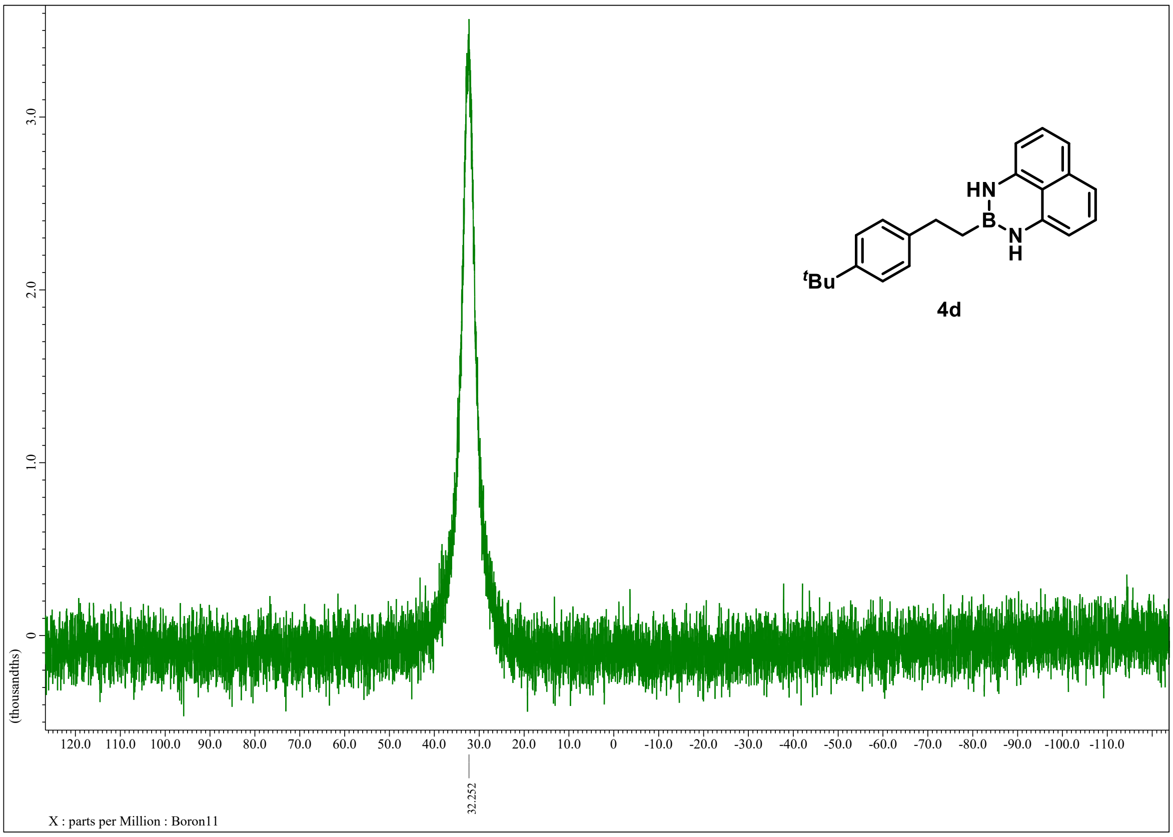

Figure S17. $^{11}B\{^{1}H\}$ NMR (128 MHz, $CDCl_3$) spectrum of **4d**.

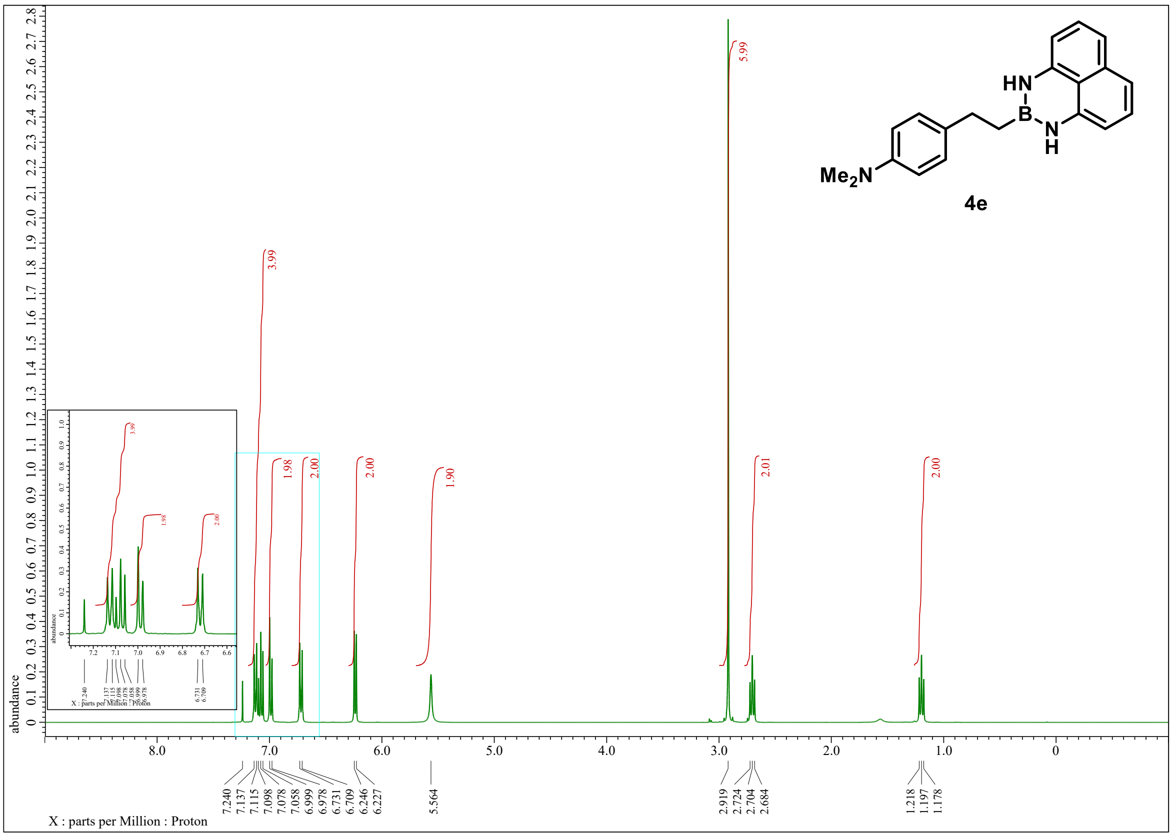

Figure S18. ^{1}H NMR (400 MHz, $CDCl_3$) spectrum of **4e.**

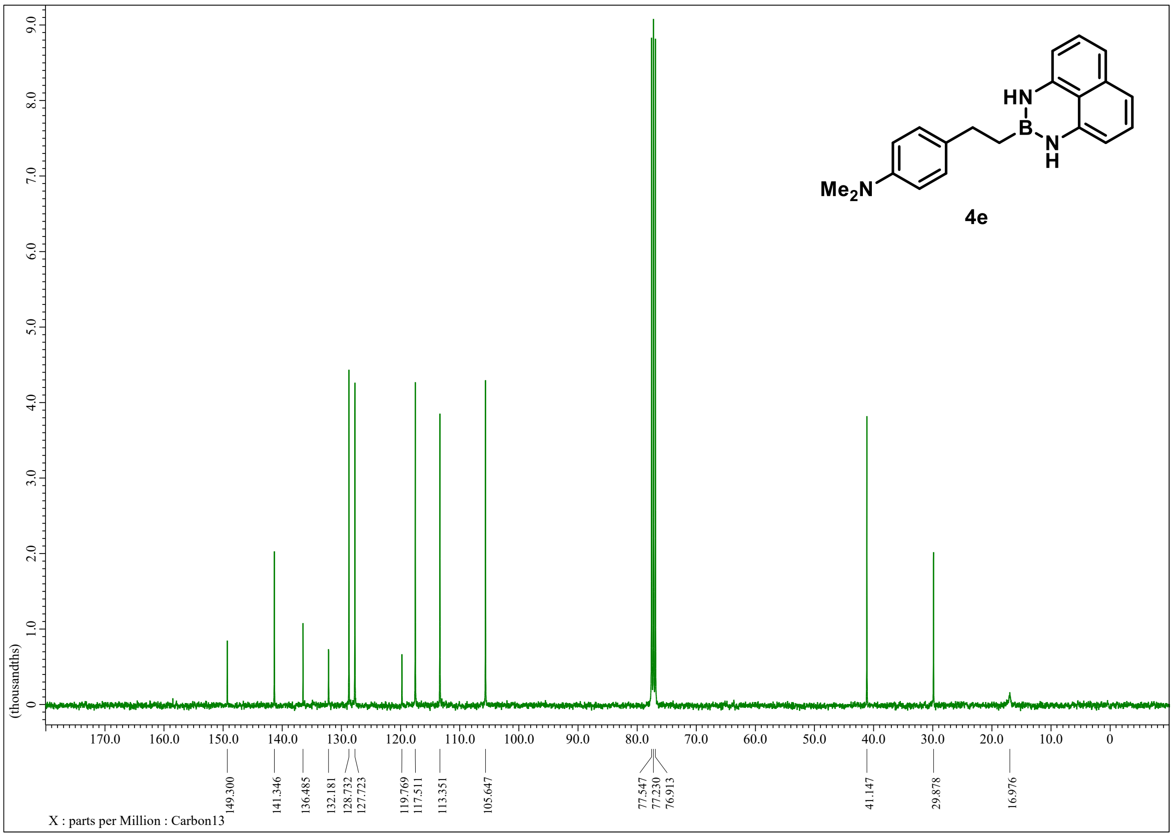

Figure S19. $^{13}C\{^{1}H\}$ NMR (100 MHz, $CDCl_3$) spectrum of **4e.**

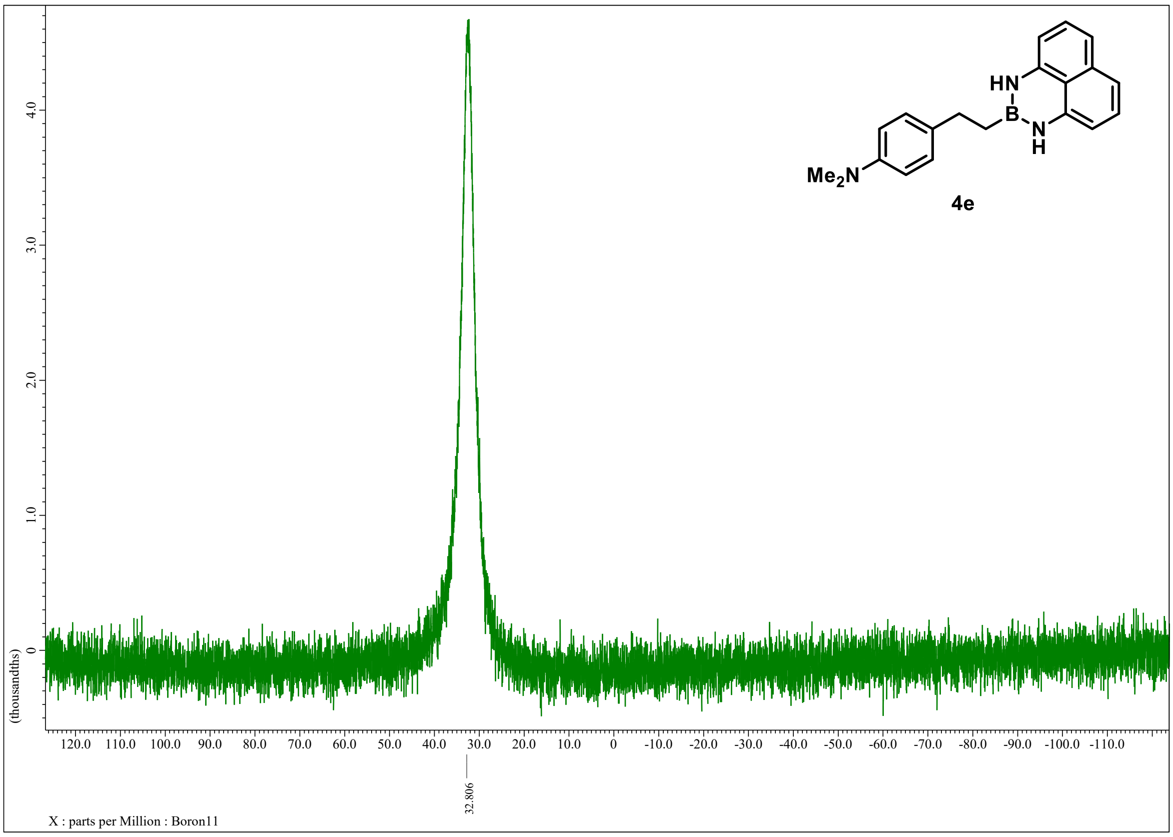

Figure S20. $^{11}B\{^1H\}$ NMR (128 MHz, $CDCl_3$) spectrum of **4e.**

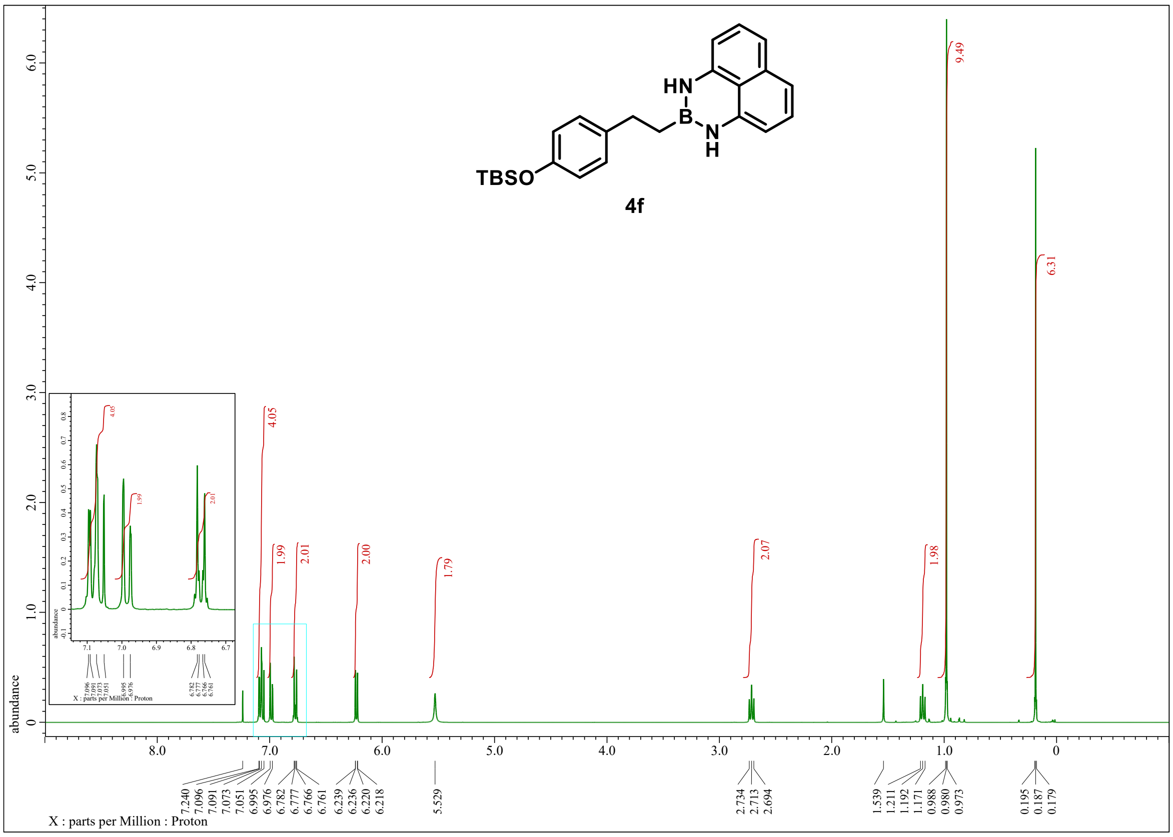

Figure S21. ^{1}H NMR (400 MHz, $CDCl_3$) spectrum of **4f**.

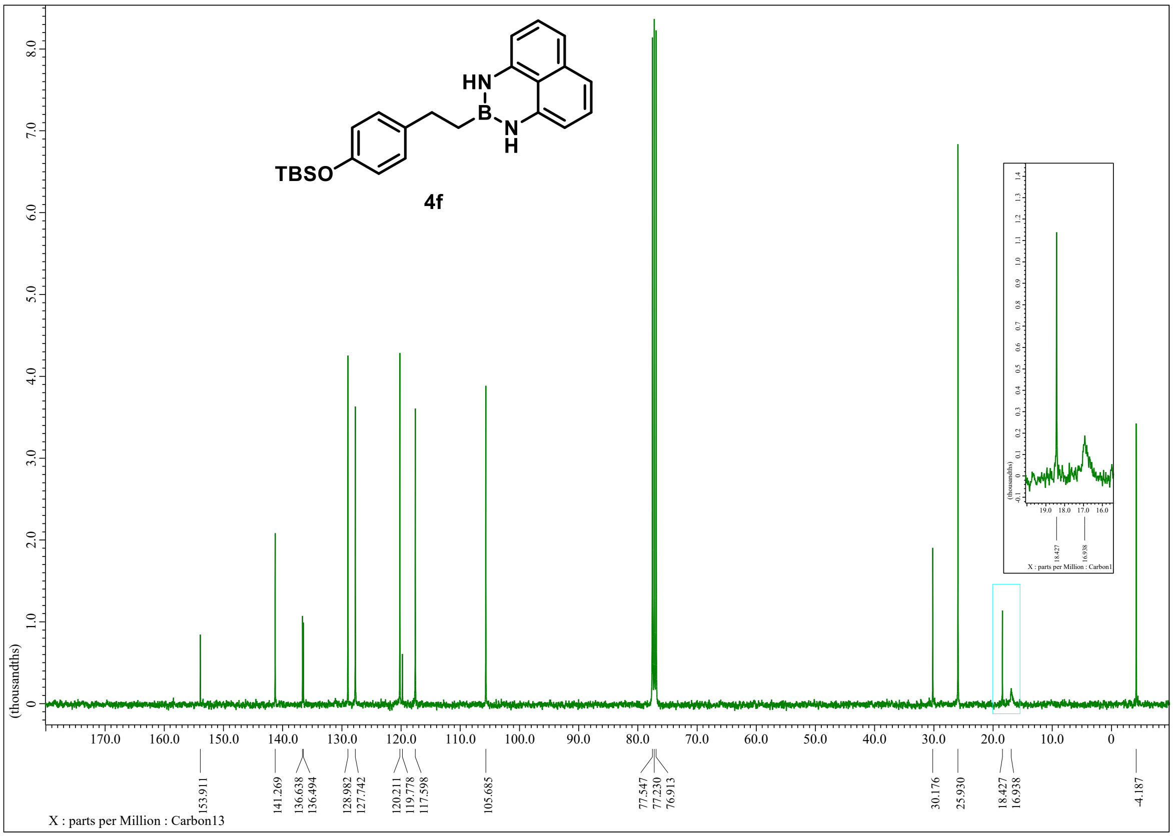

Figure S22. $^{13}C\{^{1}H\}$ NMR (100 MHz, $CDCl_3$) spectrum of **4f**.

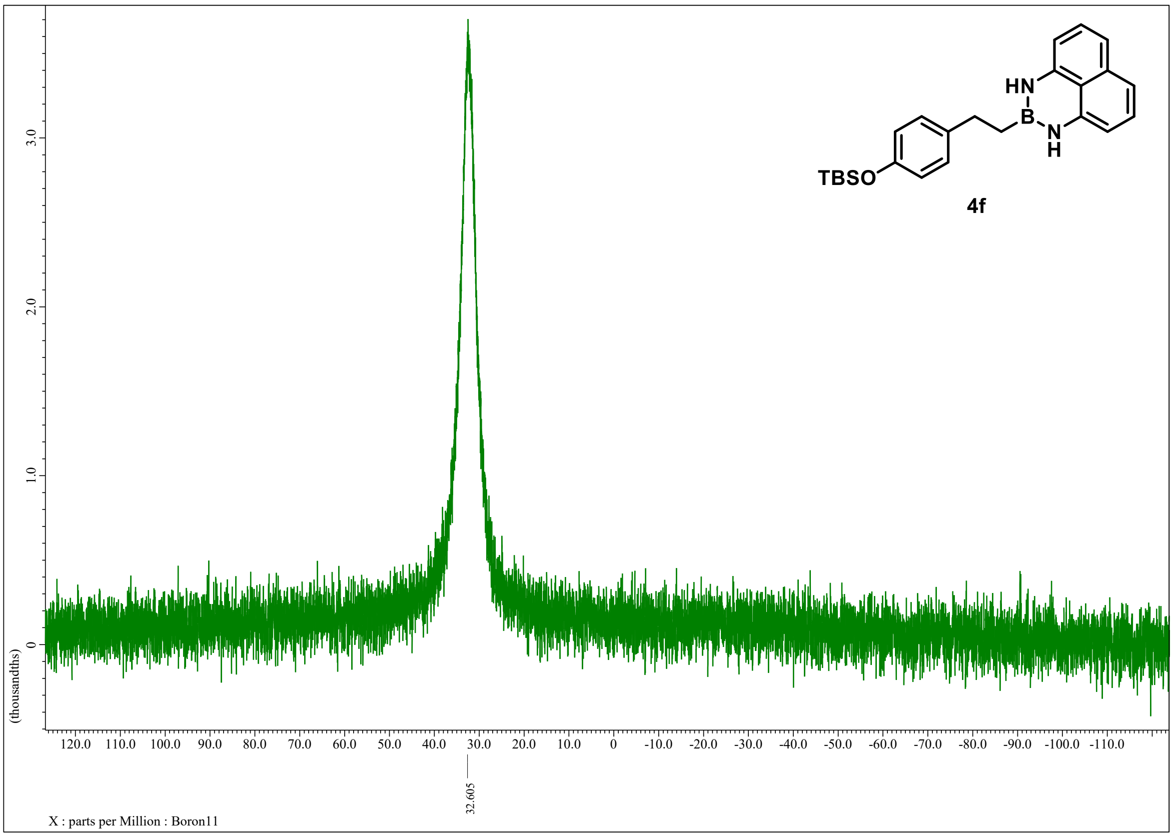

Figure S23. $^{11}B\{^{1}H\}$ NMR (128 MHz, $CDCl_3$) spectrum of **4f**.

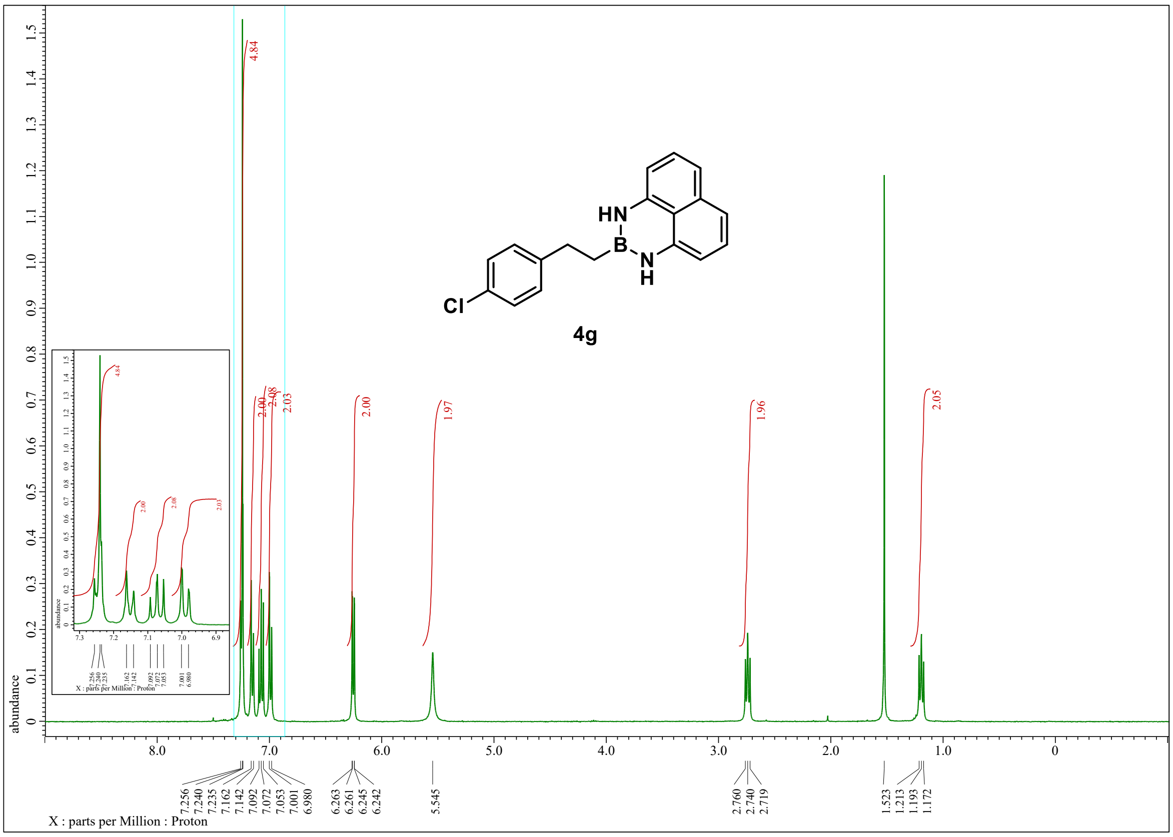

Figure S24. ^{1}H NMR (400 MHz, $CDCl_3$) spectrum of **4g**.

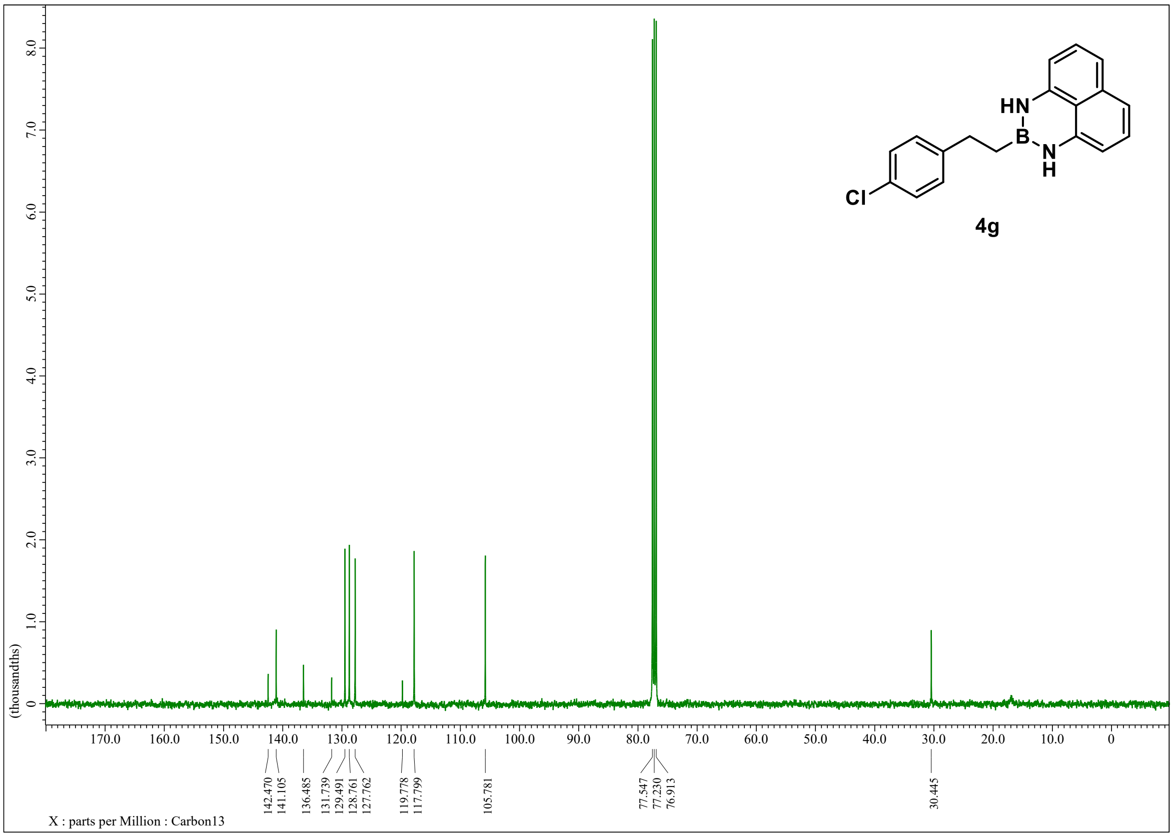

Figure S25. $^{13}C\{^{1}H\}$ NMR (100 MHz, $CDCl_3$) spectrum of **4f**.

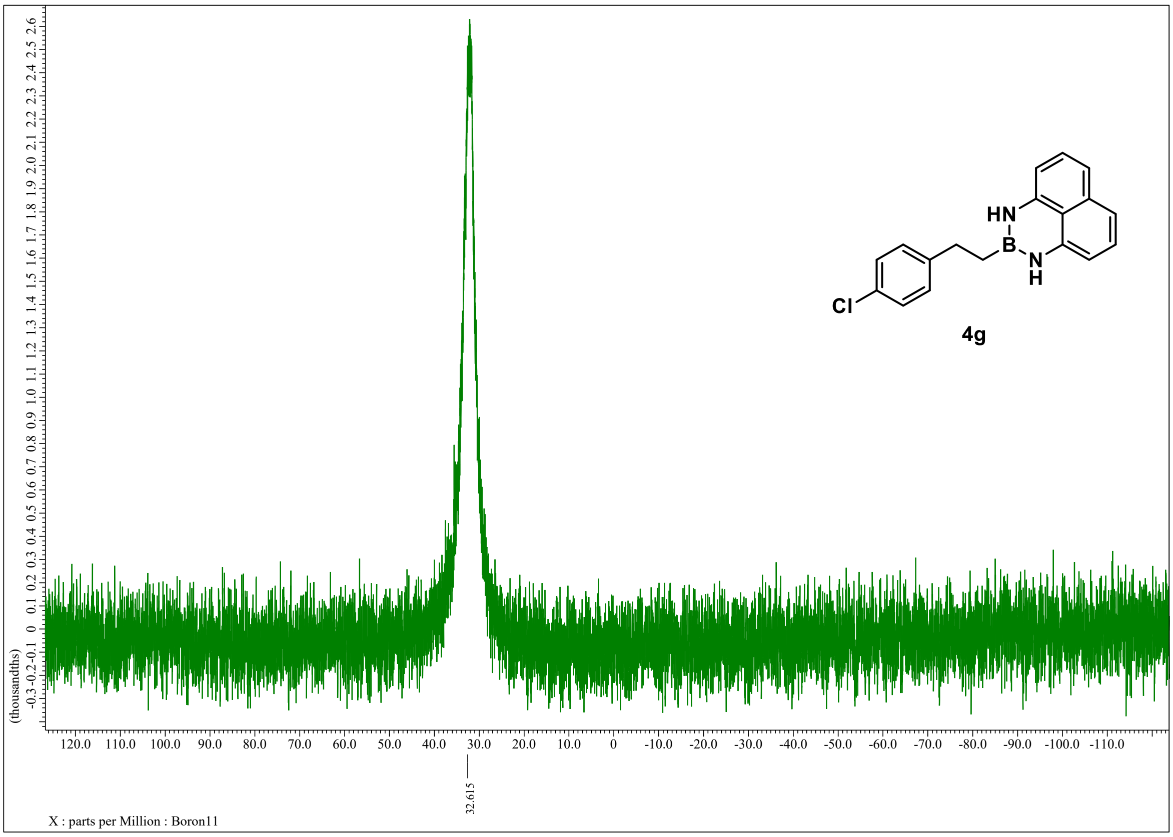

Figure S26. $^{11}B\{^{1}H\}$ NMR (128 MHz, $CDCl_3$) spectrum of **4g**.

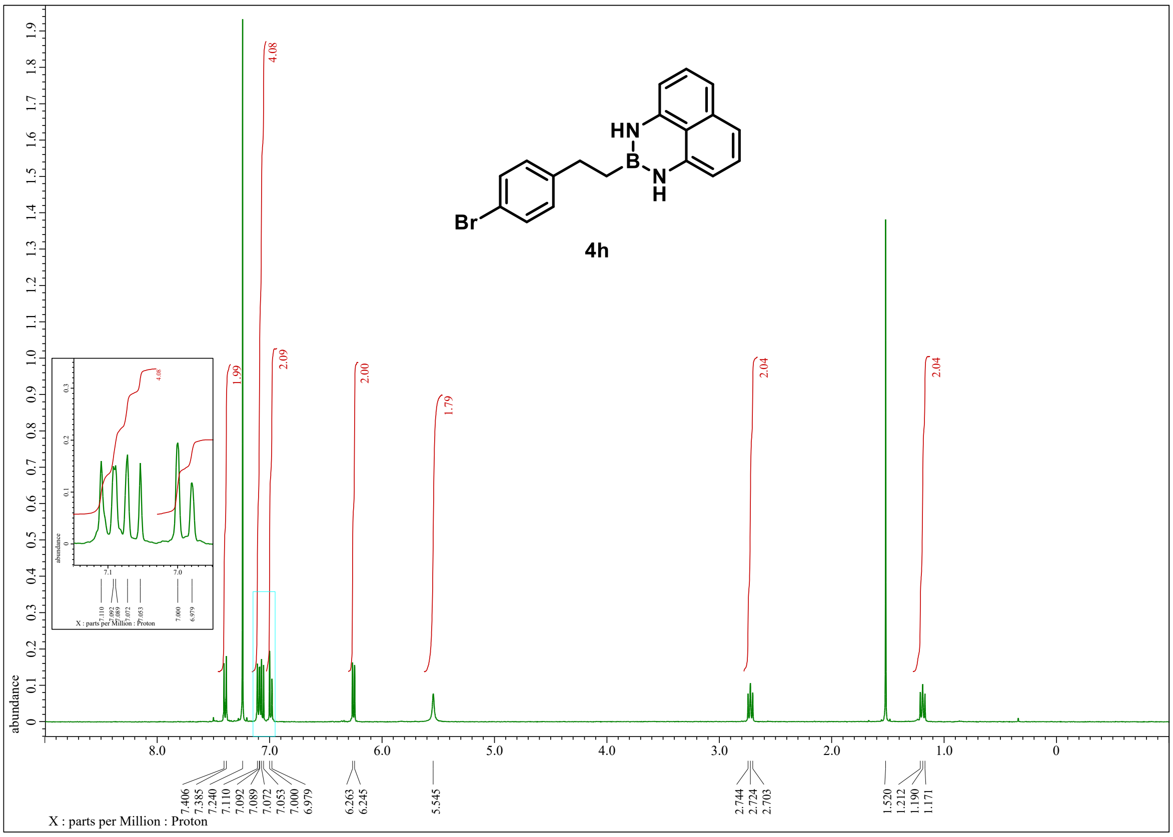

Figure S27. ^{1}H NMR (400 MHz, $CDCl_3$) spectrum of **4h**.

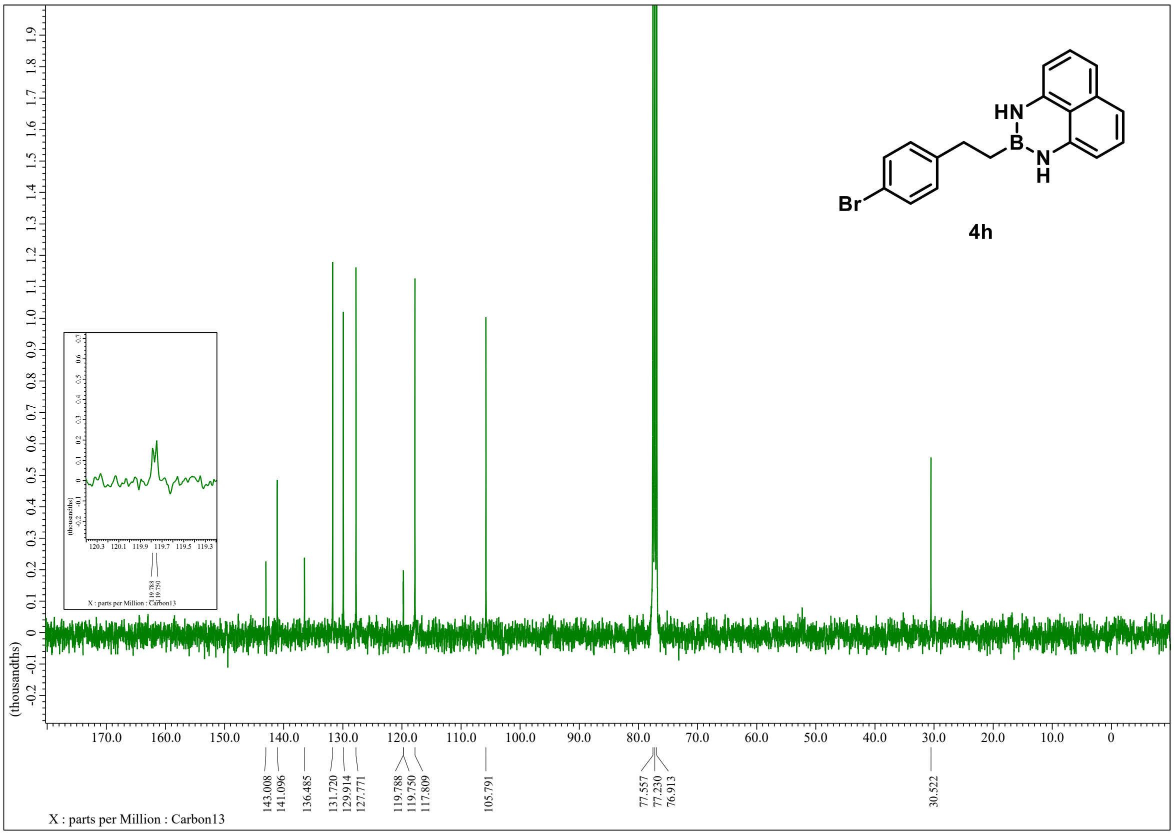

Figure S28. $^{13}C\{^{1}H\}$ NMR (100 MHz, $CDCl_3$) spectrum of **4h**.

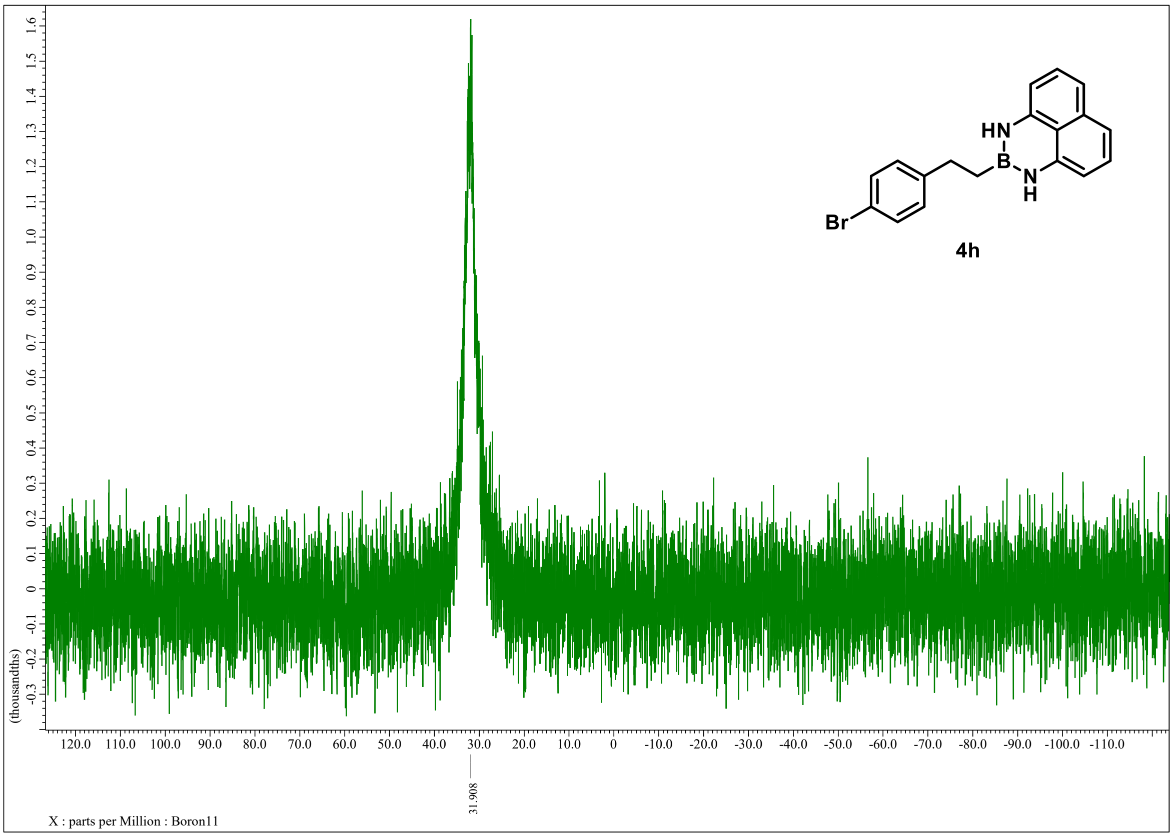

Figure S29. $^{11}B\{^{1}H\}$ NMR (128 MHz, $CDCl_3$) spectrum of **4h**.

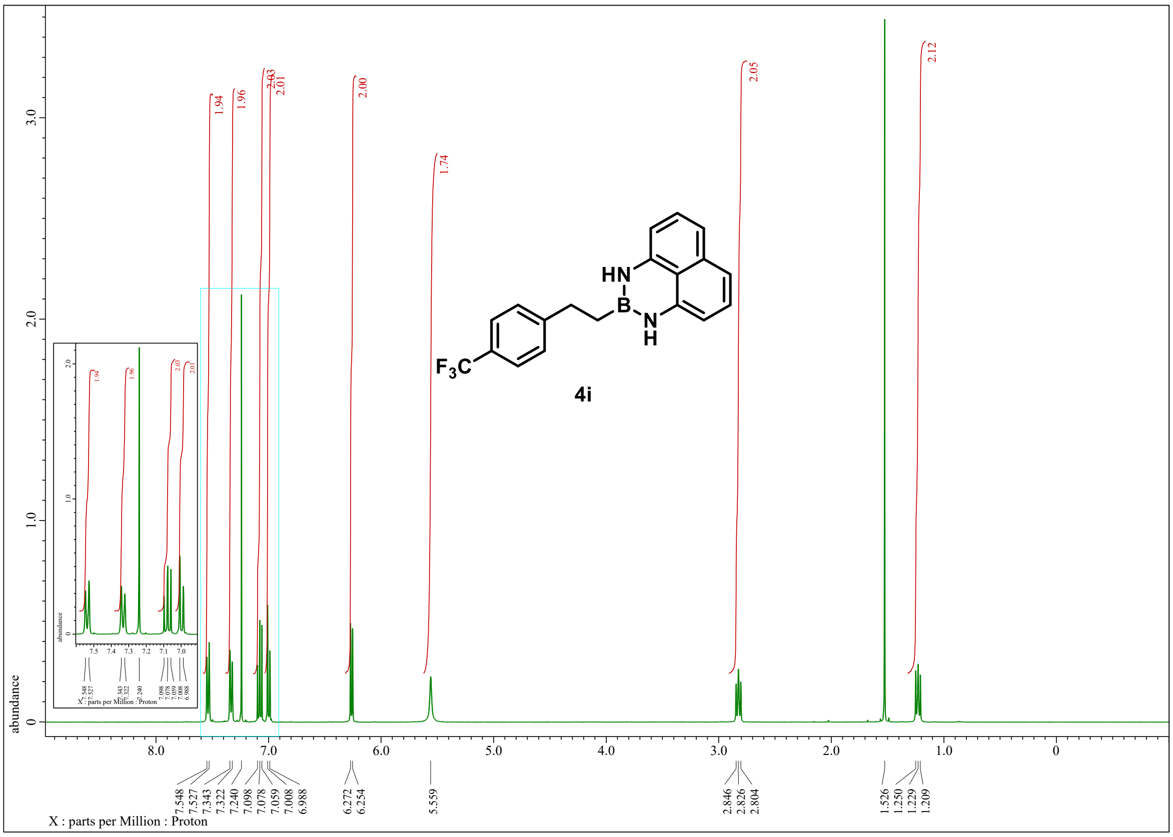

Figure S30. ^{1}H NMR (400 MHz, $CDCl_3$) spectrum of **4i**.

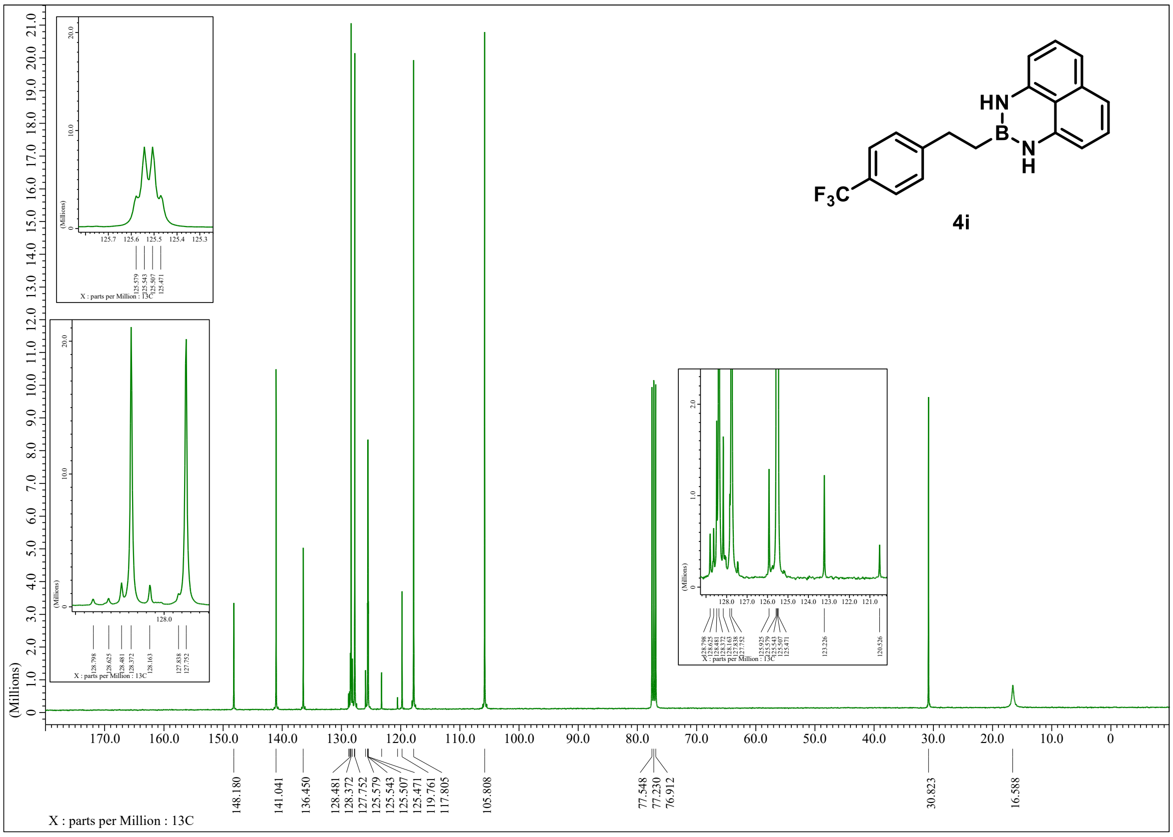

Figure S31. $^{13}C\{^{1}H\}$ NMR (100 MHz, $CDCl_3$) spectrum of **4i**.

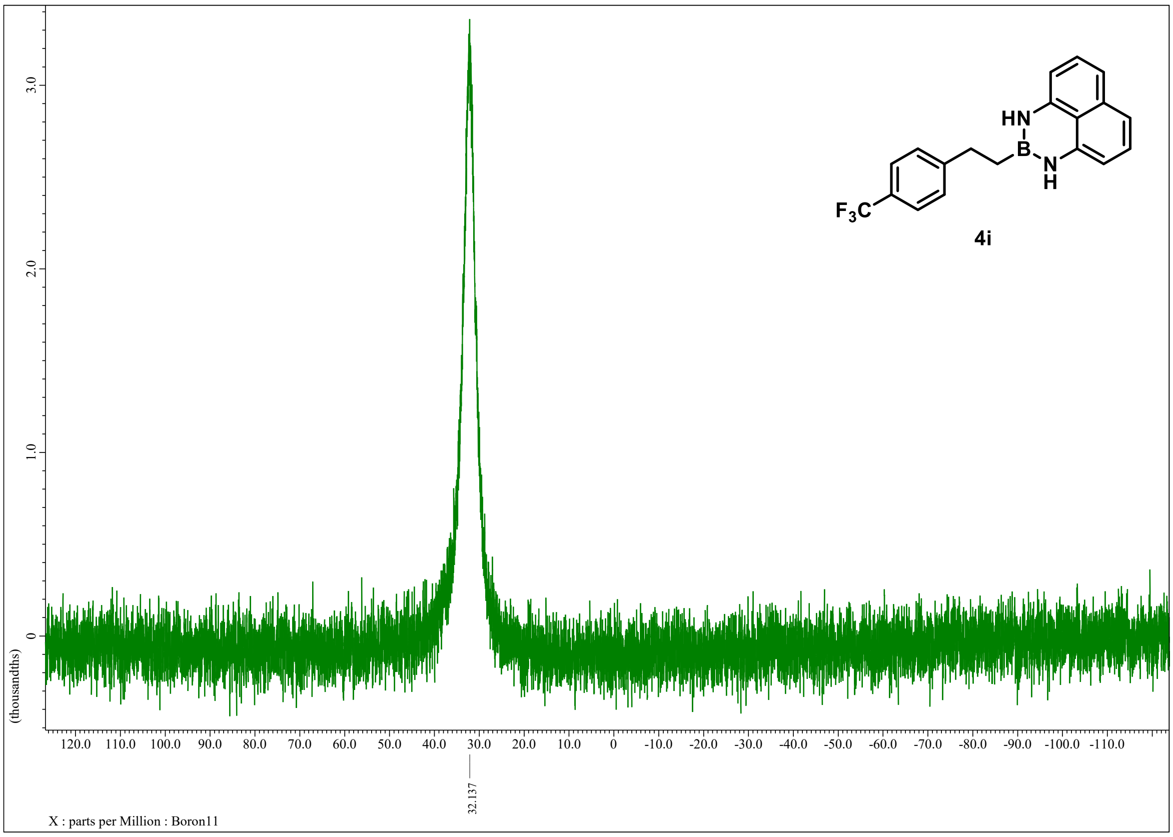

Figure S32. $^{11}B\{^{1}H\}$ NMR (128 MHz, $CDCl_3$) spectrum of **4i**.

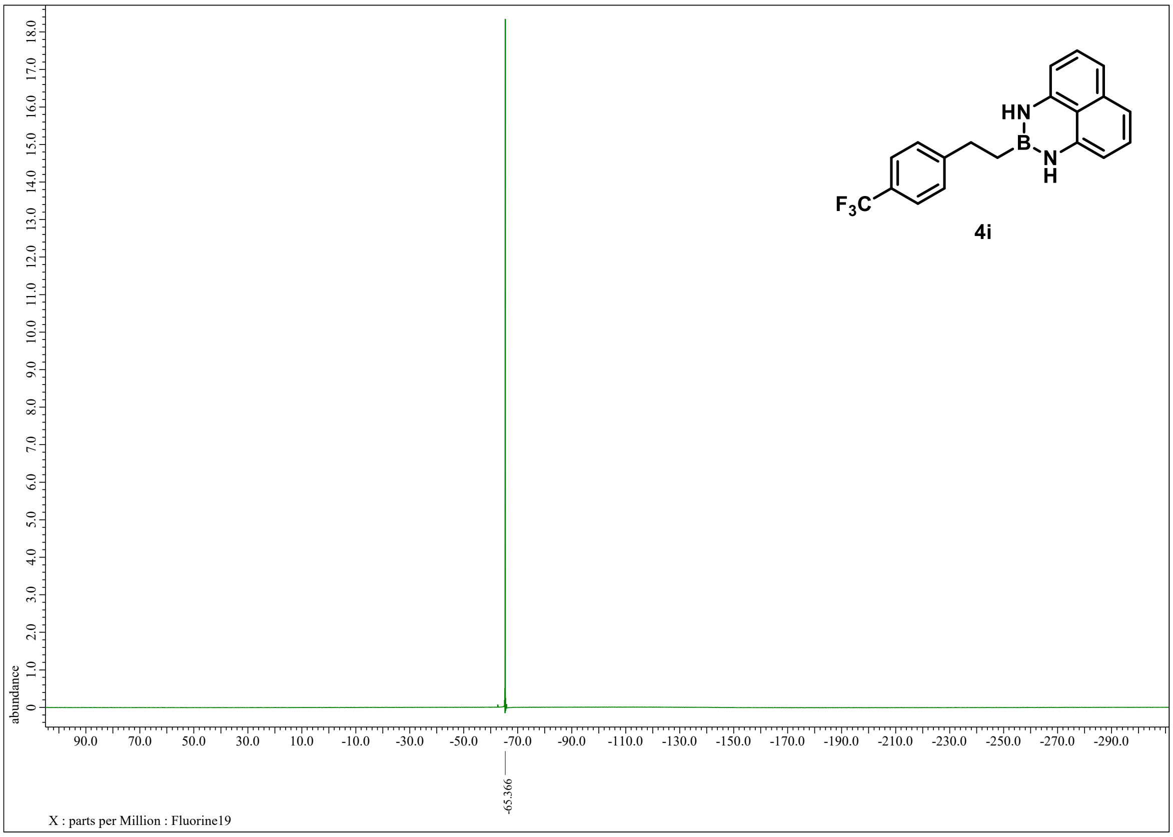

Figure S33. ^{19}F NMR (375 MHz, $CDCl_3$) spectrum of **4i**.

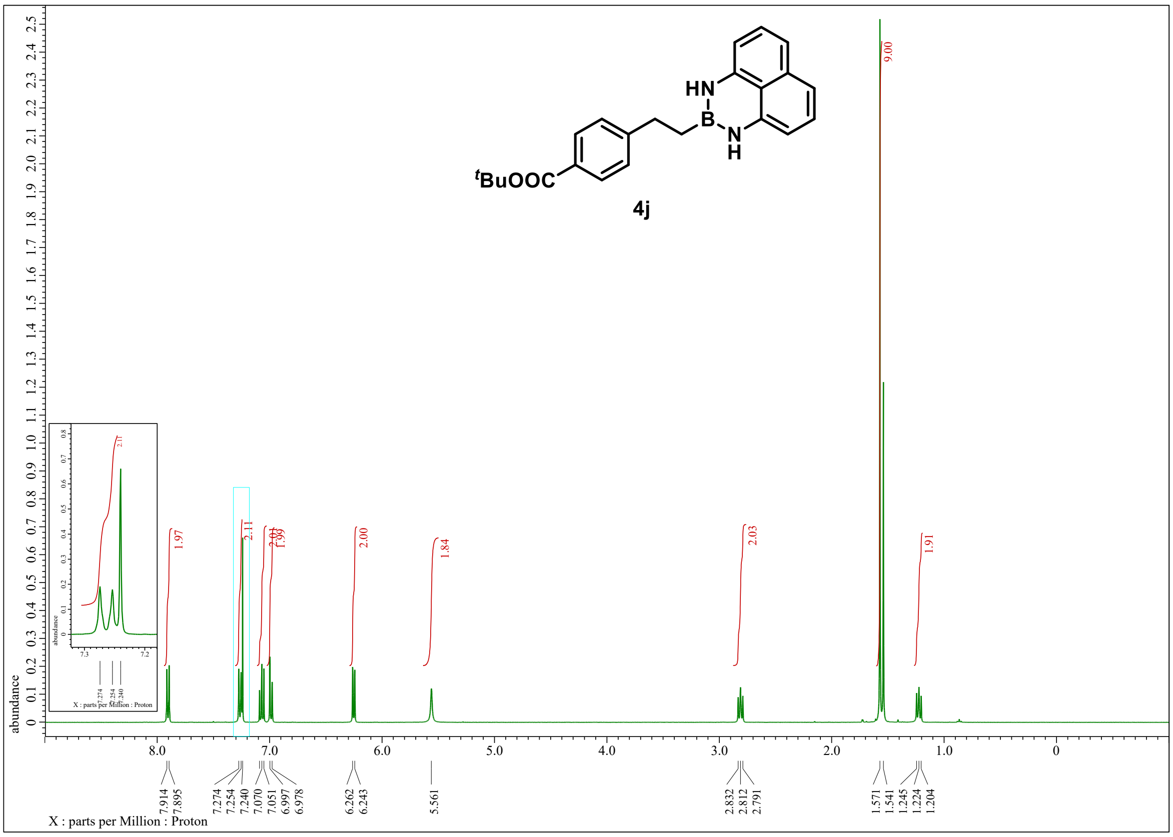

Figure S34. ^{1}H NMR (400 MHz, $CDCl_3$) spectrum of **4k**.

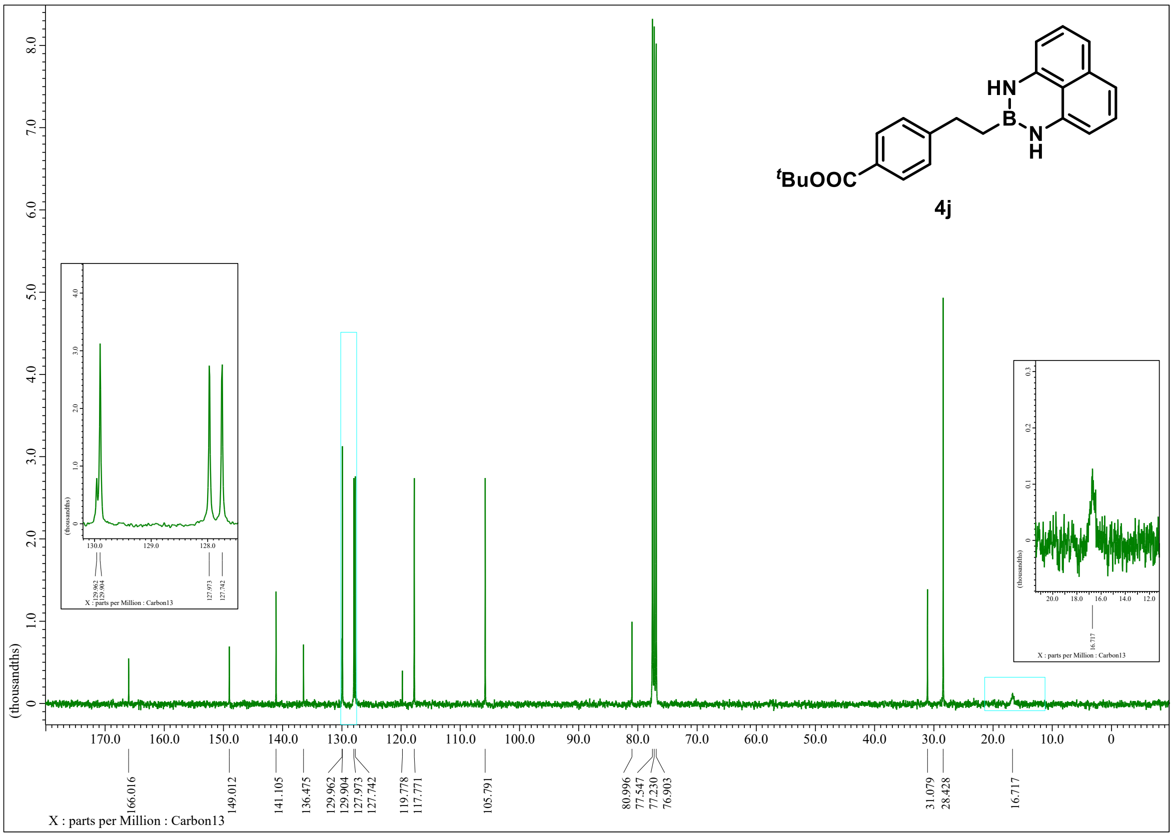

Figure S35. $^{13}C\{^{1}H\}$ NMR (100 MHz, $CDCl_3$) spectrum of **4j**.

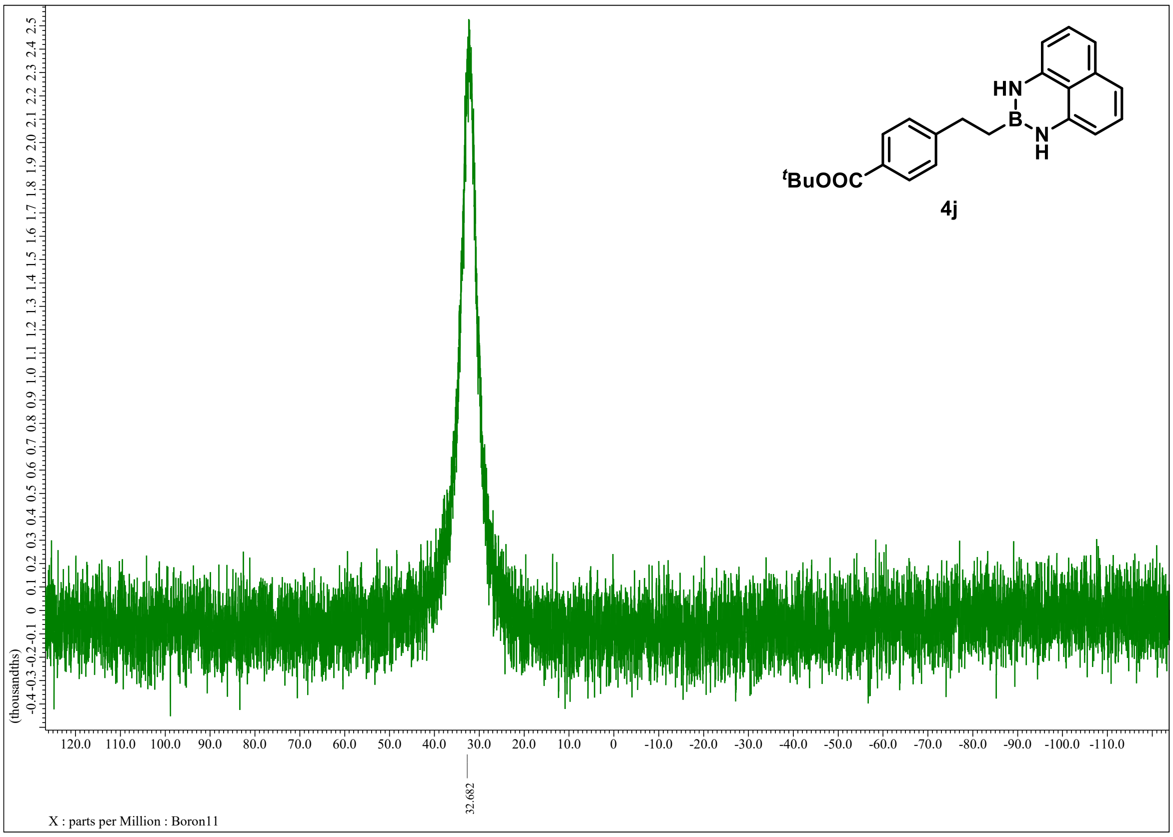

Figure S36. $^{11}B\{^{1}H\}$ NMR (128 MHz, $CDCl_3$) spectrum of **4j**.

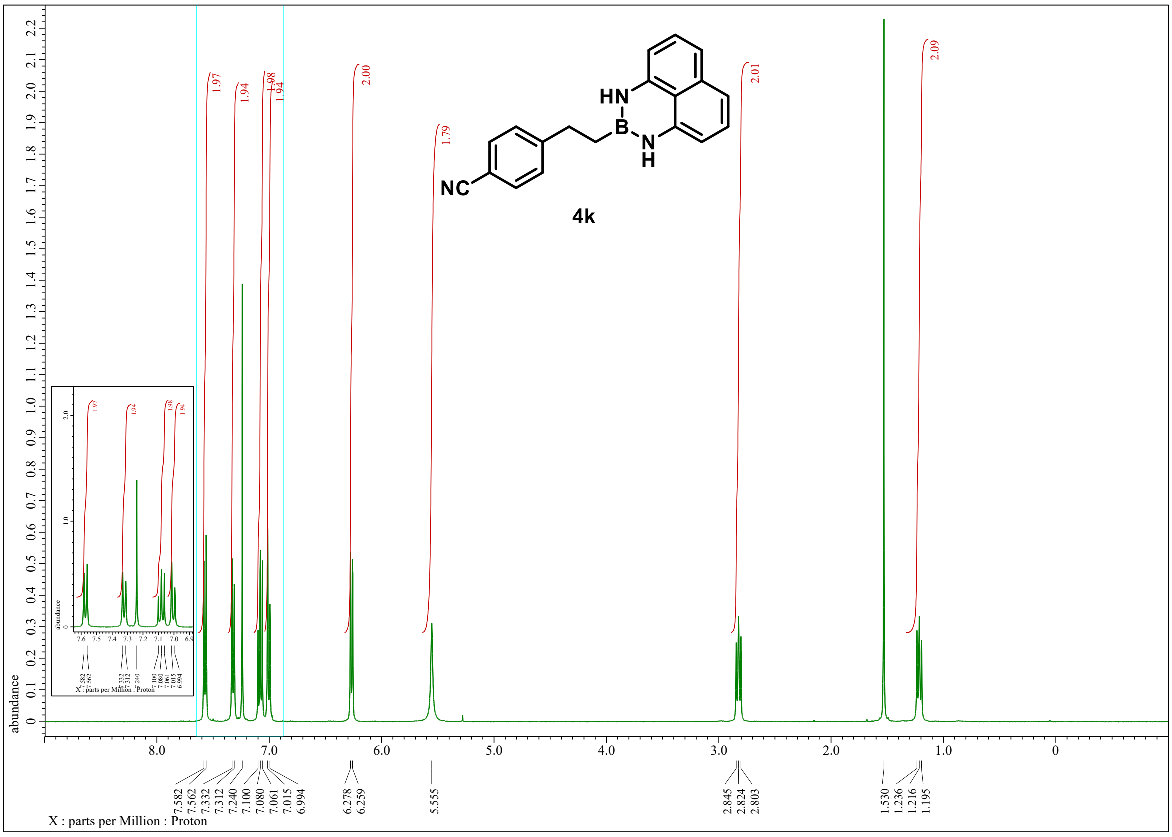

Figure S37. ^{1}H NMR (400 MHz, $CDCl_3$) spectrum of **4k**.

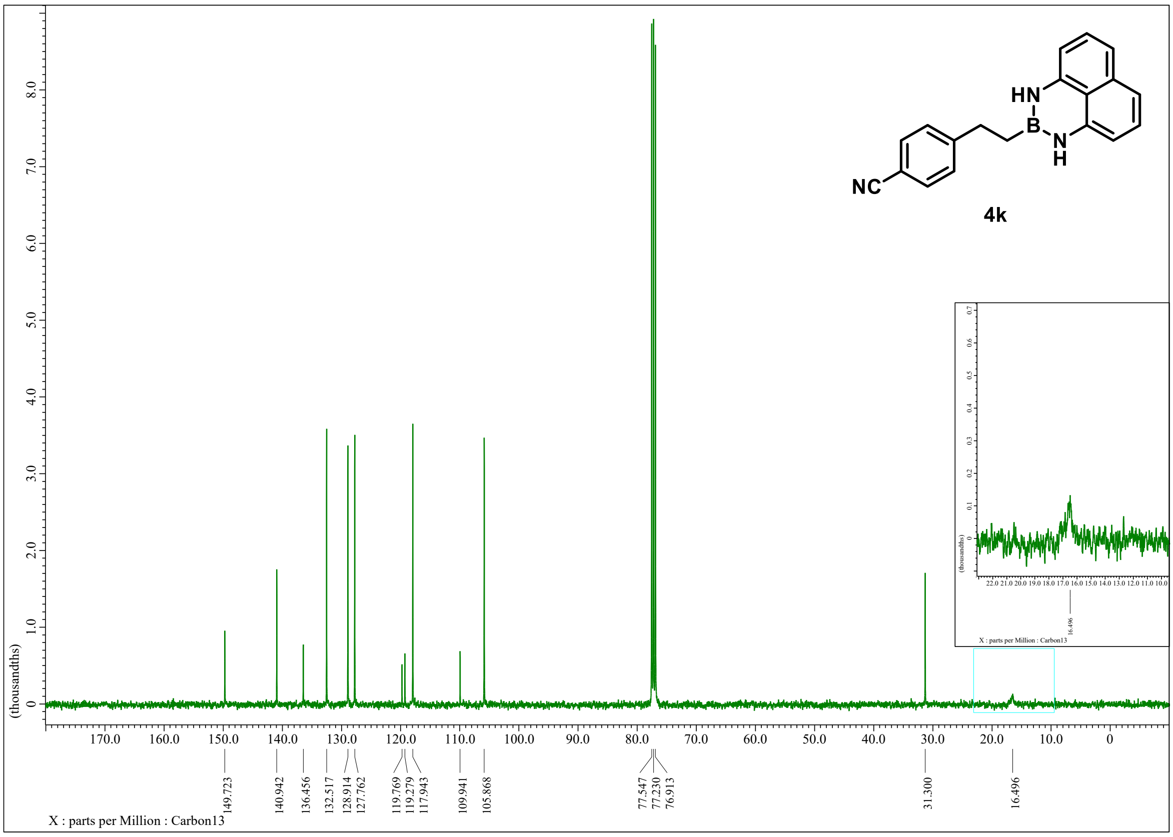

Figure S38. $^{13}C\{^{1}H\}$ NMR (100 MHz, $CDCl_3$) spectrum of **4k**.

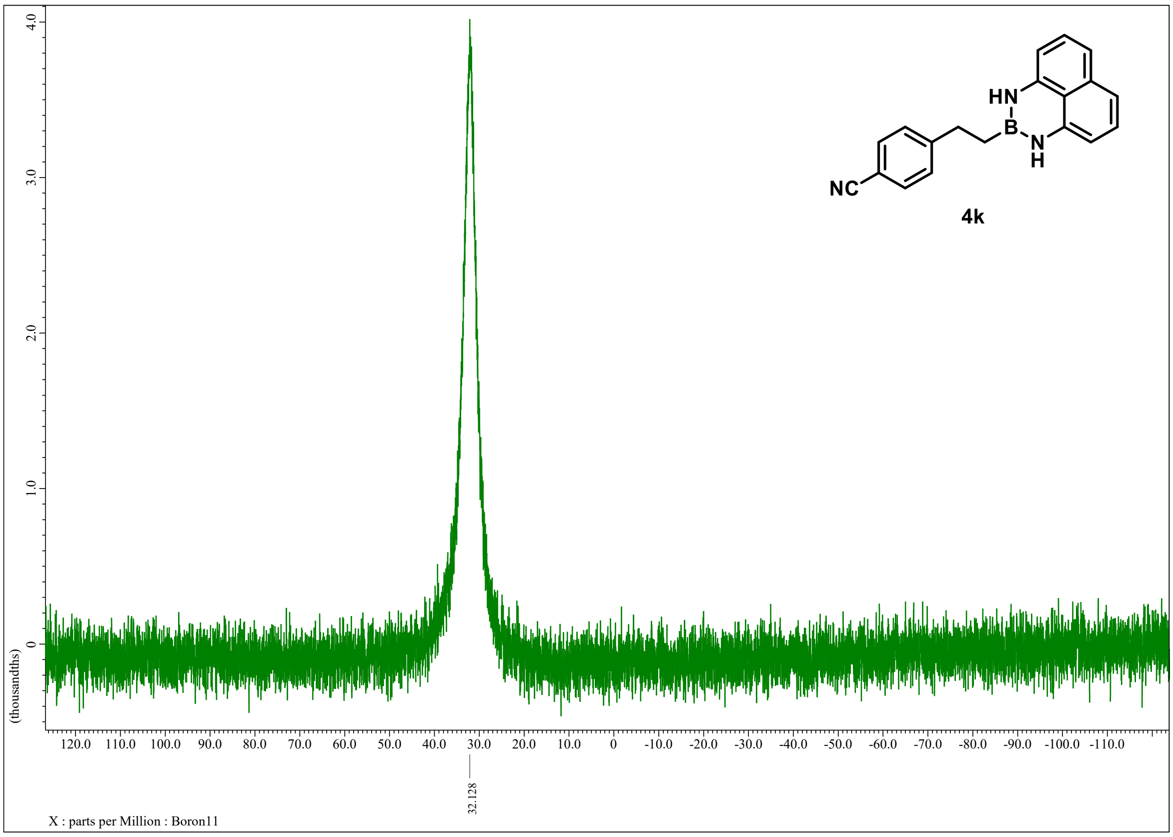

Figure S39. $^{11}B\{^{1}H\}$ NMR (128 MHz, $CDCl_3$) spectrum of **4k**.

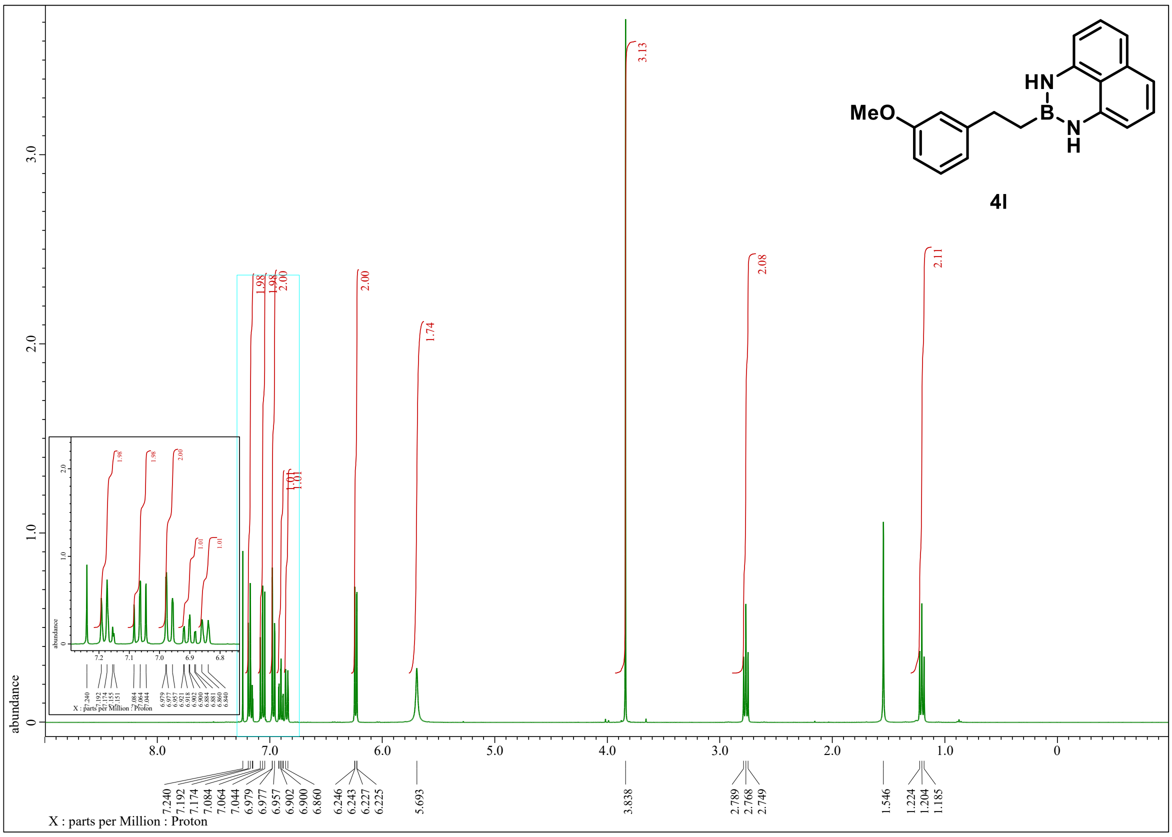

Figure S40. ^{1}H NMR (400 MHz, $CDCl_3$) spectrum of **4l**.

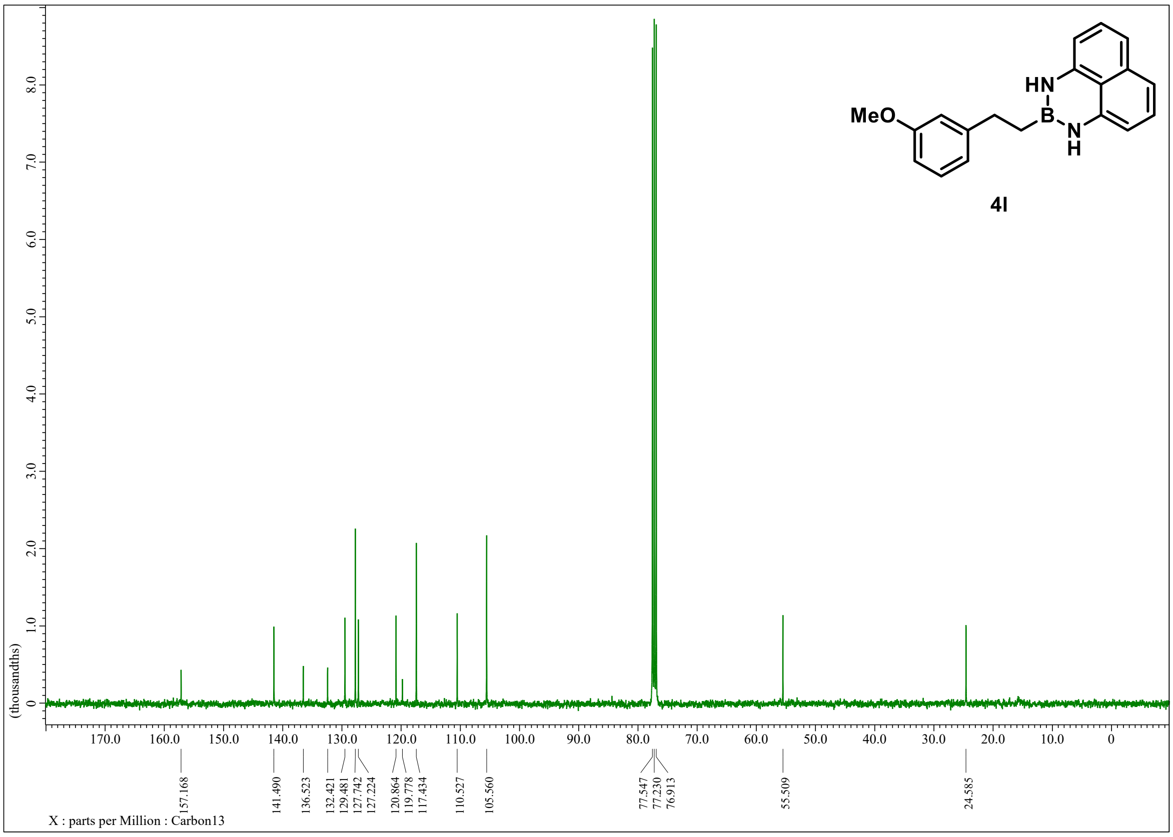

Figure S41. $^{13}C\{^{1}H\}$ NMR (100 MHz, $CDCl_3$) spectrum of **4l**.

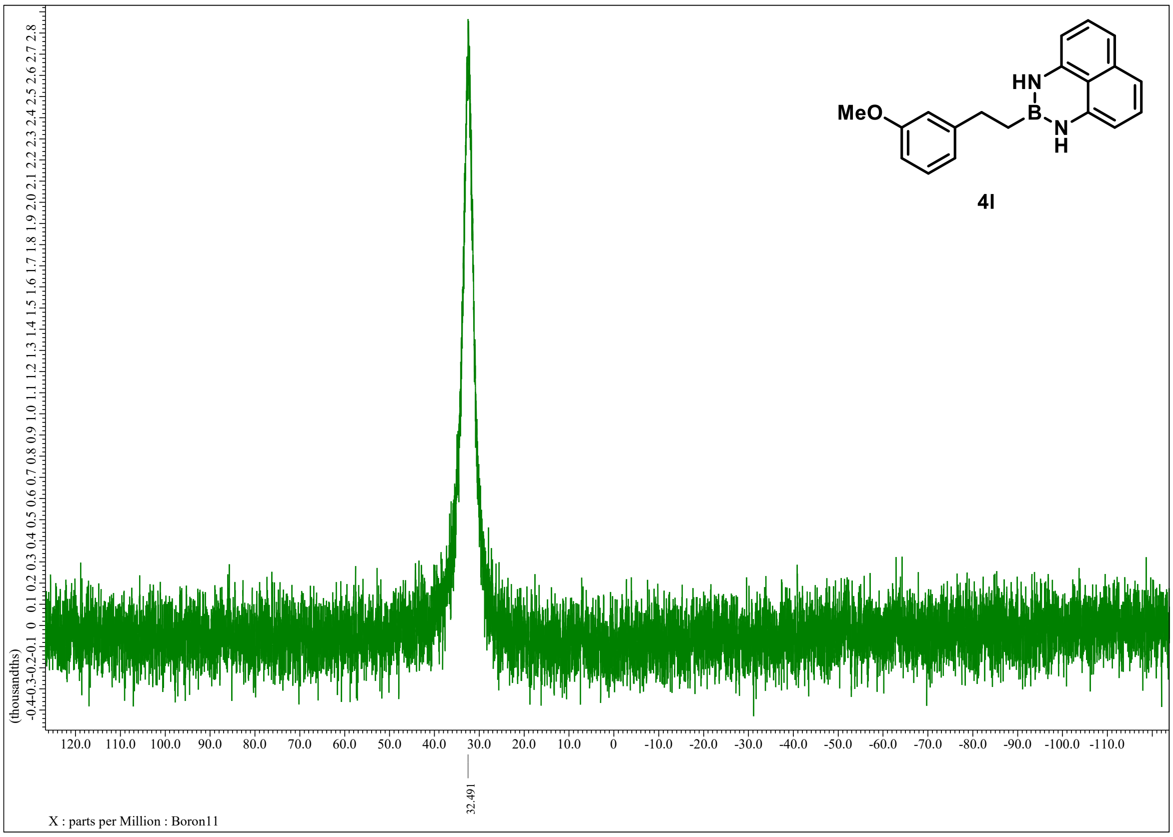

Figure S42. $^{11}B\{^{1}H\}$ NMR (128 MHz, $CDCl_3$) spectrum of **4l**.

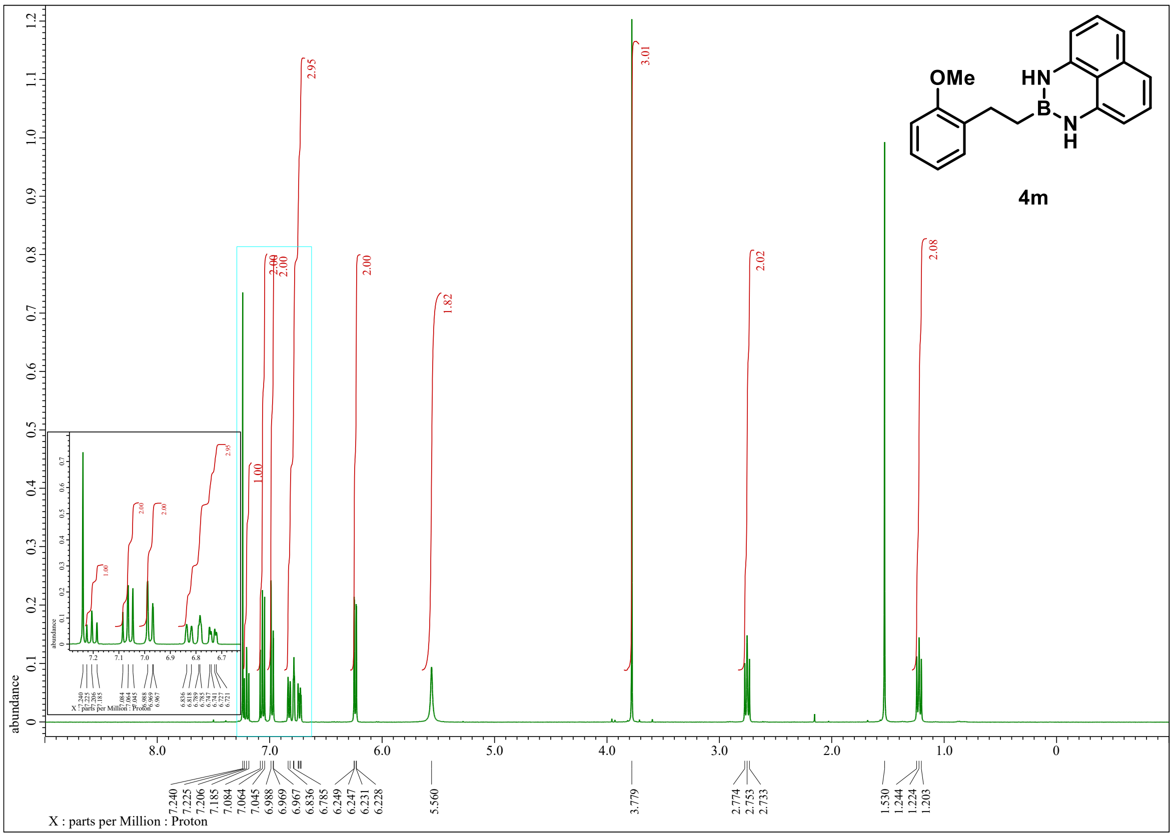

Figure S43. ^{1}H NMR (400 MHz, $CDCl_3$) spectrum of **4m**.

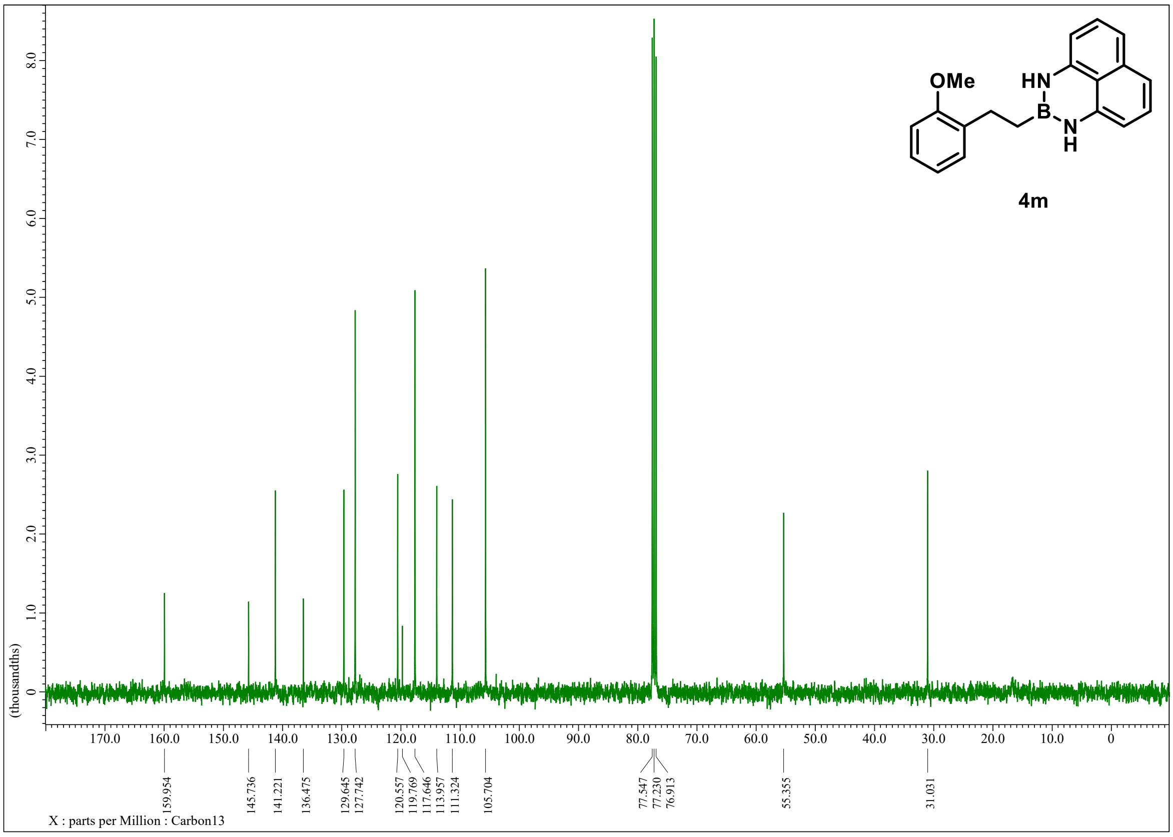

Figure S44. ^{13}C{^{1}H} NMR (100 MHz, $CDCl_3$) spectrum of **4m**.

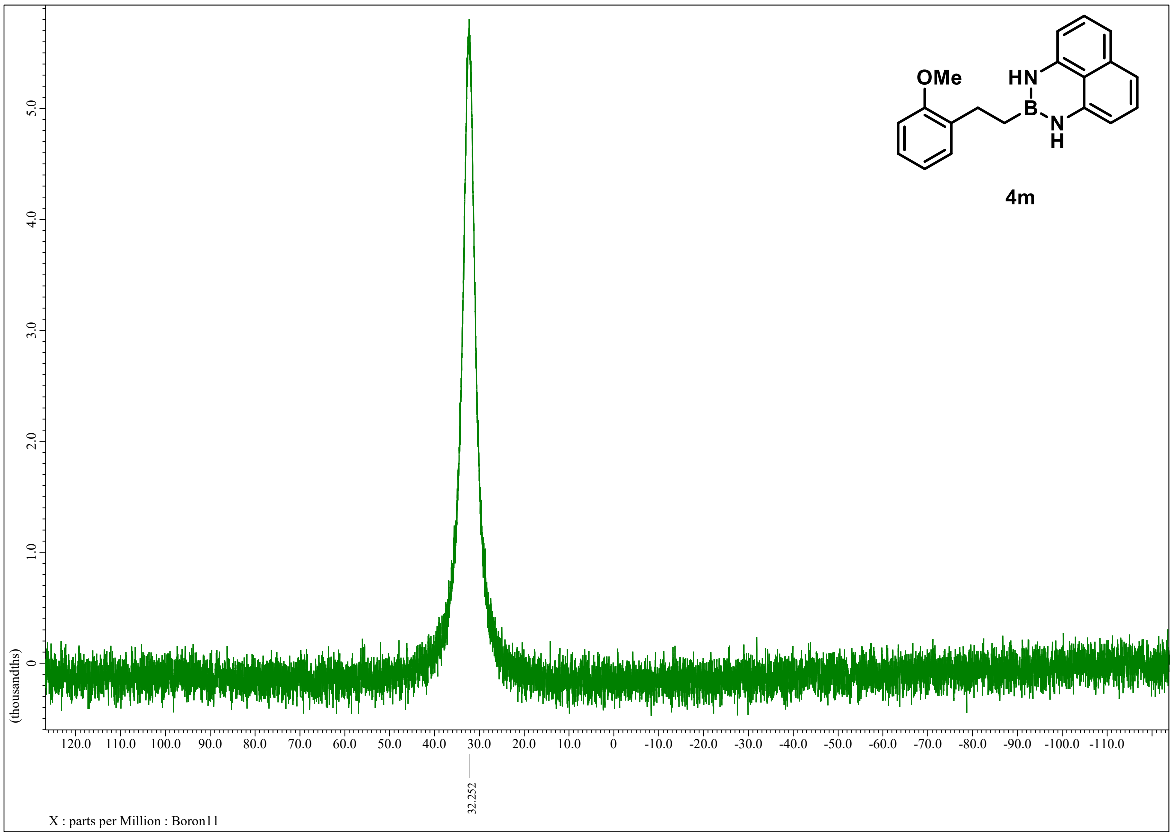

Figure S45. $^{11}B\{^{1}H\}$ NMR (128 MHz, $CDCl_3$) spectrum of **4m**.

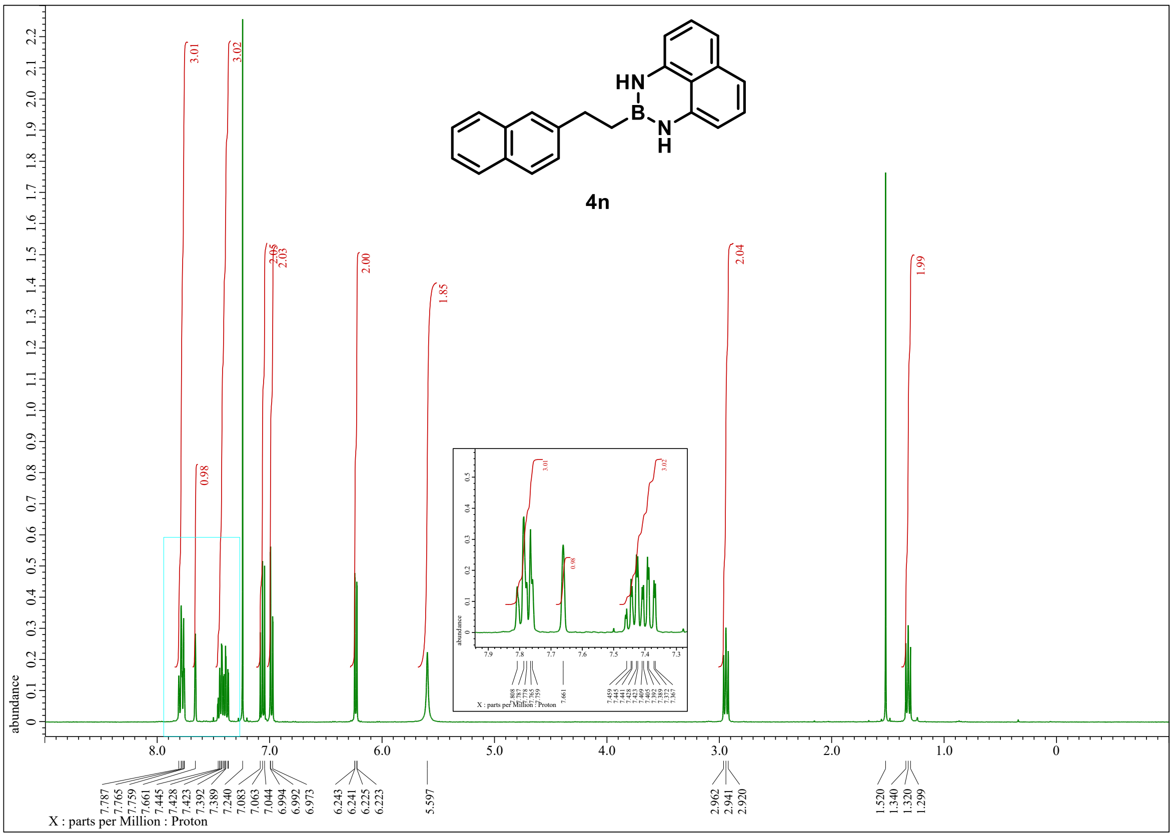

Figure S46. ^{1}H NMR (400 MHz, $CDCl_3$) spectrum of **4n**.

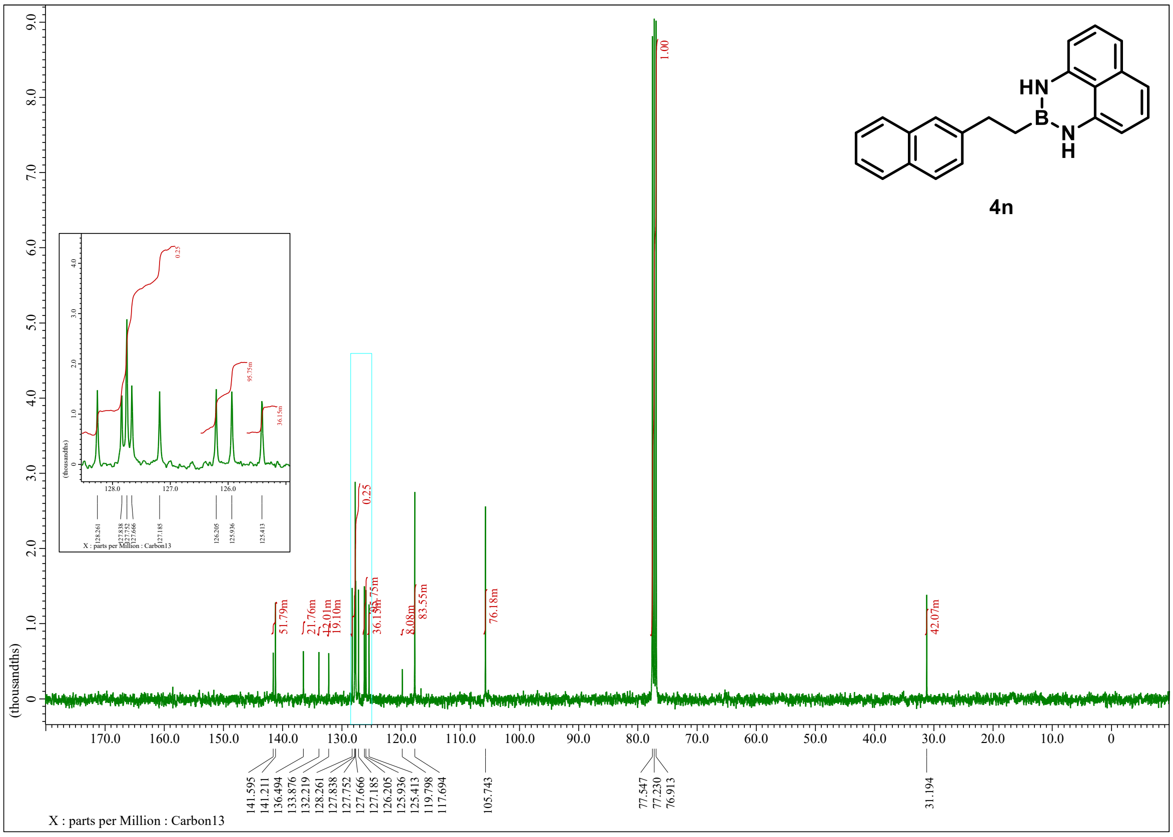

Figure S47. $^{13}C\{^{1}H\}$ NMR (100 MHz, $CDCl_3$) spectrum of **4n**.

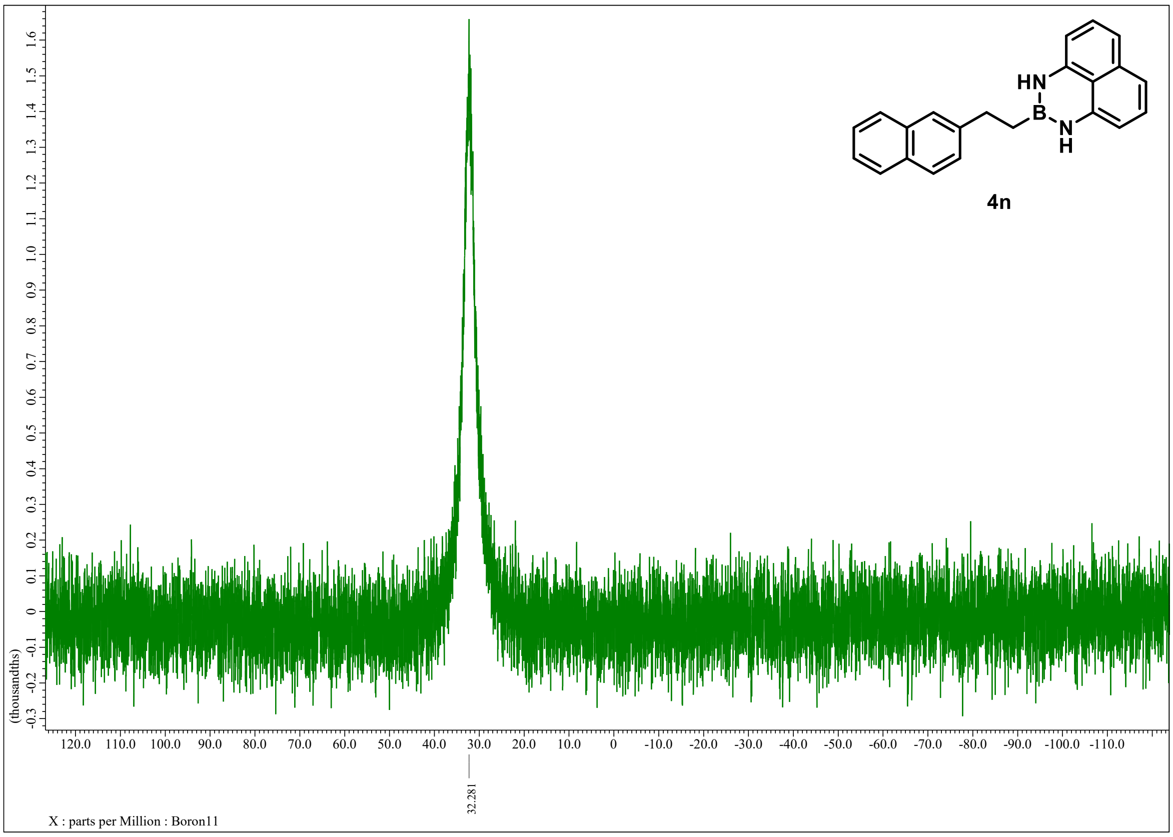

Figure S48. $^{11}B\{^{1}H\}$ NMR (128 MHz, $CDCl_3$) spectrum of **4n.**

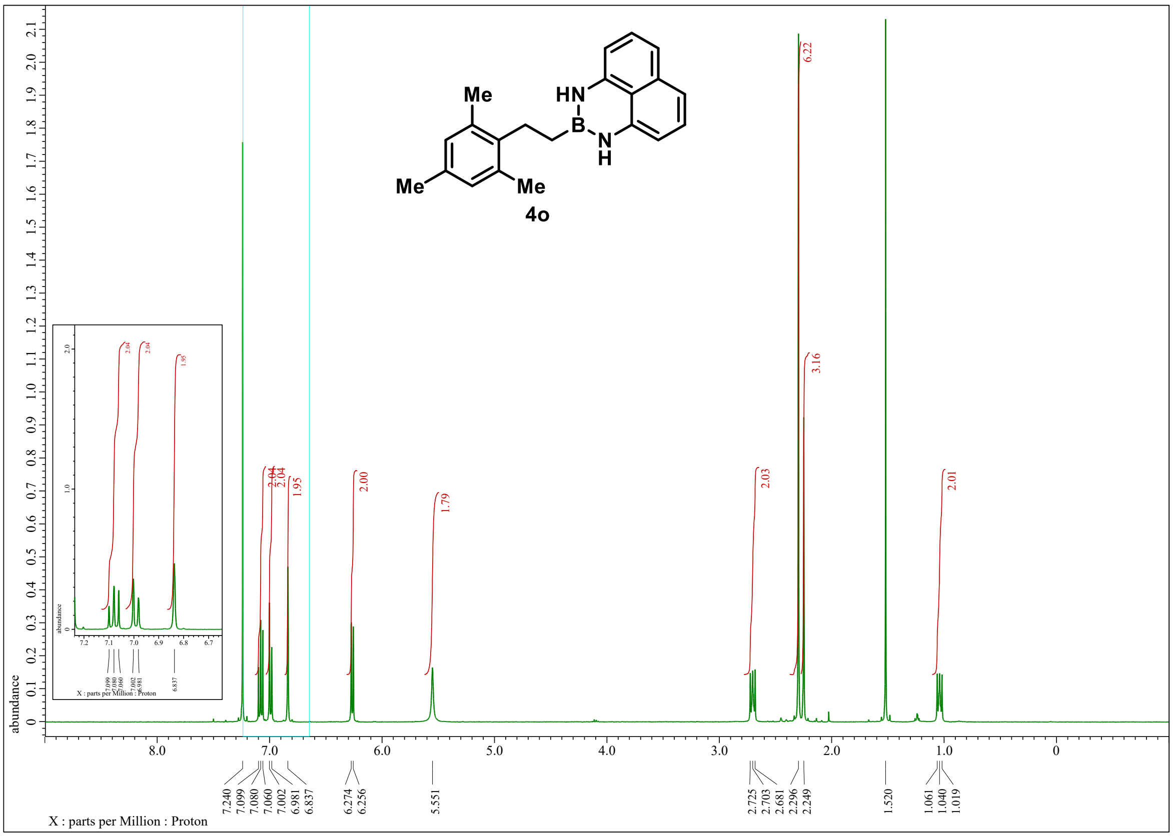

Figure S49. ^{1}H NMR (400 MHz, $CDCl_3$) spectrum of **4o.**

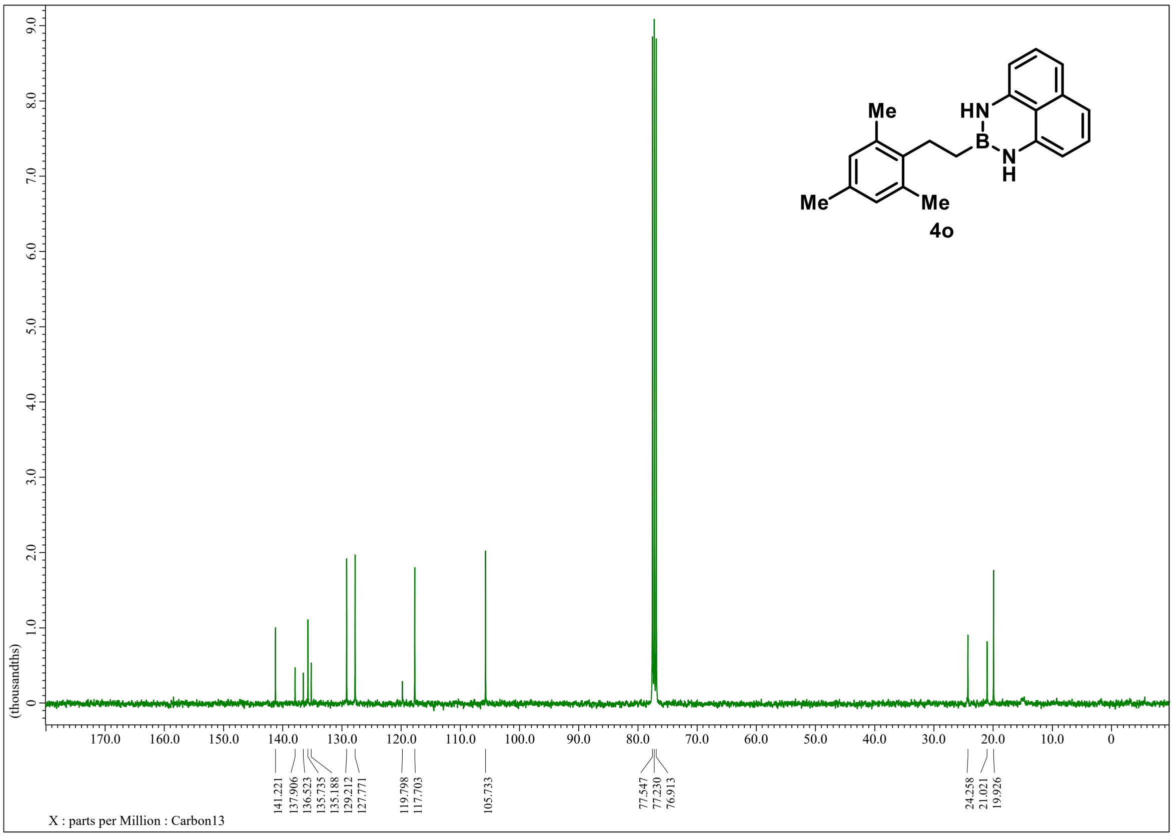

Figure S50. $^{13}C\{^{1}H\}$ NMR (100 MHz, $CDCl_3$) spectrum of **4o**.

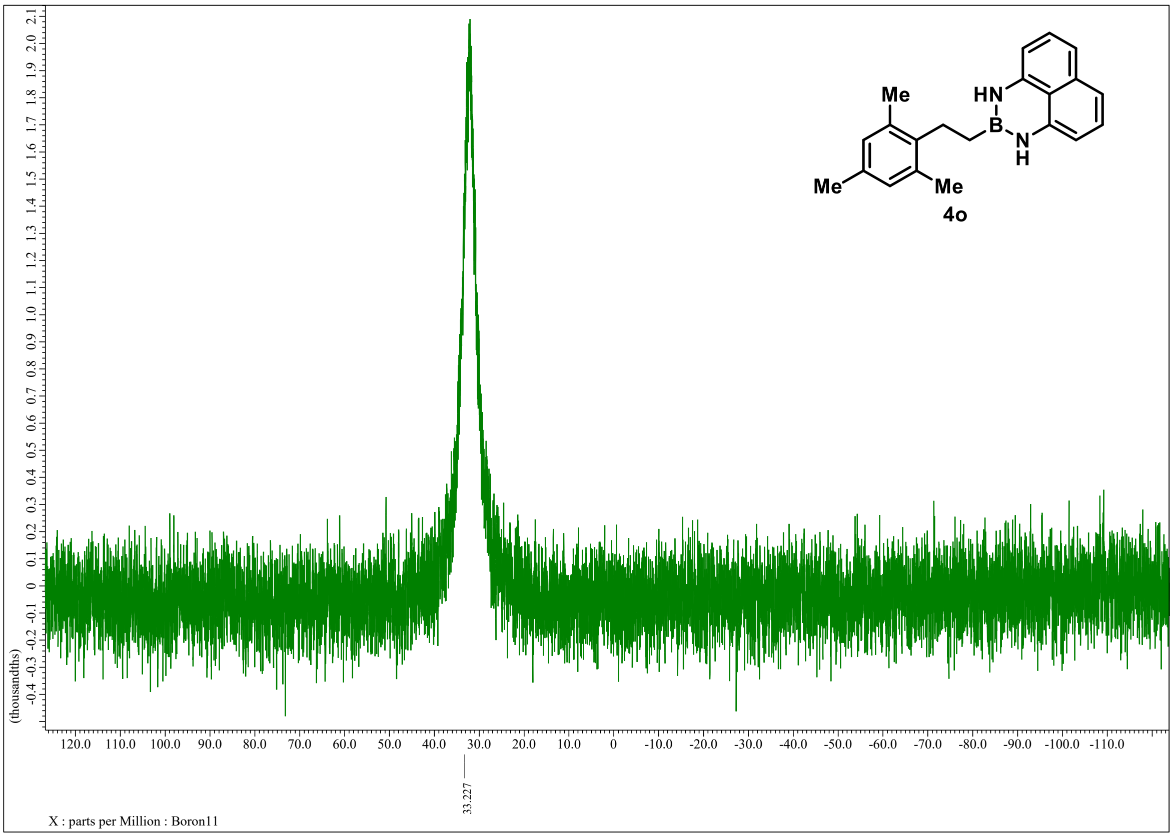

Figure S51. $^{11}B\{^1H\}$ NMR (128 MHz, $CDCl_3$) spectrum of **4o**.

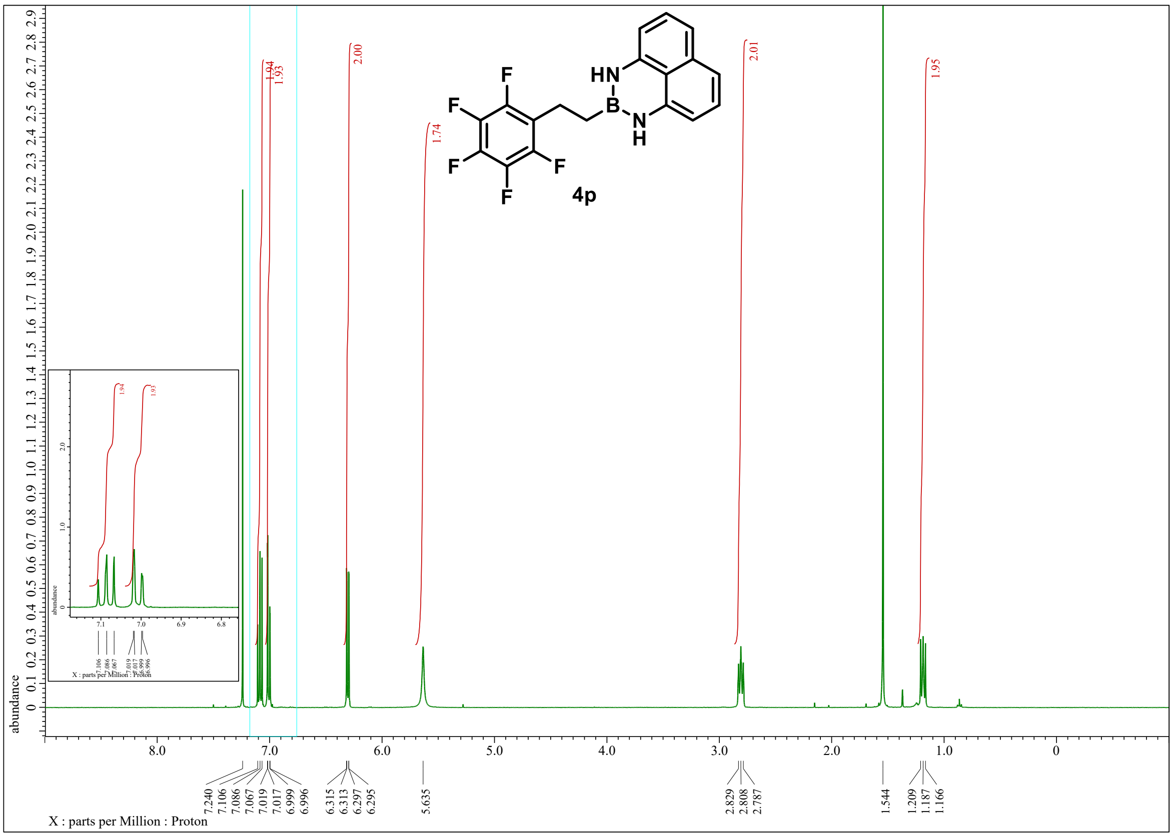

Figure S52. ^{1}H NMR (400 MHz, CDCl$_3$) spectrum of **4p**.

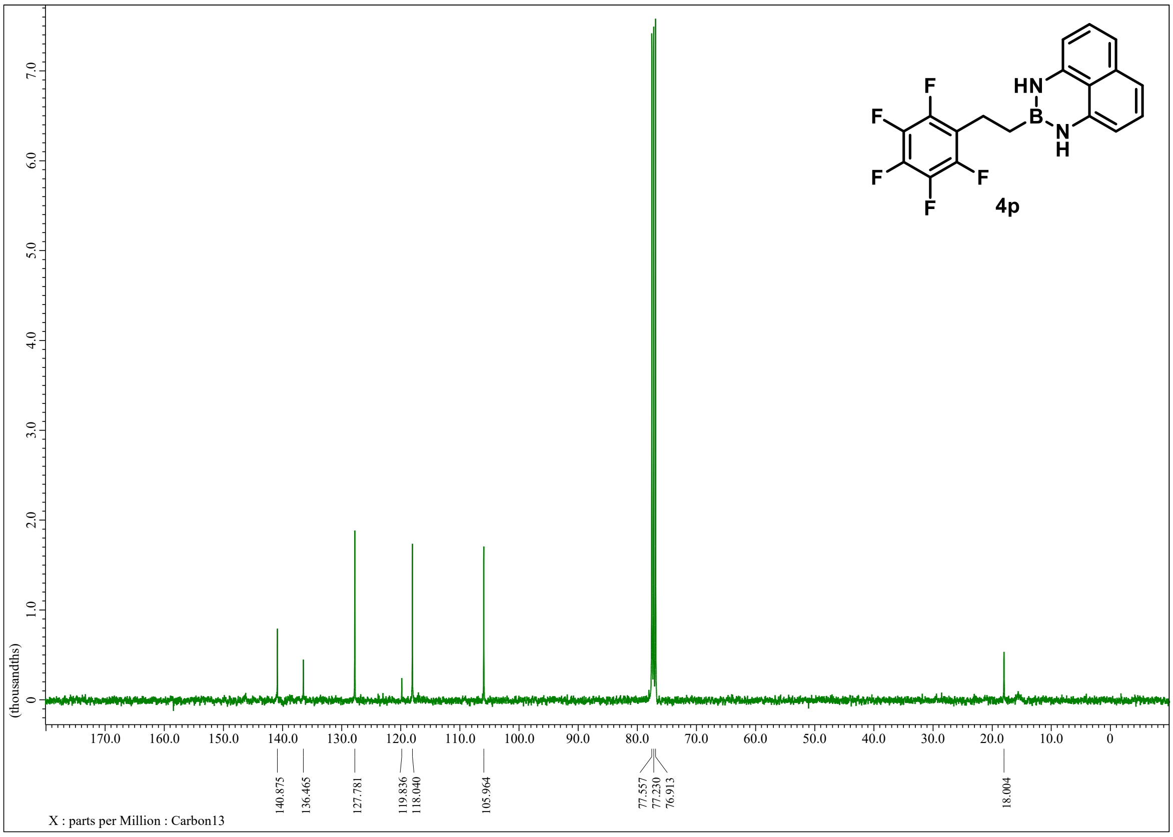

Figure S53. ^{13}C{^{1}H} NMR (100 MHz, CDCl$_3$) spectrum of **4p**.

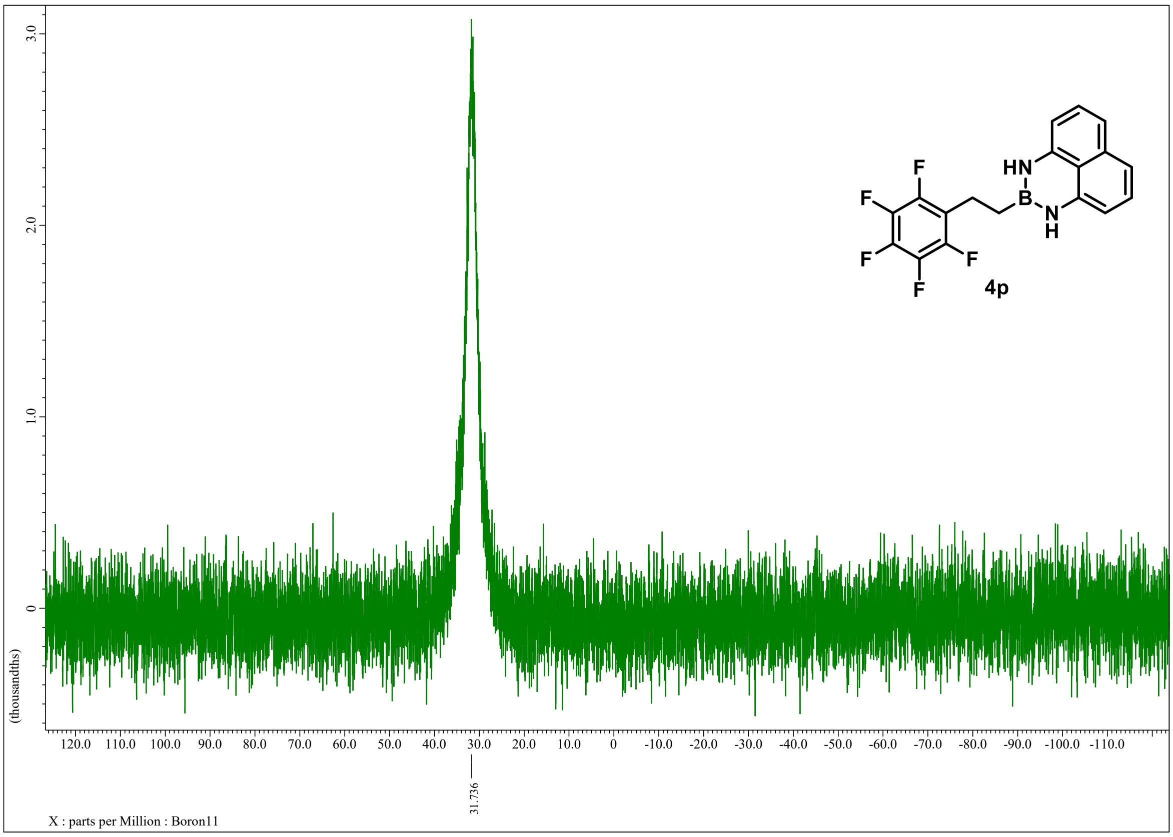

Figure S54. $^{11}B\{^{1}H\}$ NMR (128 MHz, $CDCl_3$) spectrum of **4p**.

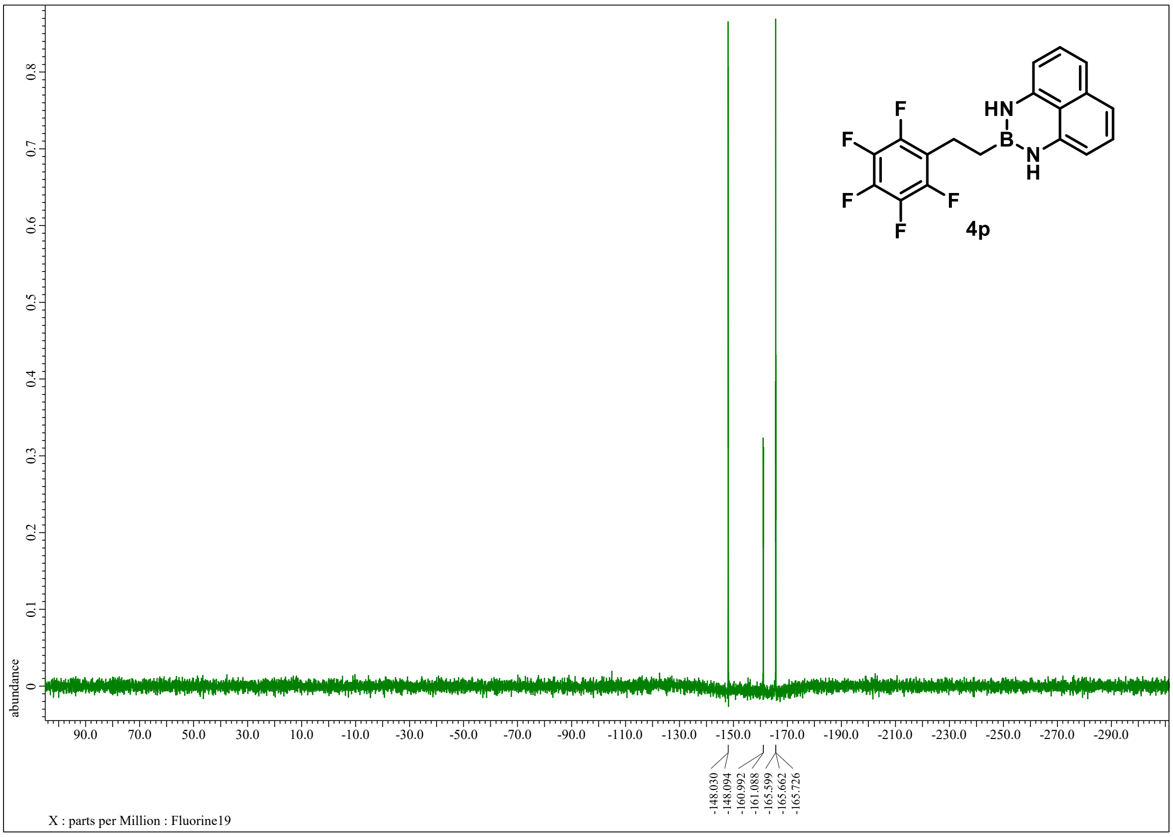

Figure S55. ^{19}F NMR (376 MHz, $CDCl_3$) spectrum of **4p**.

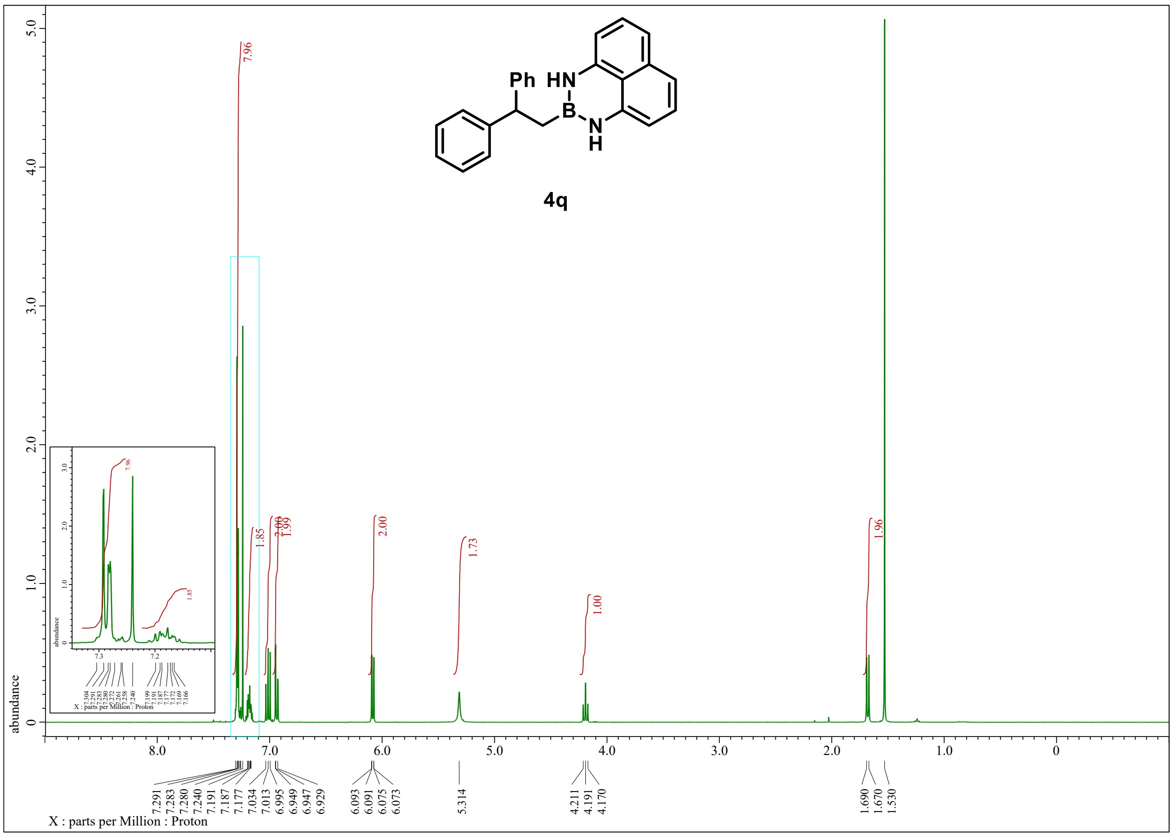

Figure S56. ^{1}H NMR (400 MHz, $CDCl_3$) spectrum of **4q**.

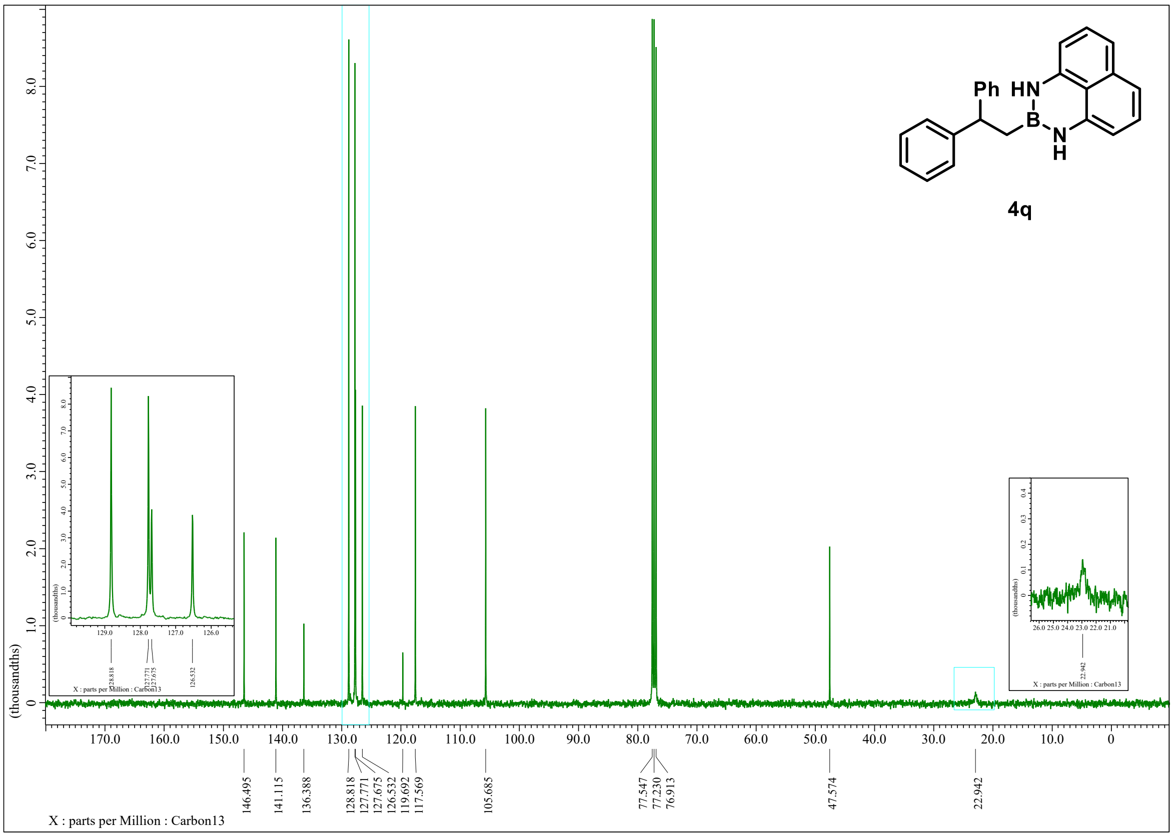

Figure S57. $^{13}C\{^{1}H\}$ NMR (100 MHz, $CDCl_3$) spectrum of **4q**.

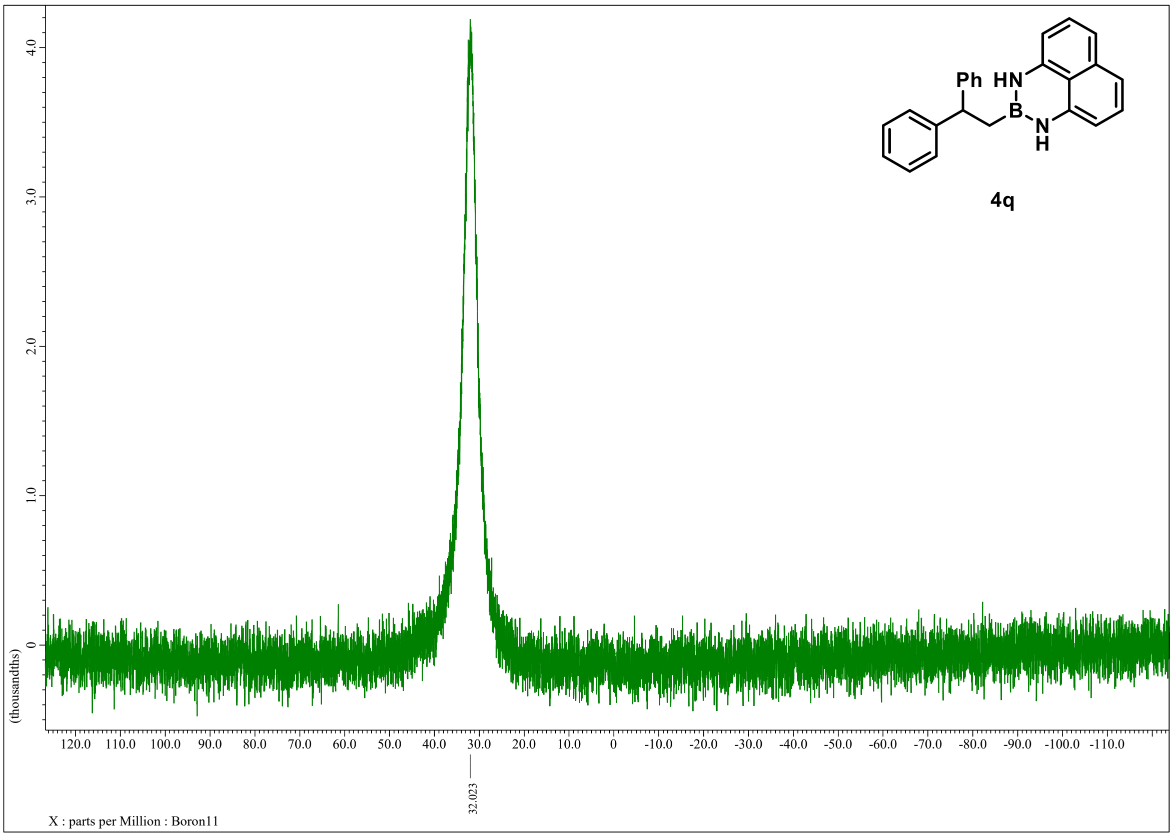

Figure S58. $^{11}B\{^{1}H\}$ NMR (128 MHz, $CDCl_3$) spectrum of **4q**.

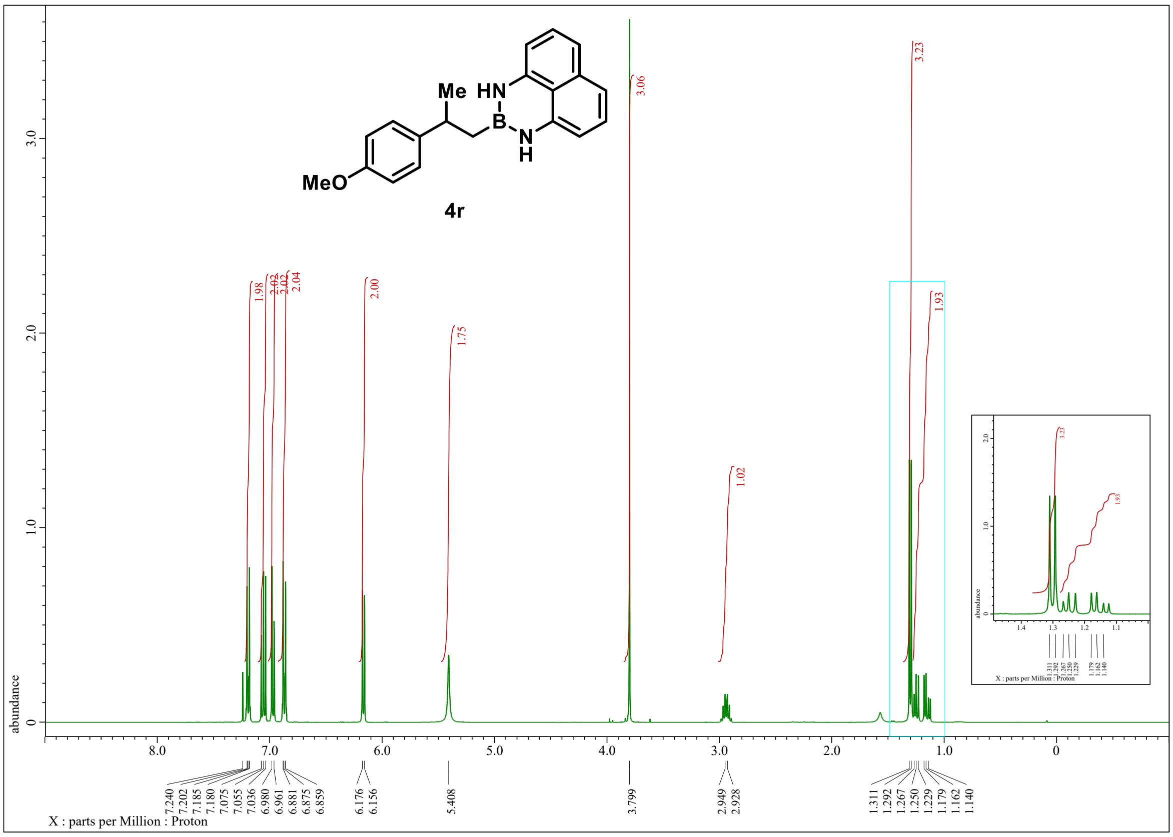

Figure S59. ^{1}H NMR (400 MHz, $CDCl_3$) spectrum of **4r**.

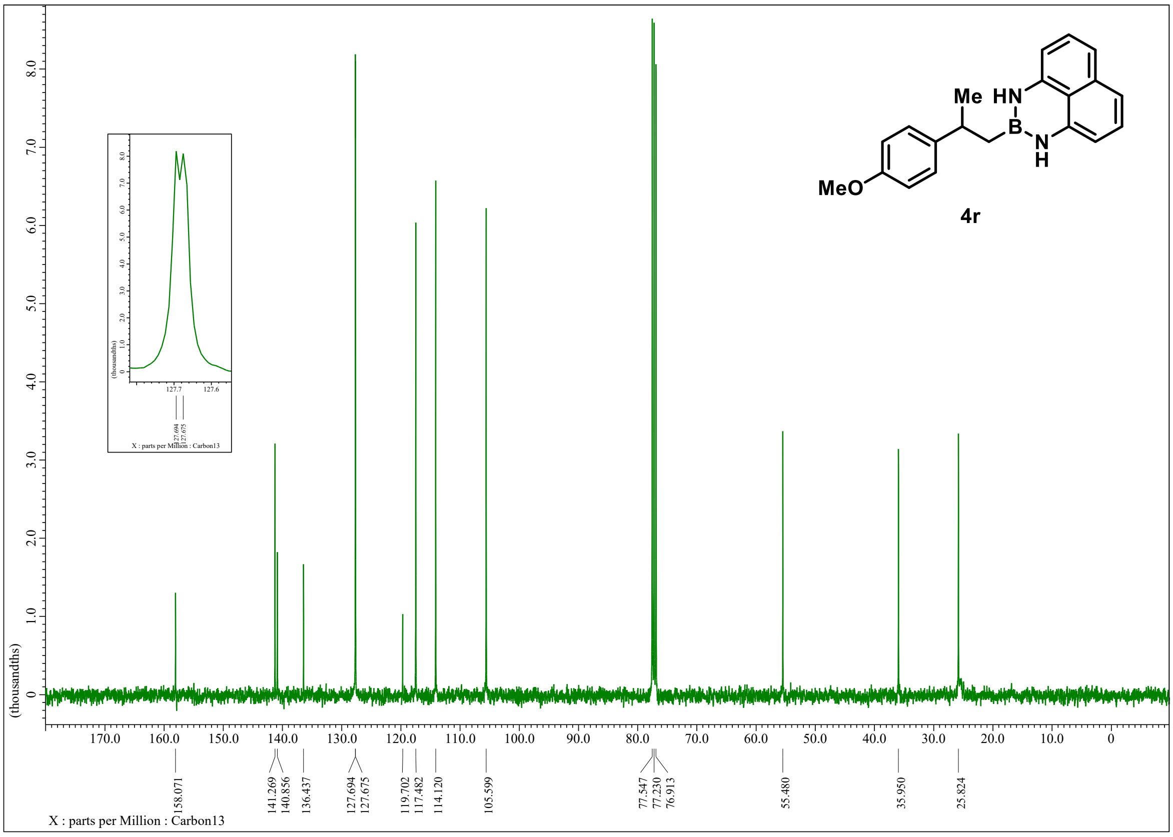

Figure S60. $^{13}C\{1H\}$ NMR (100 MHz, $CDCl_3$) spectrum of **4r**.

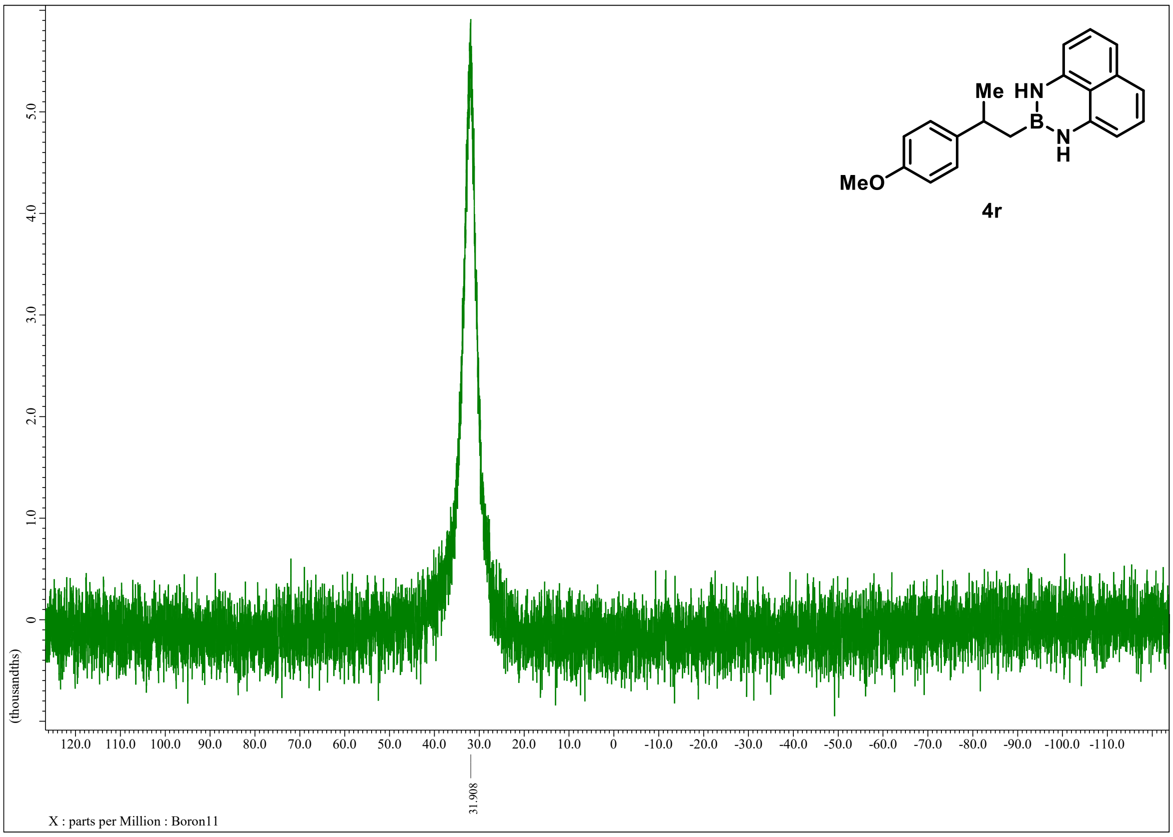

Figure S61. $^{11}B\{^{1}H\}$ NMR (128 MHz, $CDCl_3$) spectrum of **4r**.

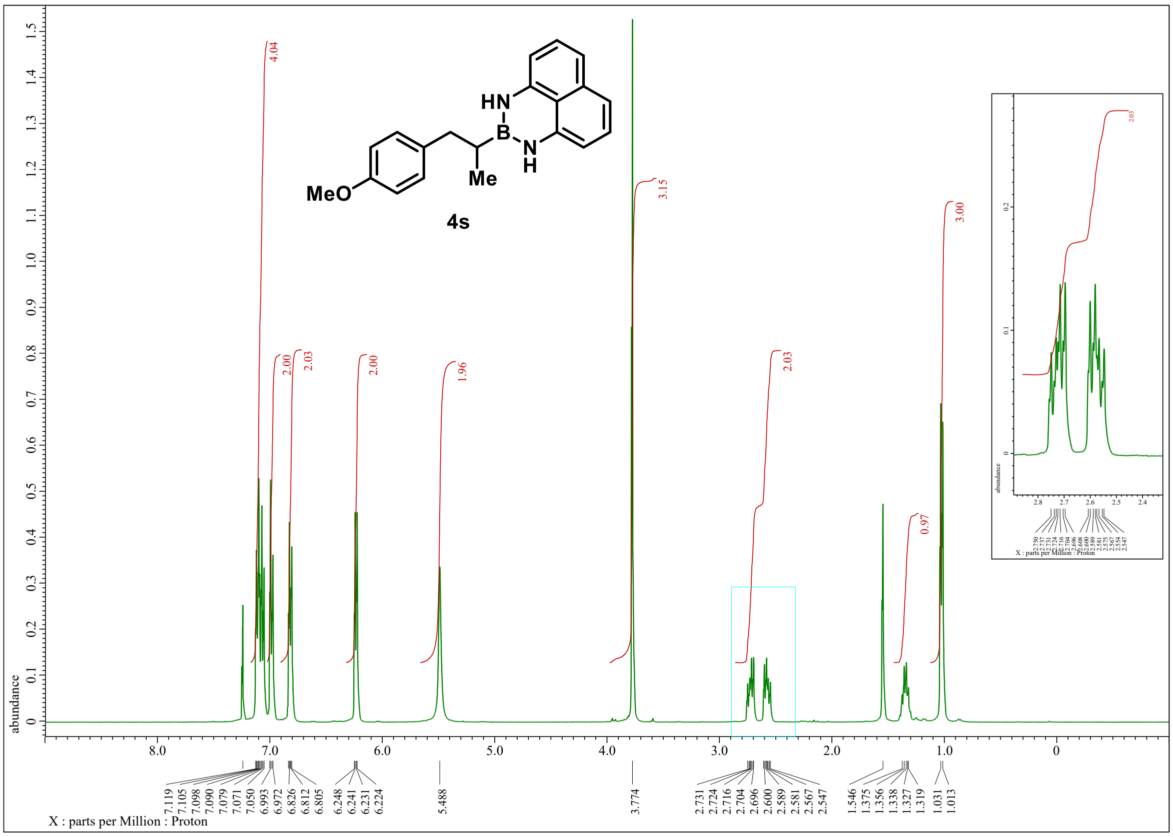

Figure S62. ^{1}H NMR (400 MHz, $CDCl_3$) spectrum of **4s**.

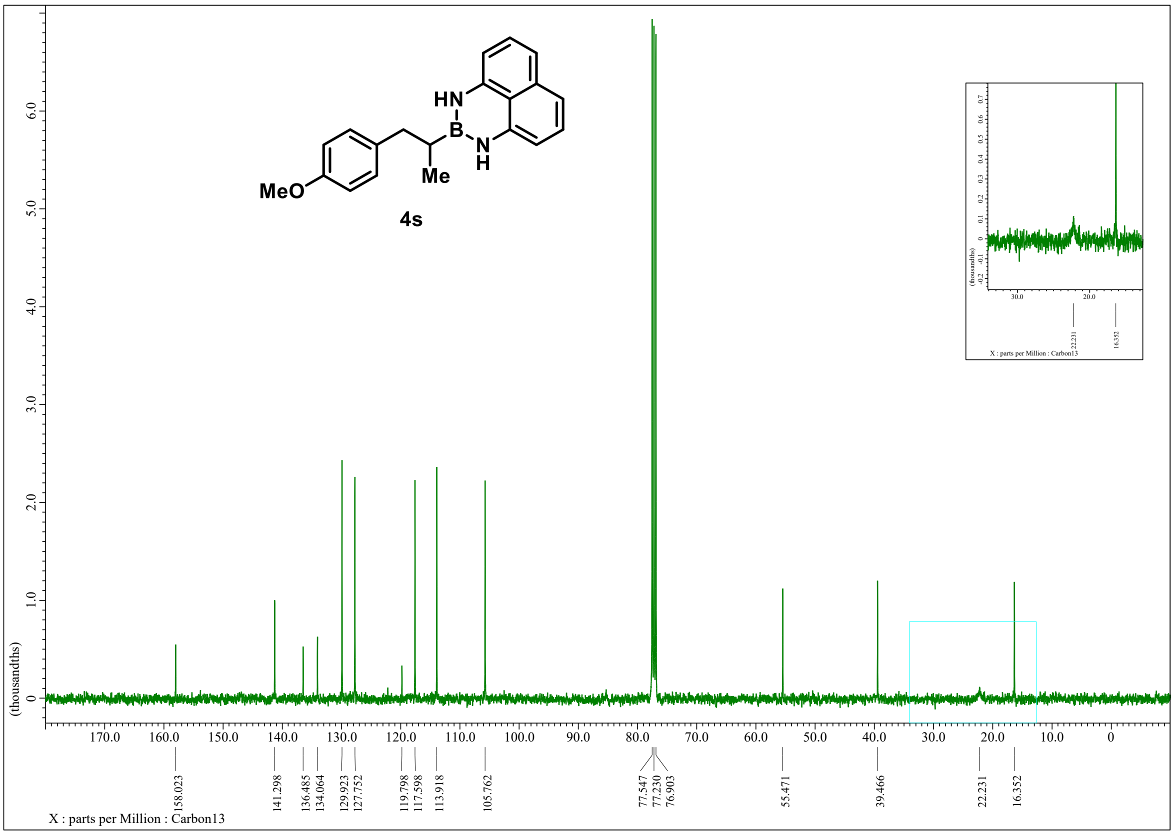

Figure S63. $^{13}C\{^1H\}$ NMR (100 MHz, $CDCl_3$) spectrum of **4s**.

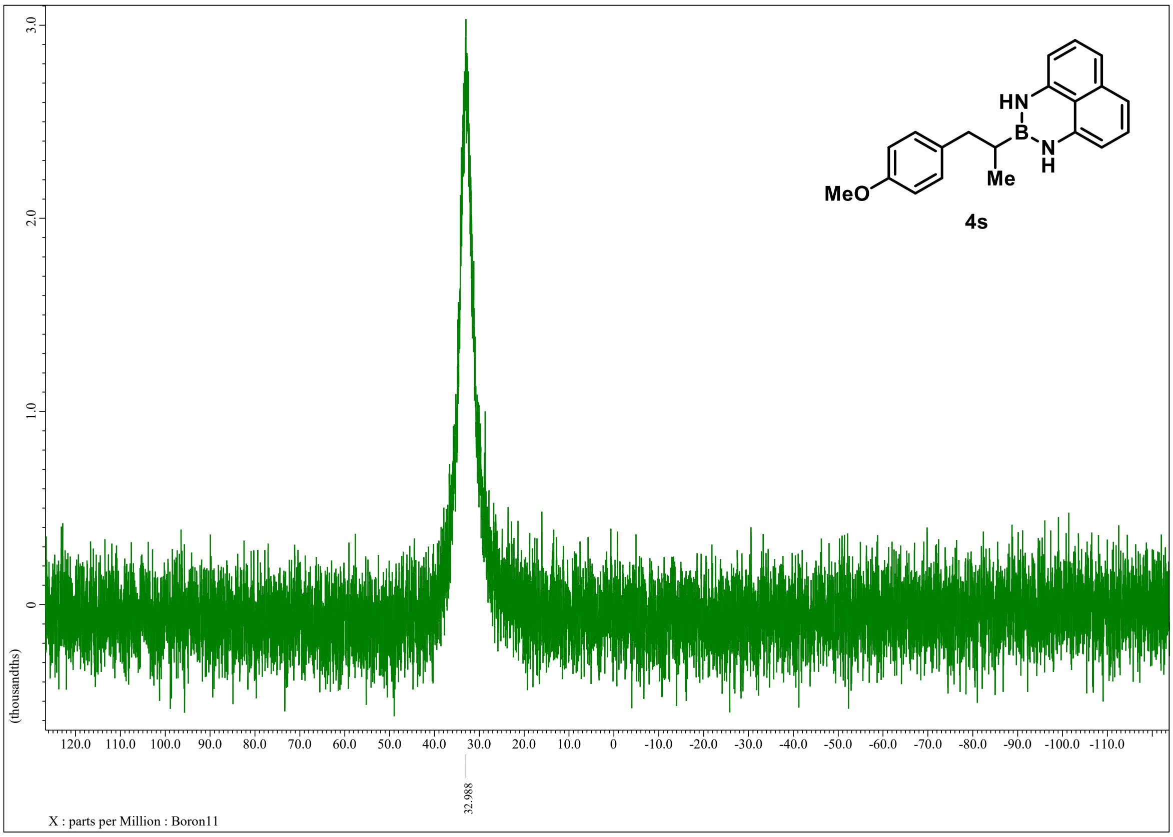

Figure S64. $^{11}B\{^{1}H\}$ NMR (128 MHz, $CDCl_3$) spectrum of **4s**.
